# Noncytotoxic polymyxin derivatives enhance antibiotic action against multidrug-resistant Gram-negative bacteria

**DOI:** 10.1128/aac.00712-25

**Published:** 2025-09-22

**Authors:** Danyel Ramirez, Danzel Marie Ramirez, Rajat Arora, Gilbert Arthur, Frank Schweizer

**Affiliations:** 1 Department of Chemistry, University of Manitoba 8664, Winnipeg, Manitoba, Canada; 2 Department of Biochemistry and Medical Genetics, University of Manitoba 646291, Winnipeg, Manitoba, Canada; 3 Department of Medical Microbiology and Infectious Diseases, University of Manitoba 574854, Winnipeg, Manitoba, Canada; University of Fribourg, Fribourg, Switzerland

**Keywords:** antibiotic potentiator, Gram-negative bacteria, antibiotic adjuvant, polymyxin

## Abstract

The widespread emergence of multidrug-resistant (MDR) Gram-negative bacteria prompted the reintroduction of polymyxins in the clinic despite their adverse effects. Ongoing research is primarily focused on the development of non-nephrotoxic and -neurotoxic polymyxins as not only standalone agents but also as potentiators that enhance the activity of a partner antibiotic. Safer derivatives of polymyxin B_3_, a minor component of polymyxin B, were synthesized and utilized as a potentiator of multiple antibiotics. Compound **1**, consisting of Dap residues, was nontoxic to kidney cells and is a promising outer membrane permeabilizer that synergized with six different classes of antibiotics against MDR Gram-negative bacteria. Compound **1** extended the activity spectrum of rifampicin, zoliflodacin, and pristinamycin by lowering the minimum inhibitory concentrations of these antibiotics below their interpretative susceptibility breakpoints in MDR *Pseudomonas aeruginosa*, *Acinetobacter baumannii*, *Escherichia coli*, *Klebsiella pneumoniae*, and *Enterobacter cloacae*. Notably, the novel combination of zoliflodacin, a first-in-class antibiotic in phase III trials for gonorrhea, and compound **1** exhibited potent bactericidal activity in MDR *P. aeruginosa* and *A. baumannii*.

## INTRODUCTION

The increasing prevalence of multidrug-resistant (MDR) bacteria has brought the world to the threshold of a post-antibiotic era. The emergence of new resistance mechanisms poses a significant threat to the effective treatment of common infections, leading to prolonged illnesses and rising mortality rates (1). Moreover, the success of critical medical procedures, including major surgeries, cancer chemotherapy, and organ transplantations, is at risk due to the diminishing potency of antibiotics (2). Without targeted interventions, drug-resistant infections are projected to cause approximately 10 million annual deaths by 2050, with an estimated global economic cost of $100 trillion (3).

One approach to circumvent antibiotic resistance is through the development of antibiotic potentiators that can restore or enhance the effect of existing antibiotics (4, 5). These “helper molecules” possess little to no antibacterial activity but may sensitize bacteria to a partner antibiotic by overcoming resistance mechanisms (4, 5). Antibiotic potentiators can inhibit efflux pumps, inhibit antibiotic degradation by enzymes, or improve membrane permeability, among other modes of action (4, 5). Importantly, these molecules are advantageous due to their low probability of resistance development (4, 5). Since antibiotic potentiators also allow antibiotics to be more effective at sub-optimal concentrations, dose-dependent toxicity associated with these antibiotics can be mitigated (4). In certain cases, these molecules may even extend the activity spectrum of antibiotics by allowing Gram-positive bacteria-selective agents to be used against Gram-negative bacteria (4).

Broadening the activity spectrum to include Gram-negative bacteria is critical, as infections caused by Gram-negative bacteria are particularly more challenging to treat due to the presence of an outer membrane that acts as a formidable permeability barrier (4). The outer membrane confers intrinsic resistance to antibiotics that are otherwise effective against Gram-positive bacteria (4). To overcome this barrier and enhance intracellular antibiotic accumulation, outer membrane permeabilizers have been developed as a potential strategy to improve treatment outcomes (4, 5).

Polymyxins serve as a representative class of molecules capable of permeabilizing bacterial membranes (6, 7). These cyclic lipopeptides are synthesized as secondary metabolites by *Paenibacillus polymyxa* as a mixture of structurally related derivatives that exhibit slight variations in amino acid sequence and lipid composition (6, 7). Polymyxins consist of a heptapeptide cyclic core and a linear tripeptide attached to an *N-*terminal fatty acid (6, 7). Polymyxin B (PMB) and E (colistin), which are the only clinically used polymyxins, are distinct from each other by one amino acid at position 6 (PMB = D-phenylalanine (D-Phe), colistin = D-leucine (D-Leu)) (6, 7). Commercial forms of PMB and colistin consist of multiple components, with the major constituents of PMB (PMB_1_ and PMB_2_) and colistin (colistin A and B) comprising branched lipids that differ by a single methylene group (6, 7). Although polymyxins demonstrate potent antibacterial activity, these molecules can also function as antibiotic potentiators (6).

The amphiphilic nature of polymyxins, conferred by both basic and hydrophobic moieties, is directly tied to the ability of these molecules to improve the effect of co-administered antibiotics. Specifically, the basic 2,4-diaminobutyric acid (Dab) residues of polymyxins electrostatically interact with the negatively charged phosphates of lipid A within the lipopolysaccharide (LPS) of the Gram-negative bacterial outer membrane (6). This interaction displaces divalent cations (Mg^2+^ and Ca^2+^) that bridge adjacent LPS molecules, thereby destabilizing the outer membrane (6). Concurrently, the hydrophobic regions of polymyxins associate with the fatty acyl chains of lipid A, further compromising membrane integrity (6). The combined electrostatic and hydrophobic interactions result in outer membrane permeabilization, ultimately promoting the intracellular accumulation of a partner antibiotic (6).

Numerous polymyxin derivatives devoid of intrinsic activity have been reported to sensitize Gram-negative bacteria. A prominent example is polymyxin B nonapeptide (PMBN), which lowered the minimum inhibitory concentration (MIC) of a wide variety of antibiotics (8). More recently, SPR741, a PMBN derivative, completed phase I trials as part of a combination regimen with β-lactam antibiotics (9, 10). In clinical settings, PMB and colistin have been employed in dual and even in triple combination therapies, especially against MDR and extensively drug-resistant (XDR) bacteria (11–16).

However, due to concerns of nephrotoxicity and neurotoxicity, polymyxins are reserved for infections with limited treatment options (6, 7). The same structural features that render polymyxins active against Gram-negative bacteria also predispose polymyxins to interactions with human kidney and neural cells (6). Thus, the overall hydrophobicity and positive charge of polymyxins were modulated to improve safety profile, without compromising outer membrane permeabilizing capacity. In this work, a total of four derivatives were synthesized with PMB_3_ as the base scaffold. Lead compound **1**, in which all the Dab residues were substituted with its shorter analog, 2,3-diaminopropionic acid (Dap), was found to be nontoxic against human kidney cell lines HK-2 and renal proximal epithelial tubule cells (RPTEC/TERT1). Compound **1** exhibited selective antibacterial activity against colistin-susceptible Enterobacterales, including *Escherichia coli* and *Enterobacter cloacae*. Additionally, compound **1** consistently demonstrated synergy with multiple antibiotics, such as rifampicin, zoliflodacin, and pristinamycin, against MDR *Pseudomonas aeruginosa*, *Acinetobacter baumannii*, *Escherichia coli*, *Klebsiella pneumoniae*, and *Enterobacter cloacae*. Compound **1** effectively reduced the MIC of the tested antibiotics below their interpretative susceptibility breakpoints and was remarkably bactericidal in combination with zoliflodacin, a novel anti-*Neisseria gonorrhoeae* antibiotic, against MDR *P. aeruginosa* and *A. baumannii*.

## RESULTS

### Design and synthesis of polymyxin derivatives

Structure-activity relationship studies have shown that decreasing the overall hydrophobicity of polymyxins led to reduced toxicity. Indeed, a reduction in toxicity was observed when individual Dab residues were previously replaced with their shorter counterpart, Dap, which has one less methylene in the side chain (17–22). Therefore, we postulated that toxicity can be further reduced by substituting all Dab residues with Dap.

Previous work has also shown that decreasing the net positive charge of polymyxins led to reduced toxicity (23, 24). Because Dab^3^ is not conserved in all naturally derived polymyxins, this amino acid may be amenable to modifications (20). Moreover, an alanine scan established that Dab residues in the linear portion of the molecule (Dab^1,3^) contribute less to antibacterial activity, in comparison to Dab residues in the cyclic core (Dab^5,8,9^) (25). Thus, Dab^3^ will also be replaced with aspartic acid (Asp) to achieve a decrease in net positive charge. A negatively charged residue, such as Asp, was selected with the objective of attenuating electrostatic interactions with the anionic brush border membrane of the kidney proximal tubules. The binding of polymyxins with the brush border membrane is responsible for polymyxin reabsorption and accumulation in the kidneys, which subsequently leads to nephrotoxicity (26).

Finally, several papers have reported that conversion of amines into the more basic guanidines (higher pKa) augmented outer membrane permeabilization, which translated to greater antibiotic-potentiating activity (27–29). Therefore, all Dap amines will also be converted to guanidines (GDap).

**Fig 1:**
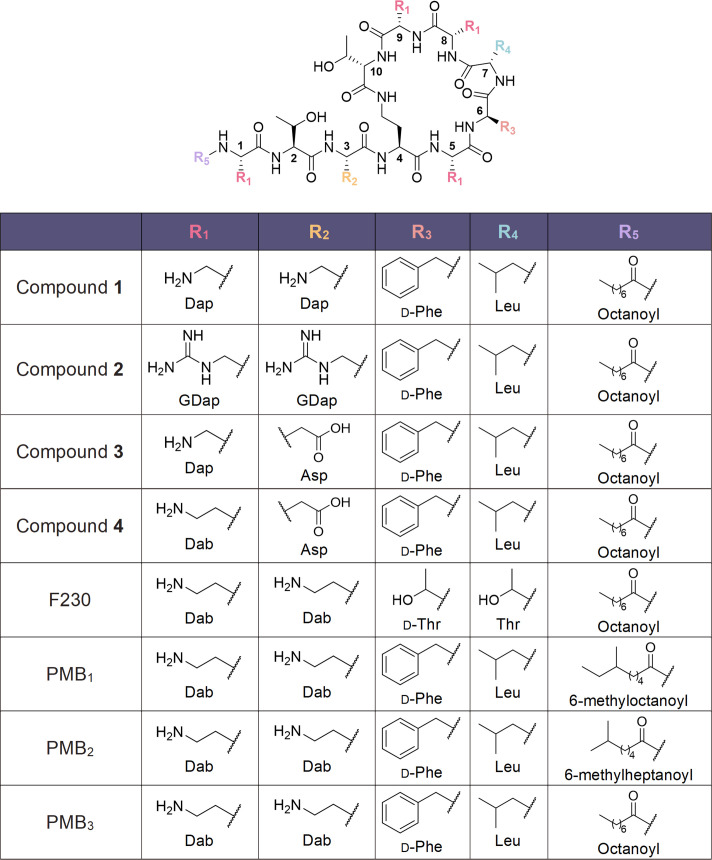
Chemical structures of synthesized and control polymyxin derivatives.

A total of four PMB_3_ derivatives were prepared with modified hydrophobicity, positive charge, and basicity (Fig. 1). PMB_3_ was selected as the base scaffold since it comprises an unbranched octanoyl lipid that is more readily available. To decrease hydrophobicity, all 5 Dab residues of PMB_3_ were replaced with Dap to produce compound **1**. To improve outer membrane permeabilizing capacity, the amines of compound **1** were converted to guanidines to produce compound **2**. To decrease both hydrophobicity and positive charge, the 5 Dab residues of PMB_3_ were replaced with 4 Dap residues and 1 Asp to produce compound **3**. Lastly, to study the influence of just decreased positive charge alone, Dab^3^ was replaced with Asp, preserving the four other Dab residues, to produce compound **4**.

**Fig 2:**
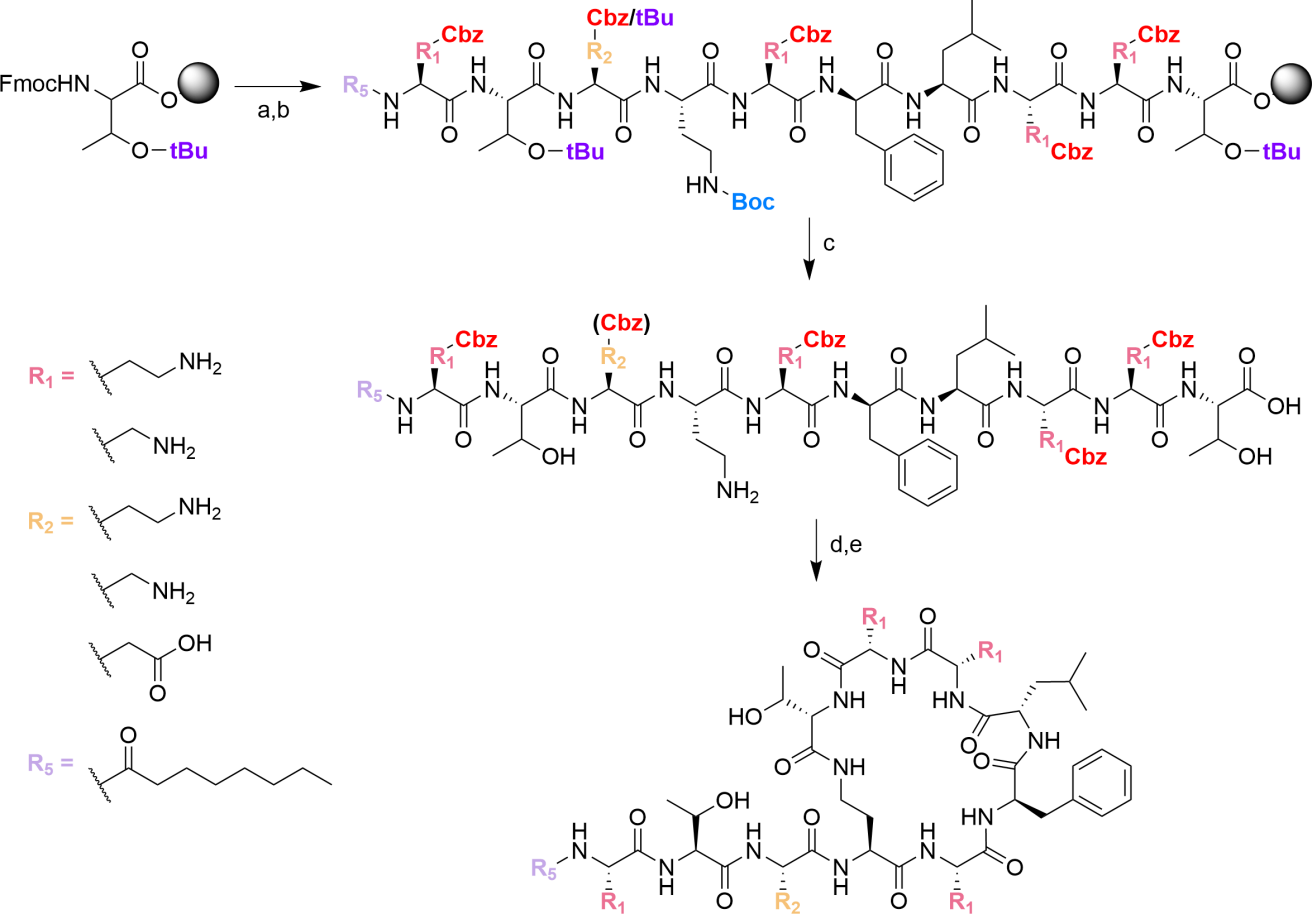
Synthesis of compounds 1, 3, and 4. Reagents and conditions: (a) 4:1 *N,N*-dimethylformamide (DMF)/piperidine; (b) protected amino acid or lipid, 2-(1H-benzotriazole-1-yl)−1,1,3,3-tetramethylaminium tetrafluoroborate (TBTU), *N*-methylmorpholine (NMM); (c) 95:5 trifluoroacetic acid (TFA)/H_2_O; (d) benzotriazol-1-yloxytripyrrolidinophosphonium hexafluorophosphate (PyBOP), 1-hydroxybenzotriazole (HOBt), NMM; (e) 4:5:1 methanol (MeOH)/acetic acid (AcOH)/H_2_O, H_2_ gas, 20% Pd/C.

**Fig 3:**
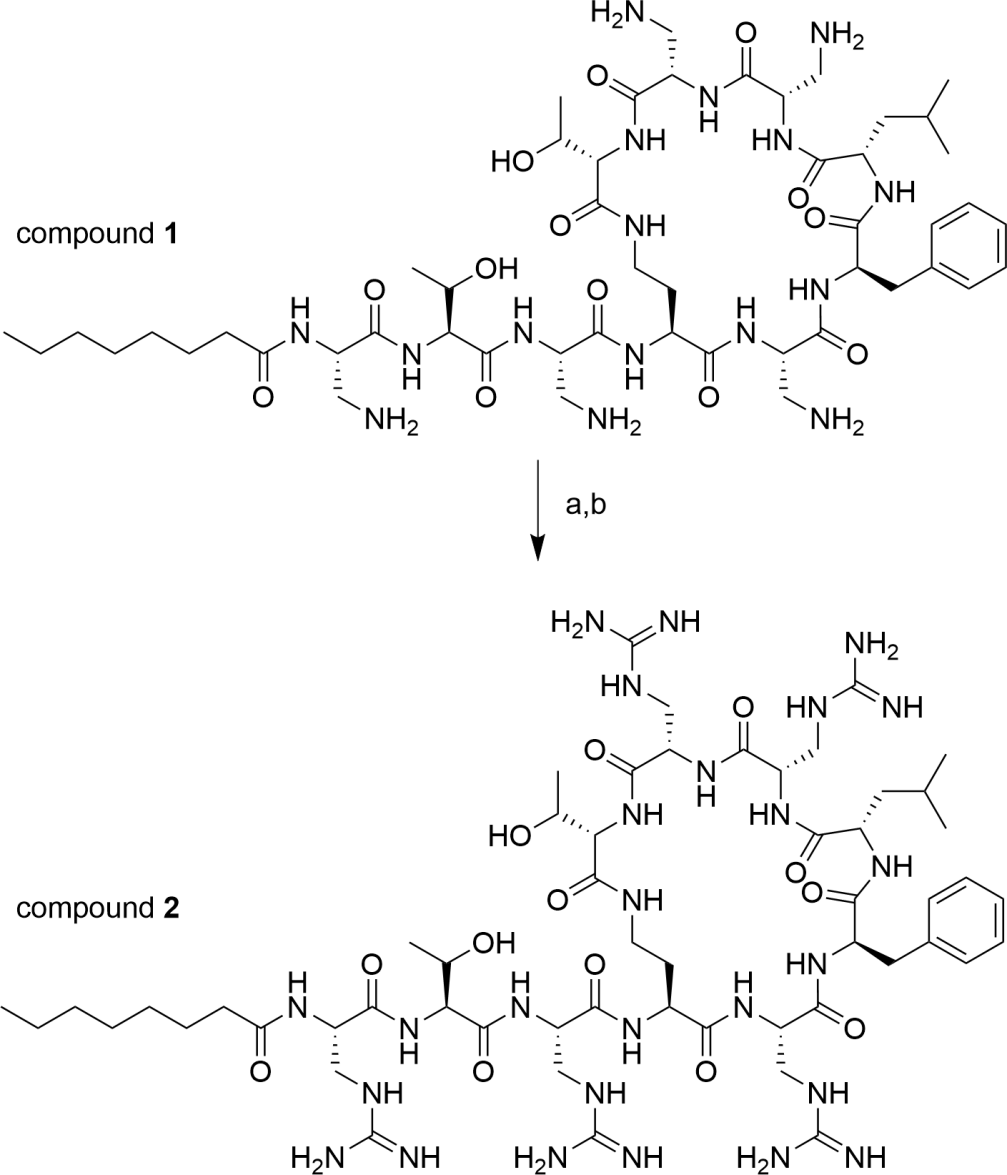
Synthesis of compound 2 via guanidinylation of compound 1. Reagents and conditions: (a) *N,N*-di-Boc-triflylguanidine, triethylamine (TEA), 3:1 dioxane/H_2_O; (b) 1:1 TFA/dichloromethane (DCM).

The linear protected peptides were prepared via solid-phase peptide synthesis (SPPS), cyclized in solution, and benzyloxycarbonyl (Cbz)-deprotected via hydrogenolysis (Fig. 2). Compound **2** was produced by guanidinylating and subsequently *tert*-butoxycarbonyl (Boc)-deprotecting compound **1** (Fig. 3). The molecular weights of the compounds are listed in Table S1, whereas the nuclear magnetic resonance (NMR) spectra are shown in Fig. S1 to S25 of the supplemental material.

### Cytotoxicity of polymyxin derivatives in kidney cell lines

**Fig 4:**
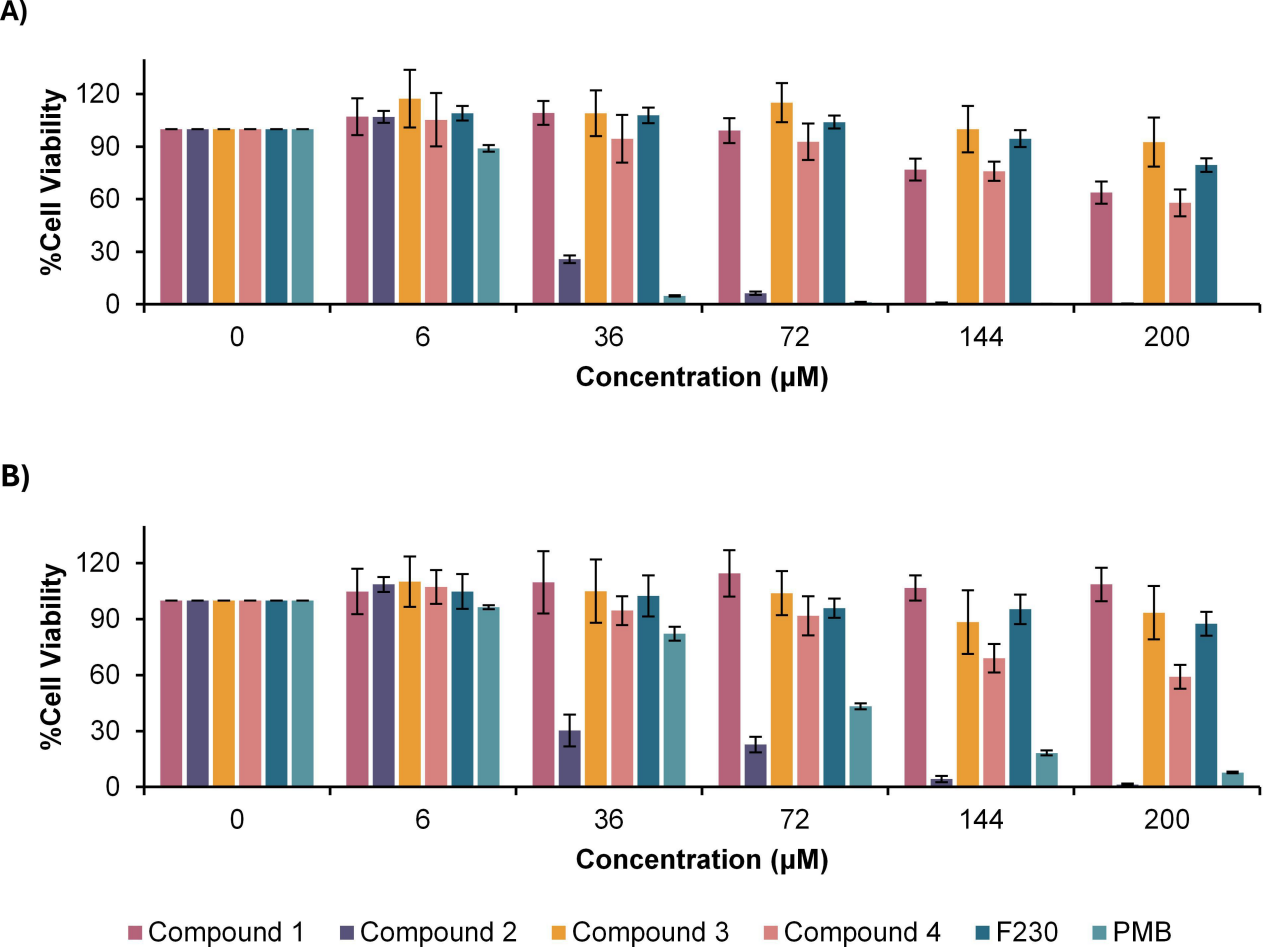
Viability of kidney cell lines (**A**) HK-2 and (**B**) RPTEC/TERT1 after treatment with polymyxin derivatives.

The toxicity of the synthesized compounds was first assessed against two kidney cell lines using the cell viability assay (Fig. 4). HK-2 and RPTEC/TERT1 were specifically chosen for the study as these cell lines are responsive to polymyxin toxicity (30–33). Commercial PMB sulfate was used as a positive control for toxicity, whereas an inactive but non-nephrotoxic polymyxin derivative, F230, previously developed by Roberts et al. (20), served as a negative control for toxicity (Fig. 1).

Compounds **1**, **3**, and **4** were relatively nontoxic in HK-2 cells, with ~60%–90% viability at the highest concentration tested of 200 µM. However, compound **2** caused a substantial decline in cell viability resembling that of PMB. Analogous to the data with HK-2, compounds **1**, **3**, and **4** were relatively nontoxic in RPTEC/TERT1, with viabilities of ~60%–100% at the highest concentration tested. In contrast, compound **2** was more toxic than PMB in this cell line. Among the series, compound **3** consistently exhibited viabilities comparable to F230 in both HK-2 and RPTEC/TERT1. For the μg/mL equivalent of the compound concentrations used in the cell viability assays, as well as the 50% cytotoxicity concentrations (CC_50_), please see Tables S2 and S3 in the supplemental material.

### Antibacterial activity in wild-type Gram-negative bacteria

Since compounds **1** and **3** were the least toxic to the tested kidney cell lines, the antibacterial activity of these compounds was initially assessed using the broth microdilution assay (Table 1). Antibacterial activity was defined by the MIC—the lowest concentration of an agent that inhibits visible bacterial growth. In contrast with PMB, compound **1** was inactive against wild-type *P. aeruginosa* PAO1 (MIC of >128 µg/mL). Meanwhile, compound **1** displayed appreciable activity in wild-type *A. baumannii* ATCC 17978 (MIC of 16 µg/mL), and surprisingly retained activity against wild-type *E. coli* ATCC 25922 (MIC of 0.5–1 μg/mL). However, compound **3** was found to be inactive (MIC of >128 µg/mL) against all tested wild-type Gram-negative bacteria.

**TABLE 1:** Antibacterial activity of compounds **1** and **3** in wild-type and MDR Gram-negative bacteria

Strain	MIC (μg/mL)		
	Compound 1	Compound 3	PMB
*P. aeruginosa* PAO1	>128	>128	1
*A. baumannii* ATCC 17978	16	>128	2
*E. coli* ATCC 25922	0.5-1	>128	0.5

### Antibiotic potentiation in wild-type Gram-negative bacteria

Once the MICs have been determined, the capacity of compounds **1** and **3** to potentiate a panel of six antibiotics representative of various classes was then assessed against wild-type Gram-negative bacteria using the checkerboard assay (Tables 2 and 3). Due to the inherent activity of compound **1** in *E. coli* ATCC 25922, antibiotic potentiation was only tested in *P. aeruginosa* PAO1 and *A. baumannii* ATCC 17978.

**TABLE 2:** Antibiotic potentiation by 8 µg/mL (5 µM) of compound **1** or **3** in wild-type *P. aeruginosa* PAO1

Compound	Antibiotic	MIC_compound_ (μg/mL)	MIC_antibiotic_ (μg/mL)	FIC index	Interpretation	MIC_antibiotic_ + compound (μg/mL)	Potentiation
**1**	Rifampicin	>128	16	0.063 < x < 0.066	Synergy	0.063	≥256-fold
	Ceftazidime	>128	2	0.063 < x < 0.313	Synergy	0.5	4-fold
	Levofloxacin	>128	0.5	0.063 < x < 0.313	Synergy	0.125	4-fold
	Minocycline	>128	32	0.063 < x < 0.078	Synergy	0.5	64-fold
	Zoliflodacin	>128	>128	x < 0.125	Synergy	8	≥32-fold
	Pristinamycin	>128	256	0.063 < x < 0.064	Synergy	0.5	512-fold
**3**	Rifampicin	>128	32	0.063 < x < 1.063	Additive	32	1-fold
	Ceftazidime	>128	4	0.063 < x < 1.063	Additive	4	1-fold
	Levofloxacin	>128	1	0.063 < x < 1.063	Additive	1	1-fold
	Minocycline	>128	64	0.063 < x < 1.063	Additive	64	1-fold
	Zoliflodacin	>128	128	0.063 < x < 1.063	Additive	128	1-fold
	Pristinamycin	>128	256	0.063 < x < 1.063	Additive	256	1-fold

**TABLE 3:** Antibiotic potentiation by ¼ MIC (≤5 µM) of compound **1** or 8 µg/mL (5 µM) of compound **3** in wild-type *A. baumannii* ATCC 17978

Compound	Antibiotic	MIC_compound_ (μg/mL)	MIC_antibiotic_ (μg/mL)	FIC index	Interpretation	MIC_antibiotic_ + compound (μg/mL)	Potentiation
**1**	Rifampicin	16	8	0.252	Synergy	0.016	≥512-fold
	Ceftazidime	16	16	0.313	Synergy	1	16-fold
	Levofloxacin	32	0.125	0.650	Additive	0.063	2-fold
	Minocycline	32	1	0.254	Synergy	0.004	256-fold
	Zoliflodacin	16	4	0.258	Synergy	0.031	≥128-fold
	Pristinamycin	8	64	0.258	Synergy	0.5	128-fold
**3**	Rifampicin	>128	4	0.063 < x < 0.563	Additive	2	2-fold
	Ceftazidime	>128	4	0.063 < x < 1.063	Additive	4	1-fold
	Levofloxacin	>128	0.125	0.063 < x < 1.063	Additive	0.125	1-fold
	Minocycline	>128	0.125	0.063 < x < 1.063	Additive	0.125	1-fold
	Zoliflodacin	>128	2	0.063 < x < 1.063	Additive	2	1-fold
	Pristinamycin	>128	64	0.063 < x < 0.563	Additive	32	2-fold

The interaction between the compounds and the antibiotics was evaluated using the fractional inhibitory concentration (FIC) index. FIC indices of ≤0.5 is synergistic, 0.5 < × ≤ 4 is additive, whereas >4 is antagonistic (34). A working compound concentration of 8 µg/mL (5 µM) was generally used, as this concentration was considered nontoxic based on the cell viability assays (at least 40-fold lower than the CC_50_ values observed in both tested kidney cell lines). However, in cases where the compounds have an MIC ≤16 µg/mL, an even lower working concentration of ≤4 µg/mL (≤2 µM) was used for the synergy studies.

Against wild-type Gram-negative bacteria, compound **1** consistently synergized with all tested antibiotics, with the exception of levofloxacin in *A. baumannii* ATCC 17978. Compound **1** potentiated the antibiotics up to ≥512-fold and even reduced the MICs of rifampicin and pristinamycin below their interpretative susceptibility breakpoint (1 µg/mL) against both strains (35, 36). In combination with compound **1**, the susceptibility breakpoint of minocycline (4 µg/mL) was also reached against *P. aeruginosa* PAO1, as well as ceftazidime and zoliflodacin (8 µg/mL and 0.5 µg/mL, respectively) against *A. baumannii* ATCC 17978 (36, 37). In contrast, compound **3** only displayed additive interactions with the antibiotics.

### Antibacterial activity in clinical isolates of Gram-negative bacteria

Since compound **1** potentiated all tested antibiotics in wild-type Gram-negative bacteria, the standalone antibacterial activity was initially assessed in MDR and XDR clinical isolates (Table 4). MDR is defined as nonsusceptibility to at least one agent in ≥3 different antibiotic classes (38). XDR is defined as nonsusceptibility to at least one agent in all but ≤2 antibiotic classes (38). See Table S4 in the supplemental material for the antibiotic susceptibility profile of the tested strains.

**TABLE 4:** Antibacterial activity of compound **1** in clinical isolates of Gram-negative bacteria

Strain	MIC (μg/mL)		
	Compound 1	PMB	Colistin
*P. aeruginosa* 259-96916	>128	≤0.063	0.5
*P. aeruginosa* 262-101856	>128	0.5	2
*P. aeruginosa* 264-104354	>128	2	1
*P. aeruginosa* 101243^*a*^	>128	>128	1024
*P. aeruginosa* 114228^*a*^	>128	64	4
*A. baumannii* AB027	>128	0.25	0.25
*A. baumannii* AB031	32	≤0.125	0.25
*A. baumannii* 92247^*a*^	>128	8	4
*A. baumannii* 110193	16-32	1	0.5
*E. coli* 94393^*a*^	>128	4	4
*E. coli* 94474^*a*^	>128	16	16
*E. coli* 107115	1	0.25	0.125
*E. coli* 131629	0.5	≤0.25	≤0.25
*K. pneumoniae* 113250^*a*^	>128	128	256
*K. pneumoniae* 116381	>128	1	1
*E. cloacae* 117029	1	0.5	0.25
*E. cloacae* 121887^*a*^	>128	>128	>16

^*a*^ Colistin-resistant.

Similar to the observed antibacterial activity in the wild-type strains, compound **1** was inactive against MDR/XDR *P. aeruginosa* (MIC of >128 µg/mL) and moderately active against MDR but colistin-susceptible *A. baumannii* (MIC of 16–32 μg/mL), with the exception of AB027 (MIC of >128 µg/mL). For the Enterobacterales clinical isolates, although compound **1** was inactive against MDR *K. pneumoniae* (MIC of >128 µg/mL), compound **1** interestingly retained activity against MDR but colistin-susceptible *E. coli* 107115, *E. coli* 131629, and *E. cloacae* 117029 (MIC of 0.5–1 μg/mL).

### Antibiotic potentiation in clinical isolates of Gram-negative bacteria

The synergy of compound **1** with the panel of six antibiotics was further assessed against MDR and compound **1**-resistant clinical isolates (Tables 5 to 7). In MDR/XDR *P. aeruginosa*, potentiation of all tested antibiotics was retained in strains PA259-96916, PA262-101856, and PA264-105354 by up to ≥1,024-fold. However, antibiotic potentiation was significantly lower in colistin-resistant strains 101243 and 114228. Although decreased potentiation of ceftazidime, levofloxacin, and minocycline was observed in both MDR *A. baumannii* and Enterobacterales, the MICs of rifampicin, zoliflodacin, and pristinamycin were consistently reduced by compound **1** by more than ≥2,048-fold.

**TABLE 5:** Potentiation of antibiotics by 8 µg/mL (5 µM) of compound **1** in MDR/XDR *P. aeruginosa*

Strain	Antibiotic	MIC_compound 1_ (μg/mL)	MIC_antibiotic_ (μg/mL)	FIC index	Interpretation	MIC_antibiotic_ + compound (μg/mL)	Potentiation
*P. aeruginosa* 259-96916	Rifampicin	>128	16	0.063 < x < 0.064	Synergy	0.031	≥512-fold
	Ceftazidime	>128	512	0.063 < x < 0.125	Synergy	16	32-fold
	Levofloxacin	>128	512	0.063 < x < 0.094	Synergy	16	32-fold
	Minocycline	>128	64	0.063 < x < 0.070	Synergy	0.5	128-fold
	Zoliflodacin	>128	128	0.063 < x < 0.064	Synergy	0.125	≥1024-fold
	Pristinamycin	>128	256	0.063 < x < 0.064	Synergy	0.25	≥1024-fold
*P. aeruginosa* 262-101856	Rifampicin	>128	512	0.063 < x < 0.066	Synergy	2	256-fold
	Ceftazidime	>128	16	0.063 < x < 0.313	Synergy	4	4-fold
	Levofloxacin	>128	>64	x < 0.125	Synergy	4	≥32-fold
	Minocycline	>128	>128	x < 0.078	Synergy	2	≥128-fold
	Zoliflodacin	>128	>128	x < 0.078	Synergy	2	≥128-fold
	Pristinamycin	>128	>256	x < 0.078	Synergy	4	≥128-fold
*P. aeruginosa* 264-105354	Rifampicin	>128	32	0.063 < x < 0.066	Synergy	0.125	≥256-fold
	Ceftazidime	>128	128	0.063 < x < 0.188	Synergy	16	8-fold
	Levofloxacin	>128	64	0.063 < x < 0.125	Synergy	4	16-fold
	Minocycline	>128	>64	x < 0.070	Synergy	0.5	≥256-fold
	Zoliflodacin	>128	>128	x < 0.066	Synergy	0.5	≥512-fold
	Pristinamycin	>128	>256	x < 0.078	Synergy	4	≥128-fold
*P. aeruginosa* 101243^*a*^	Rifampicin	>128	16	0.063 < x < 0.188	Synergy	2	8-fold
	Ceftazidime	>128	512	0.063 < 0.563	Additive	256	2-fold
	Levofloxacin	>128	128	0.063 < x < 0.188	Synergy	16	8-fold
	Minocycline	>128	16	0.063 < x < 0.313	Synergy	4	4-fold
	Zoliflodacin	>128	>128	ND	ND	>128	ND
	Pristinamycin	>128	256	0.063 < x < 0.313	Synergy	64	4-fold
*P. aeruginosa* 114228^*a*^	Rifampicin	>128	16	0.063 < x < 0.563	Additive	8	2-fold
	Ceftazidime	>128	8	0.063 < x1.063	Additive	8	1-fold
	Levofloxacin	>128	2	0.063 < x < 0.0563	Additive	2	1-fold
	Minocycline	>128	>64	x < 0.313	Synergy	16	≥8-fold
	Zoliflodacin	>128	>128	ND	ND	>128	ND
	Pristinamycin	>128	256	0.063 < x < 0.031	Additive	256	1-fold

^*a*^ Colistin-resistant; ND, not determined.

**TABLE 6:** Potentiation of antibiotics by 4 µg/mL (2 µM) of compound **1** in MDR *A. baumannii*

Strain	Antibiotic	MIC_compound 1_ (μg/mL)	MIC_antibiotic_ (μg/mL)	FIC index	Interpretation	MIC_antibiotic_ + compound (μg/mL)	Potentiation
*A. baumannii* AB027	Rifampicin	>128	2	0.031 < x < 0.033	Synergy	0.004	≥512-fold
	Ceftazidime	>128	2048	0.031 < x < 0.531	Additive	1024	2-fold
	Levofloxacin	>128	8	0.031 < x < 0.531	Additive	4	2-fold
	Minocycline	>128	2	0.031 < x < 0.531	Additive	1	2-fold
	Zoliflodacin	>128	16	0.031 < x < 0.047	Synergy	0.25	64-fold
	Pristinamycin	>128	256	0.031 < x < 0.063	Synergy	8	32-fold
*A. baumannii* AB031	Rifampicin	>128	4	0.031 < x < 0.032	Synergy	0.004	≥1024-fold
	Ceftazidime	>128	>64	ND	ND	>64	ND
	Levofloxacin	>128	0.25	0.031 < x < 0.531	Additive	0.125	2-fold
	Minocycline	>128	2	0.031 < x < 0.531	Additive	1	2-fold
	Zoliflodacin	>128	128	0.031 < x < 0.063	Synergy	4	32-fold
	Pristinamycin	>128	256	0.031 < x < 0.039	Synergy	2	128-fold
*A. baumannii* 92247^*a*^	Rifampicin	>128	4	0.031 < x < 0.039	Synergy	0.031	128-fold
	Ceftazidime	>128	8	0.031 < x < 0.531	Additive	4	2-fold
	Levofloxacin	>128	0.063	0.031 < x < 1.031	Additive	0.063	1-fold
	Minocycline	>128	0.25	0.031 < x < 1.031	Additive	0.25	1-fold
	Zoliflodacin	>128	4	0.031 < x < 0.094	Synergy	0.25	≥16-fold
	Pristinamycin	>128	32	0.031 < x < 0.063	Synergy	1	32-fold
*A. baumannii* 110193	Rifampicin	32	4	0.126	Synergy	0.004	≥1024-fold
	Ceftazidime	32	256	0.375	Synergy	64	4-fold
	Levofloxacin	32	0.25	1.125	Additive	0.25	1-fold
	Minocycline	16	2	0.75	Additive	2	1-fold
	Zoliflodacin	32	256	0.156	Synergy	8	32-fold
	Pristinamycin	32	128	0.141	Synergy	2	64-fold

^*a*^ Colistin-resistant; ND, not determined.

**TABLE 7:** Potentiation of antibiotics by 8 µg/mL (5 µM) of compound **1** in MDR Enterobacterales

Strain	Antibiotic	MIC_compound 1_ (μg/mL)	MIC_antibiotic_ (μg/mL)	FIC index	Interpretation	MIC_antibiotic_ + compound (μg/mL)	Potentiation
*E. coli* 94393^*a*^	Rifampicin	>128	>16	x < 0.125	Synergy	0.5	≥32-fold
	Ceftazidime	>128	0.5	0.063 < x < 1.063	Additive	0.5	1-fold
	Levofloxacin	>128	>2	x < 0.563	Synergy	1	≥4-fold
	Minocycline	>128	0.5	0.063 < x < 0.313	Synergy	0.125	4-fold
	Zoliflodacin	>128	64	0.063 < x < 0.094	Synergy	2	32-fold
	Pristinamycin	>128	256	0.063 < x < 0.188	Synergy	16	16-fold
*E. coli* 94474^*a*^	Rifampicin	>128	8	0.063 < x < 0.125	Synergy	0.5	16-fold
	Ceftazidime	>128	0.5	0.063 < x < 0.531	Additive	0.25	2-fold
	Levofloxacin	>128	16	0.063 < x < 0.516	Additive	8	2-fold
	Minocycline	>128	64	0.063 < x < 0.188	Synergy	8	8-fold
	Zoliflodacin	>128	64	0.063 < x < 0.125	Synergy	4	16-fold
	Pristinamycin	>128	64	0.063 < x < 0.094	Synergy	2	32-fold
*K. pneumoniae* 113250^*a*^	Rifampicin	>128	32	0.063 < x < 0.125	Synergy	2	16-fold
	Ceftazidime	>128	0.5	0.063 < x < 1.063	Additive	0.5	1-fold
	Levofloxacin	>128	0.063	0.063 < x < 1.063	Additive	0.063	1-fold
	Minocycline	>128	>4	ND	ND	>4	ND
	Zoliflodacin	>128	128	0.063 < x < 0.094	Synergy	4	32-fold
	Pristinamycin	>128	256	0.063 < x < 0.125	Synergy	16	16-fold
*K. pneumoniae* 116381	Rifampicin	>128	>256	0.063	Synergy	0.25	≥2048-fold
	Ceftazidime	>128	8	0.063 < x < 0.078	Synergy	0.125	64
	Levofloxacin	>128	256	0.063 < x < 0.078	Synergy	4	64
	Minocycline	>128	128	0.063 < x < 0.093	Synergy	4	32
	Zoliflodacin	>128	256	0.063	Synergy	0.25	≥512-fold
	Pristinamycin	>128	256	0.063	Synergy	0.25	≥1024-fold
*E. cloacae* 121887^*a*^	Rifampicin	>128	>4	x < 0.313	Synergy	1	≥8-fold
	Ceftazidime	>128	0.25	0.063 < x < 1.063	Additive	0.25	1-fold
	Levofloxacin	>128	16	0.063 < x < 1.063	Additive	16	1-fold
	Minocycline	>128	0.5	0.063 < x < 1.063	Additive	0.5	1-fold
	Zoliflodacin	>128	32	0.063 < x < 0.188	Synergy	4	8-fold
	Pristinamycin	>128	256	0.063 < x < 0.563	Additive	128	2-fold

^*a*^ Colistin-resistant; ND, not determined.

Against all 14 clinical isolates, rifampicin was synergized in 13 (93%), zoliflodacin and pristinamycin in 12 (86%), minocycline in 8 (57%), and ceftazidime and levofloxacin in 5 (36%) of the strains. Notably, the MICs of all the tested antibiotics reached their susceptibility breakpoints in several strains. The best results were seen with rifampicin, which was rendered active in 12 of 16 (75%) rifampicin-resistant strains. Compound **1** at ≤8 µg/mL (≤5 µM) expanded the activity spectrum of zoliflodacin and pristinamycin, in which 6 of 16 (38%) zoliflodacin-resistant and 5 of 16 (31%) pristinamycin-resistant strains were sensitized to the antibiotics. The activity of minocycline and ceftazidime was also restored in 5 of 9 (56%) minocycline-resistant strains and 3 of 9 (33%) ceftazidime-resistant strains, respectively. Meanwhile, the least successful result was obtained with levofloxacin, in which only 1 of 9 (11%) levofloxacin-resistant strains was made susceptible. The susceptibility breakpoint of ceftazidime is 4 µg/mL in Enterobacterales and 8 µg/mL in *P. aeruginosa* and *Acinetobacter* spp., whereas the susceptibility breakpoint of levofloxacin is 0.5 µg/mL in Enterobacterales, 1 µg/mL in *P. aeruginosa*, and 2 µg/mL in *Acinetobacter* spp. (36).

### Time-kill kinetics in clinical isolates *P. aeruginosa* 259-96916 and *A. baumannii* AB027

**Fig 5:**
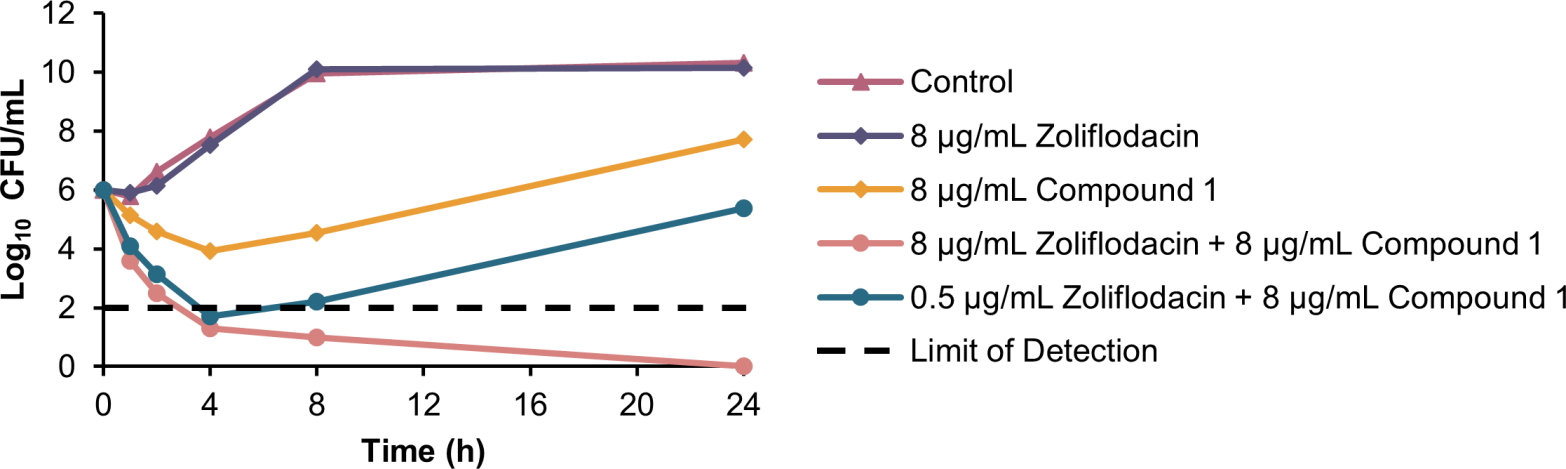
Time-kill kinetics of compound 1 and zoliflodacin against *P. aeruginosa* 259-96916.

**Fig 6:**
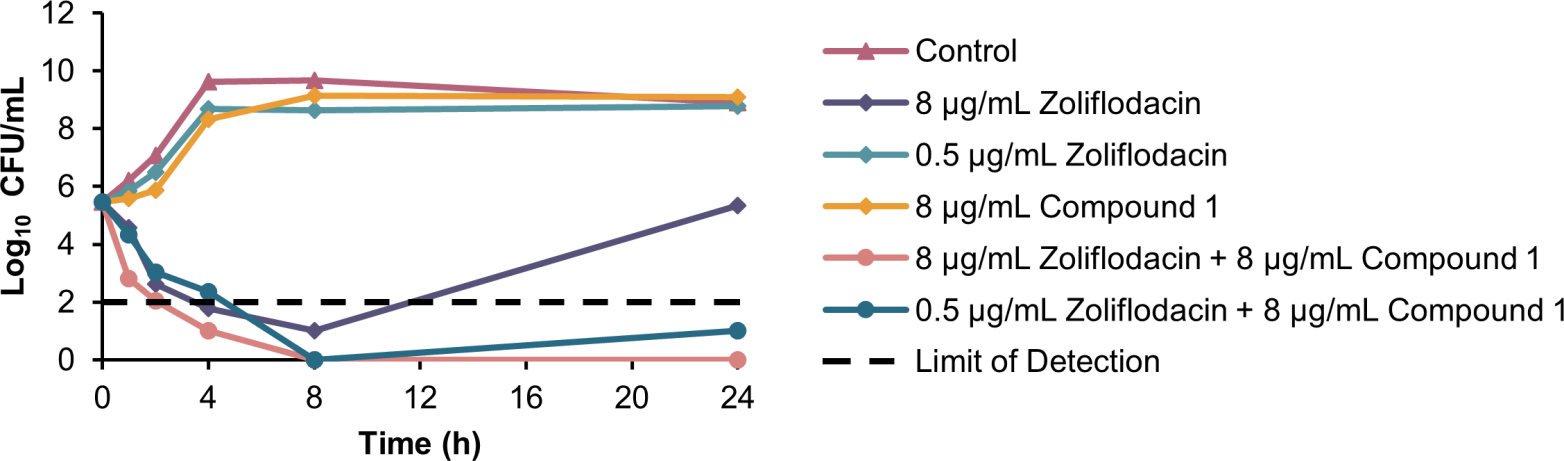
Time-kill kinetics of compound 1 and zoliflodacin against *A. baumannii* AB027.

To determine the rate of bacterial killing by the combination of compound **1** and zoliflodacin and to corroborate the synergistic data obtained with the checkerboard assays, the time-kill kinetics assay was performed (Fig. 5 and 6). *P. aeruginosa* 259-96916 and *A. baumannii* AB027 were chosen for the study to compare how bacterial killing would differ between two different organisms, and because the combination of compound **1** and zoliflodacin was most potent against these strains. Compound **1** was tested at a fixed concentration of 8 µg/mL (5 µM), whereas zoliflodacin was evaluated at either 8 µg/mL, representing a sub-MIC level, or 0.5 µg/mL, corresponding to the interpretative susceptibility breakpoint. Bactericidal and bacteriostatic activity correspond to a ≥ 3 log_10_ and a < 3 log_10_ decrease in bacterial load after 24 h relative to the starting inoculum, respectively (39). Synergistic activity corresponds to a ≥ 2 log_10_ decrease in bacterial load between the combination and the most active component after 24 h, as well as a ≥ 2 log_10_ decrease in bacterial load relative to the starting inoculum (40).

In *P. aeruginosa* 259-96916 (Fig. 5), zoliflodacin at 8 µg/mL resulted in a growth curve similar to that of untreated bacteria. On the other hand, compound **1** at 8 µg/mL (5 µM) resulted in a decrease in growth up to 4 h, with regrowth occurring at 8 h. Combining compound **1** with 0.5 µg/mL zoliflodacin resulted in a bacteriostatic effect, whereas complete sterilization was achieved after 24 h when zoliflodacin concentration was increased to 8 µg/mL.

In *A. baumannii* AB027 (Fig. 6), monotherapy of zoliflodacin at 0.5 µg/mL or compound **1** at 8 µg/mL (5 µM) resulted in growth curves similar to that of untreated bacteria. Although monotherapy of zoliflodacin at 8 µg/mL initially decreased bacterial loads, regrowth occurred after 8 h. The combination of compound **1** with either 0.5 or 8 µg/mL zoliflodacin appeared to eradicate the bacteria at 8 h. However, the combination consisting of 0.5 µg/mL zoliflodacin led to a slight regrowth after 24 h.

### Outer membrane permeabilization by compound 1

It is well established that polymyxins specifically bind to LPS of the outer membrane and that polymyxins have limited activity against Gram-positive bacteria due to the lack of LPS (6, 7). To confirm the mechanism of antibiotic potentiation, the ability of compound **1** to synergize with rifampicin in Gram-positive bacteria was assessed (Table 8). As expected, antibiotic potentiation was abolished in methicillin-resistant *S. aureus* ATCC 22592, *E. faecalis* ATCC 29212, and *E. faecium* ATCC 27270.

**TABLE 8:** Potentiation of rifampicin by 8 µg/mL (5 µM) compound **1** in Gram-positive bacteria

Strain	MIC_compound 1_ (μg/mL)	MIC_antibiotic_ (μg/mL)	FIC index	Interpretation	MIC_antibiotic_ + compound (μg/mL)	Potentiation
Methicillin-resistant *S. aureus* ATCC 22592	>128	512	0.063 < x < 1.063	Additive	512	1-fold
*E. faecalis* ATCC 29212	>128	0.5	0.063 < x < 1.063	Additive	0.5	1-fold
*E. faecium* ATCC 27270	>128	0.031	0.063 < x < 1.063	Additive	0.031	1-fold

It has also been established that polymyxins potentiate antibiotics by permeabilizing the outer membrane through the displacement of divalent cations that bridge and stabilize adjacent LPS molecules (6, 7). To verify the mechanism of action, the ability of compound **1** to potentiate zoliflodacin in elevated Mg^2+^ concentrations (25 mM) was assessed and compared with the standard Mg^2+^ concentrations (0.4–0.5 mM) in cation-adjusted Mueller Hinton broth (CAMHB) (Table 9). As predicted, the inclusion of competing Mg^2+^ caused a slight reduction in antibiotic potentiation (by 2-fold) in *A. baumannii* ATCC 17978, although a complete loss of antibiotic potentiation was seen in *P. aeruginosa* PAO1.

**TABLE 9:** Potentiation of zoliflodacin by 8 µg/mL (5 µM) compound **1** against *P. aeruginosa* PAO1 and 4 µg/mL compound **1** against *A. baumannii* ATCC 17978 in Mg^2+^-enriched media

Strain	[Mg^2+^] (mM)	MIC_compound 1_ (μg/mL)	MIC_antibiotic_ (μg/mL)	FIC index	Interpretation	MIC_antibiotic_ + compound (μg/mL)	Potentiation
*P. aeruginosa* PAO1	0.4–0.5	>128	>128	x < 0.125	Synergy	8	≥32-fold
	25	>128	1024	0.063 < x < 1.063	Additive	1024	1-fold
*A. baumannii* ATCC 17978	0.4–0.5	16	4	0.258	Synergy	0.031	≥128-fold
	25	>128	>32	x < 0.063	Synergy	4	≥64-fold

### Antibiotic potentiation in FBS-supplemented media

Strong binding of polymyxins to serum proteins reduces free concentration and thus affects the efficacy of polymyxins in the body. Therefore, the ability of compound **1** to potentiate zoliflodacin in increasing concentrations of fetal bovine serum (FBS) was assessed (Table 10). Antibiotic potentiation was lost when FBS concentrations reached 25% and 50% FBS in *P. aeruginosa* PAO1 and *A. baumannii* ATCC 17978, respectively.

**TABLE 10:** Potentiation of zoliflodacin by 8 µg/mL (5 µM) compound **1** against *P. aeruginosa* PAO1 and 4 µg/mL compound **1** against *A. baumannii* ATCC 17978 in FBS-supplemented media

Strain	%FBS	MIC_compound 1_ (μg/mL)	MIC_antibiotic_ (μg/mL)	FIC index	Interpretation	MIC_antibiotic_ + compound (μg/mL)	Potentiation
*P. aeruginosa* PAO1	0	>128	>128	x < 0.125	Synergy	8	≥32-fold
	10	>128	512	0.063 < x < 0.078	Synergy	8	64-fold
	25	>128	1024	0.063 < x < 1.063	Additive	1024	1-fold
	50	>128	1024	0.063 < x < 1.063	Additive	1024	1-fold
*A. baumannii* ATCC 17978	0	16	4	0.258	Synergy	0.031	≥128-fold
	10	>128	16	0.031 < x < 0.063	Synergy	0.5	32-fold
	25	>128	16	0.031 < x < 0.156	Synergy	2	8-fold
	50	>128	64	0.031 < x < 0.531	Additive	32	2-fold

### Cytotoxicity of polymyxin derivatives in neural cell lines

**Fig 7:**
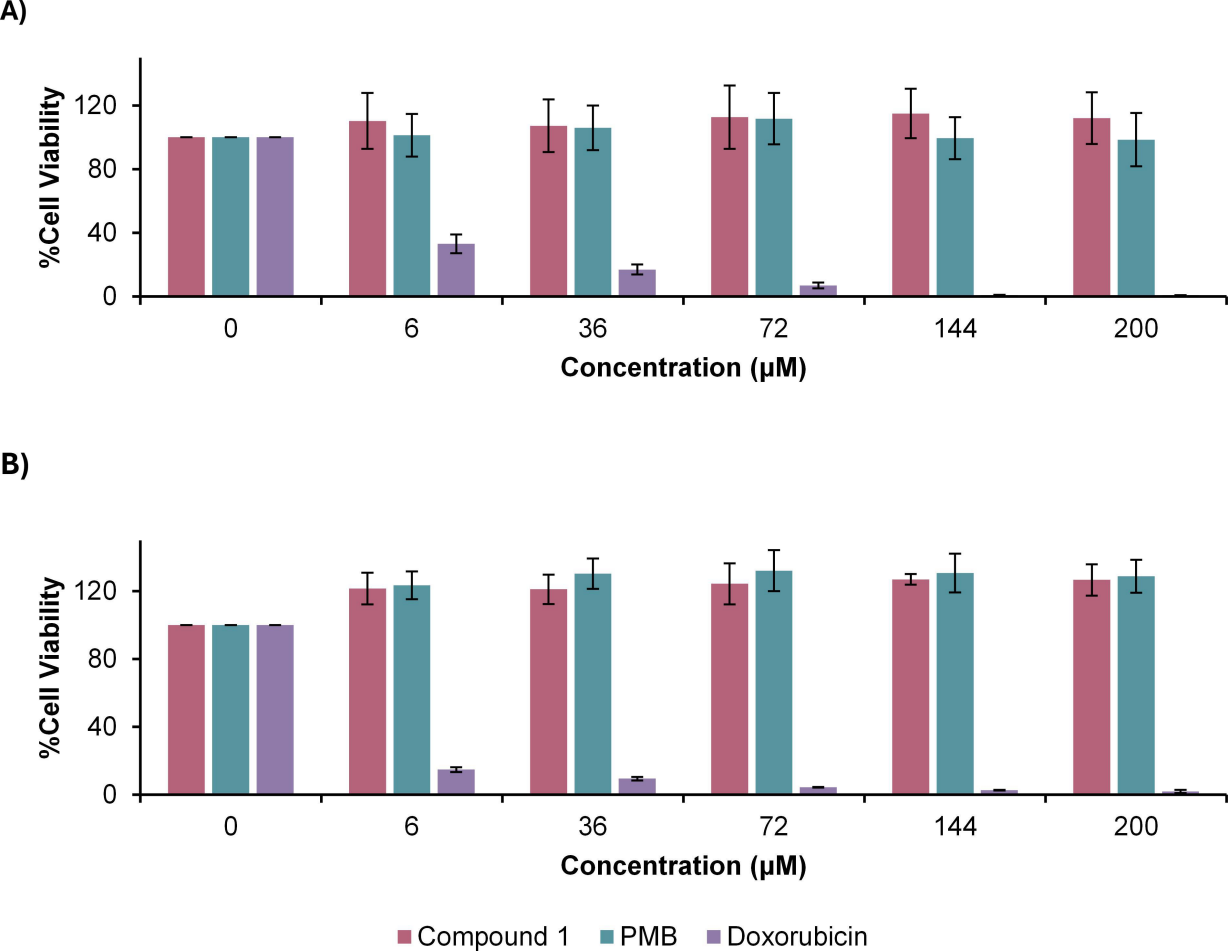
Viability of neural cell lines (**A**) U-87 MG and (**B**) SK-N-SH after treatment with compound 1.

Polymyxins possess moderate neurotoxic activity (6). Thus, the toxicity of lead compound **1** was also assessed against two neural cell lines, U-87 MG and SK-N-SH, using the cell viability assay (Fig. 7). Commercial PMB sulfate and anti-cancer agent doxorubicin were used as positive controls for toxicity. Although compound **1** showed no apparent toxicity to the neural cells—exhibiting cell viabilities exceeding 100% at the highest concentration tested—PMB also demonstrated a lack of toxicity.

## DISCUSSION

The overall hydrophobicity, positive charge, and basicity of polymyxins influence their toxicity and outer membrane permeabilizing capability (17–24, 27–29). We therefore focused on modifying these parameters in the design of PMB_3_-derived potentiators **1-4** (Fig. 1). As opposed to the major components of PMB that contain branched lipids, PMB_3_ has a linear lipid portion. Compound **1** was overall less hydrophobic with Dap residues in place of Dab. Converting the Dap amines of **1** to guanidines resulted in a more basic (higher pKa) derivative **2**. Substituting Dap^3^ in **1** or Dab^3^ in PMB_3_ with Asp yielded compounds **3** and **4** with fewer positive charges. Meanwhile, F230 is a previously reported inactive but non-nephrotoxic derivative by Roberts et al. that replaced both hydrophobic residues, D-Phe and Leu, with polar threonines (Thr) (20). Unlike PMB, which is produced via microbial fermentation, the compounds were produced via SPPS. This approach, coupled with the usage of expensive building blocks, may present significant challenges with regard to production cost and scalability.

Renal proximal tubular cells, including HK-2 and RPTEC/TERT1, predominantly express megalin, a membrane-associated transporter with a strong affinity for polymyxins. The binding of polymyxins to megalin facilitates endocytosis of polymyxins, subsequently activating the death receptor pathway of apoptosis (41, 42). Accordingly, HK-2 and RPTEC/TERT1 were employed to investigate polymyxin-induced cytotoxicity. As shown in Fig. 4, decreasing the hydrophobicity (compound **1**) or number of positive charges (compound **4**) greatly improved viability in contrast with PMB. When both properties were decreased (compound **3**), viability was further enhanced, similar to the F230 control. The increased toxicity from guanidinylation of compound **1** (compound **2**) can be attributed to an increase in basicity (higher pKa). This is consistent with previous findings (29).

Prior to testing the antibiotic potentiation of the nontoxic compounds **1** and **3**, the standalone activity was first determined in reference Gram-negative strains, such as *P. aeruginosa* PAO1, *A. baumannii* ATCC 17978, and *E. coli* ATCC 25922 (Table 1). The predominant loss of activity, with the exception of compound **1** against *E. coli* ATCC 25922, ascertains the importance of the Dab residues in maintaining antibacterial activity. Molecular modeling of polymyxins in complex with LPS revealed that Dab^1,5,7,8^ are directly involved in interacting with the phosphates in LPS molecules (43). Thus, slight modifications of these residues will inadvertently affect antibacterial activity. Shortening of the sidechain length of all Dab residues likely weakened interactions with the *P. aeruginosa* and *A. baumannii* LPS, whereas the modification was tolerated in *E. coli*. The diminished antibacterial activity of compound **1** relative to PMB is either due to minimizing basicity (Dap has a lower pKa than Dab) or due to steric factors (44). Substitution of Dab^3^ with Asp in compound **3** completely abolished antibacterial activity, suggesting that the introduction of a carboxyl group to the PMB scaffold likely attenuated interactions with LPS (45). These results agree with findings reported in a des-fatty acyl PMB derivative in which Dab^1^ was also replaced with another acidic residue, glutamic acid (17). The data also align with a prior study in which replacement of Thr^2^ with Asp led to a dramatic decrease in antibacterial activity (46). Structure-activity relationship studies have established that Thr^2,10^, along with Dab^3^, participate in hydrophilic contacts with the LPS Kdo moiety (43).

Six antibiotics belonging to different classes with diverse mechanisms of action were selected for the checkerboard assays to evaluate the capacity and spectrum of compound **1** as a potentiator. Rifampicin (ansamycin) inhibits bacterial RNA synthesis by binding to RNA polymerase (47). Ceftazidime (third-generation cephalosporin) interferes with peptidoglycan synthesis by binding to penicillin-binding proteins (1, 47). Levofloxacin (fluoroquinolone) inhibits bacterial DNA synthesis by binding to DNA gyrase or topoisomerase IV (1, 47). Minocycline (tetracycline) interferes with bacterial protein synthesis by binding to the 30S ribosomal subunit (1, 47). Zoliflodacin, a novel spiropyrimidinetrione being developed for the treatment of *Neisseria gonorrhoeae* infections and is in phase III clinical trials, interferes with bacterial DNA synthesis by binding to DNA gyrase at a site different from that of fluoroquinolines (48). Finally, pristinamycin (streptogramin) is a mixture composed of a cyclodepsipeptide (pristinamycin IA) and a polyunsaturated macrolactone (pristinamycin IIA) that interferes with bacterial protein synthesis by binding to the 50S ribosomal subunit (1, 49).

Compound **1** synergized with most of the tested antibiotics against *P. aeruginosa* PAO1 and *A. baumannii* ATCC 17978, highlighting its potential to expand the activity spectrum of rifampicin, zoliflodacin, and pristinamycin, as well as enhance the activity of ceftazidime, levofloxacin, and minocycline (Tables 2 and 3). Synergy is more apparent with antibiotics with limited outer membrane permeability possibly due to their hydrophobic character and/or high molecular weight (e.g., rifampicin, zoliflodacin, and pristinamycin), in comparison to antibiotics that are able to traverse the outer membrane through passive diffusion and/or the use of porins (e.g., ceftazidime, levofloxacin, and minocycline). The lack of synergistic interactions seen with compound **3** is presumed to be a result of possible electrostatic repulsions with LPS and the negatively charged Asp residue (45).

Advanced studies of compound **1** against clinical isolates were then performed (Table 4). Compound **1** remained inactive against MDR/XDR *P. aeruginosa*, and MDR *A. baumannii* and Enterobacterales, with the exception of colistin-susceptible *E. coli and E. cloacae*. The observed antibacterial activity for MDR/XDR *P*. aeruginosa, MDR *A. baumannii,* and *K. pneumoniae* is again likely due to reduced binding affinity to LPS as a consequence of either minimizing basicity (Dap has lower pKa than Dab) and/or affecting sterics (44). Meanwhile, inactivity against colistin-resistant strains is possibly due to modifications of the outer membrane that render it more positively charged, thereby causing electrostatic repulsion with polymyxins (6).

Synergy assessment was then carried out in the clinical isolates, excluding *E*. coli 107115, *E. coli* 131629, *and E*. cloacae 117029, which were susceptible to compound **1** (Tables 5 to 7). Similar to the data obtained in the wild-type strains, antibiotic potentiation was mostly retained, particularly for antibiotics with limited outer membrane permeability such as rifampicin, zoliflodacin, and pristinamycin. On the other hand, the activities of ceftazidime, levofloxacin, and minocycline, antibiotics that can overcome the permeability barrier through passive diffusion and/or porin uptake, were more challenging to enhance further. Ceftazidime, levofloxacin, and minocycline were especially more difficult to potentiate against the MDR *A. baumannii* and Enterobacterales clinical isolates, as most of the strains were already susceptible to these antibiotics.

In MDR/XDR *P. aeruginosa*, modest antibiotic potentiation and additive interactions were observed in colistin-resistant strains 101243 and 114228. A similar trend was observed in MDR *K. pneumoniae* 116381, wherein antibiotic potentiation was either not observed or was significantly lower in the colistin-resistant isolate 113250. Against MDR *A. baumannii*, rifampicin potentiation followed the same trend, whereas zoliflodacin and pristinamycin potentiation in the colistin-resistant strain 99247 was comparable with the MDR but colistin-susceptible *A. baumannii* strains. These results imply that LPS modifications, the main resistance determinant against polymyxins, also affect the ability of compound **1** to efficiently permeabilize the outer membrane. Nonetheless, compound **1** synergized with rifampicin in the MDR clinical isolates, with the majority of the strains becoming sensitized to rifampicin and achieving MICs below the interpretative susceptibility breakpoint. However, rifampicin potentiation is contemporarily utilized as a tool for assessing outer membrane permeabilizing capabilities. Therefore, it is perhaps more relevant to further explore the novel synergistic interaction of compound **1** with zoliflodacin.

The time-kill kinetics assay demonstrated the potency of the dual combination of compound **1** and zoliflodacin relative to zoliflodacin monotherapy (Fig. 5 and 6). In *P. aeruginosa*, wherein 8 µg/mL (5 µM) of compound **1** alone inhibited growth, the inclusion of zoliflodacin was necessary for eradication. A clear dose-dependent killing trend was also evident, with log_10_ reductions becoming more substantial as zoliflodacin concentration rose from 0.5 to 8 µg/mL. In *A. baumannii*, although 8 µg/mL of zoliflodacin monotherapy showed growth inhibition, the dual combination with compound **1** resulted in bacterial killing even at a lower concentration of zoliflodacin. The combination of compound **1** and zoliflodacin, both at a concentration of 8 µg/mL, resulted in ≥2 log_10_ decrease in bacterial load from the starting inoculum and from the most active monotherapy after 24 h. This indicates a synergistic interaction analogous to what was observed in the checkerboard studies. Of note, faster killing was exhibited against the *A. baumannii* strain, which was eradicated at 8 h, whereas the *P. aeruginosa* strain required 24 h for complete sterilization.

The presumed mechanism of outer membrane permeabilization by compound **1** was validated with its ability to synergize with antibiotics that have limited outer membrane permeability. Additional evidence supporting this is the suppression or complete loss of zoliflodacin potentiation upon inclusion of Mg^2+^ (Table 9). This suggests that Mg^2+^ competes with polymyxins for LPS binding and that this effect is more pronounced in *P. aeruginosa*. This difference possibly arises from the distinct outer membrane composition of each organism. The presence of excess Mg^2+^ likely significantly stabilizes the *P. aeruginosa* membrane, making it less vulnerable to polymyxin disruption. Moreover, compound **1** failed to synergize with rifampicin in Gram-positive strains, wherein the LPS is absent (Table 8). Collectively, these results imply that compound **1** enhances antibiotic activity by inducing outer membrane permeabilization through LPS interactions.

Although the amphiphilic nature of polymyxins is crucial to permeabilize the outer membrane, this property can also be detrimental as it increases binding affinity to serum proteins, thereby limiting bioavailability (50). Several studies have reported that PMB demonstrated a mean protein binding of >90% (51, 52), whereas another study has reported a median protein binding of 58% (53). To assess the impact of serum binding on antibiotic potentiation, FBS was added to the media to simulate these conditions (Table 10). Zoliflodacin potentiation by compound **1** was diminished with increasing concentrations of FBS. Although additive effects were observed at 50% FBS in both wild-type strains, zoliflodacin potentiation was retained at 25% FBS in *A. baumannii* ATCC 17978. These findings indicate that serum exposure influences the potency of the combination therapy, with a more substantial impact against *P. aeruginosa*. The disparity is likely attributable to outer membrane composition differences between the two organisms. *A. baumannii* is possibly more susceptible to polymyxin-induced permeability changes, permitting continued uptake of the partner antibiotic despite serum interference. However, there are limitations to this study, as the presence of serum may also have unknown effects on the viability and sensitivity of the bacteria.

In addition to the preliminary toxicity screening in HK-2 and RPTEC/TERT1, the toxicity of compound **1** was also assessed in neural cells (Fig. 7). However, the U-87 MG and SK-N-SH cell lines were not appropriate indicators of neurotoxicity and remained viable at the highest concentrations of both compound **1** and PMB. U-87 MG and SK-N-SH appear to be less responsive to polymyxins, potentially requiring higher concentrations to elicit cytotoxicity. This outcome may also be associated with minimal expression of membrane receptors that mediate polymyxin uptake.

Overall, lead compound **1**, in which all Dab residues were replaced with Dap, was found to be nontoxic to human kidney cell lines HK-2 and RPTEC/TERT1. Notably, the strain-specific antibacterial activity against colistin-susceptible *E. coli* and *E. cloacae* may offer a therapeutic advantage with regard to targeted interventions and minimizing unintended impact on commensal microbiota. Compound **1** consistently potentiated multiple antibiotics and lowered their MICs below clinical susceptibility breakpoints against a broad range of MDR Gram-negative bacteria. By displaying rapid bactericidal activity in combination with zoliflodacin against *P. aeruginosa* and *A. baumannii* clinical isolates, compound **1** shows promise as a candidate antibiotic potentiator to combat infections caused by top-priority pathogens.

## MATERIALS AND METHODS

### Chemistry

Solvents and reagents were purchased from Sigma-Aldrich (United States), Fisher Scientific (United States), and ChemImpex (United States). The linear PMB_3_ intermediates were produced via SPPS on a Thr-preloaded Wang resin using a fluorenylmethoxycarbonyl (Fmoc) deprotection strategy and by following a previously described protocol (54, 55). The *N*-terminus of the amino acids was protected with Fmoc. The side chain amine of Dab and Dap was protected with Boc or Cbz, and the side chain alcohol and carboxylic acid were protected with *tert*-butyl (tBu). Deprotection of Fmoc was performed using a solution of 4:1 DMF/piperidine (2 × 15 min). Coupling of the amino acids and the lipid was performed using a preactivated solution of TBTU and NMM in DMF, and gentle agitation with N_2_ gas (45 min – 1 h). After Fmoc deprotection and peptide or lipid coupling, the resin was washed with DMF (3×), DCM (3×), and DCM (3×) to remove piperidine from the reaction vessel. Completion of the reactions was monitored by performing the chloranil test (2% chloranil in DMF) on a small amount of resin. Once the desired peptide sequence has been assembled, the resin was washed with DCM (3×), MeOH (3×), and DCM (3×) to remove DMF from the reaction vessel. Cleavage of the peptide and deprotection of Boc and tBu was then performed using a solution of 95:5 TFA/H_2_O (1 h). Finally, the resin was washed with DCM (3×), and the filtrate was evaporated to afford the crude linear peptide.

Cyclization of the PMB_3_ intermediates was carried out using crude linear peptide, PyBOP (4.5 mol. equiv.), HOBt (6 mol. eq.), and NMM (8 mol. eq.) in anhydrous DMF (2 h). The cyclization reaction was performed under very dilute conditions to favor the intramolecular reaction between the Dab^4^ amine and the Thr^10^ carboxylic acid. Subsequently, the solvent was removed *in vacuo* and co-distillation with H_2_O (2×). The cyclized peptide was then precipitated using ice-cold H_2_O and filtered to afford a pale brown solid. The cyclized peptide was partially dissolved in H_2_O and stirred for at least 30 min to break the clumps. This resulting solution was then further filtered and washed with copious amounts of H_2_O to remove PyBOP-derived impurities, affording an off-white to white solid.

Finally, deprotection of the Cbz groups on the PMB_3_ derivatives was carried out via hydrogenolysis using 4:5:1 MeOH/AcOH/H_2_O, a catalytic amount of 20% Pd/C, and H_2_ gas. After an overnight reaction, the mixture was filtered using Celite, and the filtrate was evaporated to afford crude Cbz-deprotected peptide.

Compound **1** was guanidinylated using *N,N*-di-Boc-triflylguanidine, TEA, and 3:1 dioxane/H_2_O (4–5 days). The deprotection of the Boc groups was then performed using a solution of 1:1 TFA/DCM to afford compound **2** (2 h).

Compounds were purified by reverse-phase flash chromatography using SiliCycle (Canada) SiliaBond C_18_ (17%C, 40-63 µM, 60 Å) and a gradient of 0%–50% MeOH in H_2_O. The compounds were characterized by performing ^1^H, ^13^C, and 2D NMR experiments on a Bruker (Germany) AMX-400 or AMX-500 spectrometer. Compound masses were determined by conducting matrix-assisted laser desorption ionization time-of-flight mass spectrometry experiments on a Bruker (Germany) Ultraflextreme in either positive or negative ion mode using 2,5-dihydroxybenzoic acid or 2’,4’,6’-trihydroxyacetophenone monohydrate as the matrix, respectively.

### Mammalian cell lines and growth conditions

Kidney cell lines, HK-2 (CRL-2190) and RPTEC/TERT1 (CRL-4031), and neural cell lines, U-87 MG (HTB-14) and SK-N-SH (HTB-11), were obtained from American Type Culture Collection (ATCC). HK-2 cells were cultured in T75 flasks with keratinocyte serum-free medium supplemented with bovine pituitary extract, human epidermal growth factor, and 2.5% FBS. RPTEC/TERT1 cells were cultured in T75 flasks with Dulbecco’s modified Eagle’s medium:F12 supplemented with the ATCC (United States) human telomerase reverse transcriptase immortalized RPTEC growth kit, G418 (0.1 mg/mL final concentration), and 2% FBS. Both U-87 MG and SK-N-SH cells were cultured in minimal essential media supplemented with 10% FBS. The cells were incubated in a humidified atmosphere at 5% CO_2_ and at 37°C.

### Cell viability assay

The cytotoxicity of the compounds toward the mammalian cell lines was assessed using the cell viability assay protocols as previously described (29). Designated wells in 96-well plates were seeded with HK-2 (5,000 cells), RPTEC/TERT1 (8,000 cells), U-87 MG (7,000 cells), or SK-N-SH (8,000 cells) in 50 µL. Blank wells received an identical volume of only media. After incubating the cells for 24 h, all wells were treated with varying concentrations of the compound and incubated for 48 h. Subsequently, PrestoBlue reagent (Invitrogen, United States) was added to the wells to a final concentration of 10% (vol/vol), and the plate was incubated for an additional 1 h. Fluorescence was measured with a SpectraMax M2 plate reader (Molecular Devices, United States), at excitation and emission wavelengths of 560 and 590 nm, respectively. Values from the blank wells were subtracted from the corresponding wells containing cells. The cell viability relative to the controls with vehicle was calculated and presented as mean ± standard deviation of at least two experiments with five replicates each. CC_50_ values were obtained using GraphPad Prism (version 10.4.1) by non-linear regression (four-parameter logistic fit). For compounds where cell viability did not fall below 50% relative to the control within the tested concentration range, the CC_50_ represents a concentration estimated from the curve fit, accounting for the relative slope and rightward shift of each dose-response profile.

### Bacterial strains and growth conditions

The bacterial isolates were acquired from ATCC or the Canadian Ward Surveillance (CANWARD) study (56). CANWARD isolates were collected from infected patients admitted to participating medical centers across Canada. Gram-negative and Gram-positive bacterial isolates were grown in lysogeny broth (LB) and brain heart infusion, respectively, overnight in a shaking incubator at 250 rpm and at 37°C.

### Antibacterial susceptibility assay

The antibacterial activity of the antibiotics and compounds was assessed using the broth microdilution assay in accordance with Clinical Laboratory Standards Institute (CLSI) guidelines (36), and by following a previously described protocol (27–29, 54, 55). Bacterial culture grown overnight was diluted in saline to 0.5 McFarland turbidity and was further diluted 1:50 in CAMHB to attain a final concentration of 5 × 10^5^ colony-forming units (cfu)/mL for inoculation. The antibiotic or compound was serially diluted 2-fold in CAMHB on a 96-well plate and was incubated with equal volumes of bacterial inoculum overnight at 37°C. Wells containing only media were used as negative controls, and wells containing only bacteria were used as positive controls. Antibacterial activity was measured by the MIC, or the lowest concentration of agent required to inhibit visible bacterial growth. Turbidity was confirmed with a Molecular Devices (United States) EMax Plus microplate reader at a wavelength of 590 nm.

### Checkerboard assay

The ability of the compounds to potentiate antibiotics was assessed using the checkerboard assay in accordance with CLSI guidelines (36) and by following a previously described protocol (27–29, 54, 55). Bacterial culture grown overnight was diluted in saline to 0.5 McFarland turbidity and was further diluted 1:50 in CAMHB to attain a final concentration of 5 × 10^5^ cfu/mL for inoculation. The antibiotic and compounds were serially diluted 2-fold in CAMHB along the x- and y-axis, respectively, on a 96-well plate that was subsequently incubated with equal volumes of bacterial inoculum overnight at 37°C. Wells containing only media were used as negative controls, and wells containing only bacteria were used as positive controls. Synergy was measured by the FIC index, which is the summation of the individual FICs of the antibiotic and the compound. The FIC is calculated by dividing the MIC of one agent in the presence of another by the MIC of the agent alone. Turbidity was confirmed with a Molecular Devices (United States) EMax Plus microplate reader at a wavelength of 590 nm. The FBS studies were conducted by following a previously published protocol (29, 50). The FBS was inactivated by heating at 56°C for 30 min with constant swirling.

### Time-kill kinetics assay

The time- and concentration-dependent killing of the antibiotic/compound combinations was assessed using the time-kill kinetics assay by following a previously described protocol (27–29). Bacterial culture grown overnight was diluted in phosphate-buffered saline (PBS) to 0.5 McFarland turbidity and was further diluted 1:50 in LB. Bacterial cultures treated with varying concentrations of antibiotic alone, compound alone, or the combination of antibiotic and compound were incubated in a shaking incubator at 250 rpm and at 37°C. At designated time points, 100 µL aliquots were taken from the culture tubes, serially diluted in PBS, and plated on LB agar plates. The bacterial colonies were then counted after incubation of the plates for 18 h at 37°C.

## SUPPLEMENTAL MATERIAL

Data S1 aac.00712-25-s0001.csv SMILES, MICs, and CC50s of synthesized compounds.

Supplemental material aac.00712-25-s0002.docx Tables S1 to S4; Fig. S1 to S25.

ASM does not own the copyrights to Supplemental Material that may be linked to, or accessed through, an article. The authors have granted ASM a non-exclusive, world-wide license to publish the Supplemental Material files. Please contact the corresponding author directly for reuse.

## References

[1] Reygaert WC. 2018. An overview of the antimicrobial resistance mechanisms of bacteria. AIMS Microbiol 4:482–501. doi:10.3934/microbiol.2018.3.482 31294229 PMC6604941

[2] Laxminarayan R, Duse A, Wattal C, Zaidi AKM, Wertheim HFL, Sumpradit N, Vlieghe E, Hara GL, Gould IM, Goossens H, et al.. 2013. Antibiotic resistance-the need for global solutions. Lancet Infect Dis 13:1057–1098. doi:10.1016/S1473-3099(13)70318-9 24252483

[3] O’Neill J. 2014. Antimicrobial resistance: tackling a crisis for the health and wealth of nations. In The review on antimicrobial resistance

[4] Melander RJ, Melander C. 2017. The challenge of overcoming antibiotic resistance: an adjuvant approach? ACS Infect Dis 3:559–563. doi:10.1021/acsinfecdis.7b00071 28548487 PMC5798239

[5] Douafer H, Andrieu V, Phanstiel O 4th, Brunel JM. 2019. Antibiotic adjuvants: make antibiotics great again! J Med Chem 62:8665–8681. doi:10.1021/acs.jmedchem.8b01781 31063379

[6] Nang SC, Azad MAK, Velkov T, Zhou QT, Li J. 2021. Rescuing the last-line polymyxins: achievements and challenges. Pharmacol Rev 73:679–728. doi:10.1124/pharmrev.120.000020 33627412 PMC7911091

[7] Vaara M. 2018. New polymyxin derivatives that display improved efficacy in animal infection models as compared to polymyxin B and colistin. Med Res Rev 38:1661–1673. doi:10.1002/med.21494 29485690

[8] Vaara M. 1992. Agents that increase the permeability of the outer membrane. Microbiol Rev 56:395–411. doi:10.1128/mr.56.3.395-411.1992 1406489 PMC372877

[9] Vaara M, Siikanen O, Apajalahti J, Fox J, Frimodt-Møller N, He H, Poudyal A, Li J, Nation RL, Vaara T. 2010. A novel polymyxin derivative that lacks the fatty acid tail and carries only three positive charges has strong synergism with agents excluded by the intact outer membrane. Antimicrob Agents Chemother 54:3341–3346. doi:10.1128/AAC.01439-09 20479195 PMC2916294

[10] Eckburg PB, Lister T, Walpole S, Keutzer T, Utley L, Tomayko J, Kopp E, Farinola N, Coleman S. 2019. Safety, tolerability, pharmacokinetics, and drug interaction potential of SPR741, an intravenous potentiator, after single and multiple ascending doses and when combined with β-lactam antibiotics in healthy subjects. Antimicrob Agents Chemother 63:e00892-19. doi:10.1128/AAC.00892-19 31262767 PMC6709486

[11] Sun H-Y, Shields RK, Cacciarelli TV, Muder RR, Singh N. 2010. A novel combination regimen for the treatment of refractory bacteremia due to multidrug-resistant Pseudomonas aeruginosa in a liver transplant recipient. Transpl Infect Dis 12:555–560. doi:10.1111/j.1399-3062.2010.00543.x 20626709

[12] Ribera A, Benavent E, Lora-Tamayo J, Tubau F, Pedrero S, Cabo X, Ariza J, Murillo O. 2015. Osteoarticular infection caused by MDR Pseudomonas aeruginosa: the benefits of combination therapy with colistin plus β-lactams. J Antimicrob Chemother 70:3357–3365. doi:10.1093/jac/dkv281 26419763

[13] Li D, Rao H, Xu Y, Zhang M, Zhang J, Luo J. 2024. Monotherapy vs combination therapy in patients with Klebsiella pneumoniae bloodstream infection: a systematic review and meta-analysis. J Infect Chemother 30:372–378. doi:10.1016/j.jiac.2024.02.007 38369125

[14] Sayyahfar S, Choobdar FA, Mashayekhi M, Jazi FM. 2021. Successful management of pan-resistant Acinetobacter baumannii meningitis without intrathecal or intraventricular antibiotic therapy in a neonate. Infect Chemother 53:146–150. doi:10.3947/ic.2020.0202 32869561 PMC8032910

[15] Qiao L, Zuo W, Yang Y, Liu X, Wang Q, Yu J, Wu J, Xu T, Jiang J, Zhang B, Long Y. 2023. Clinical outcomes and safety of intravenous polymyxin B-based treatment in critically ill patients with carbapenem-resistant Acinetobacter baumannii nosocomial pneumonia. Int J Antimicrob Agents 62:106880. doi:10.1016/j.ijantimicag.2023.106880 37301311

[16] Xing H, Cheng C, Zhang Y, Cai Y, Wang X, Deng D, Xu L, Xu M, Chen J. 2021. Successful treatment with intrathecal and intravenous polymyxin B-based combination against MDR Acinetobacter baumannii meningitis in pediatric patient: a case report. Front Pediatr 9:564991. doi:10.3389/fped.2021.564991 34386463 PMC8353103

[17] Katsuma N, Sato Y, Ohki K, Okimura K, Ohnishi K, Sakura N. 2009. Development of des-fatty acyl-polymyxin B decapeptide analogs with Pseudomonas aeruginosa-specific antimicrobial activity. Chem Pharm Bull 57:332–336. doi:10.1248/cpb.57.332 19336926

[18] Sato Y, Shindo M, Sakura N, Uchida Y, Kato I. 2011. Novel des-fatty acyl-polymyxin B derivatives with Pseudomonas aeruginosa-specific antimicrobial activity. Chem Pharm Bull 59:597–602. doi:10.1248/cpb.59.597 21532197

[19] Brown P, Abbott E, Abdulle O, Boakes S, Coleman S, Divall N, Duperchy E, Moss S, Rivers D, Simonovic M, Singh J, Stanway S, Wilson A, Dawson MJ. 2019. Design of next generation polymyxins with lower toxicity: the discovery of SPR206. ACS Infect Dis 5:1645–1656. doi:10.1021/acsinfecdis.9b00217 31525992 PMC8152168

[20] Roberts KD, Zhu Y, Azad MAK, Han M-L, Wang J, Wang L, Yu HH, Horne AS, Pinson J-A, Rudd D, Voelcker NH, Patil NA, Zhao J, Jiang X, Lu J, Chen K, Lomovskaya O, Hecker SJ, Thompson PE, Nation RL, Dudley MN, Griffith DC, Velkov T, Li J. 2022. A synthetic lipopeptide targeting top-priority multidrug-resistant Gram-negative pathogens. Nat Commun 13:1625. doi:10.1038/s41467-022-29234-3 35338128 PMC8956739

[21] Magee TV, Brown MF, Starr JT, Ackley DC, Abramite JA, Aubrecht J, Butler A, Crandon JL, Dib-Hajj F, Flanagan ME, et al.. 2013. Discovery of Dap-3 polymyxin analogues for the treatment of multidrug-resistant Gram-negative nosocomial infections. J Med Chem 56:5079–5093. doi:10.1021/jm400416u 23735048

[22] Slingerland CJ, Lysenko V, Chaudhuri S, Wesseling CMJ, Barnes D, Masereeuw R, Martin NI. 2023. Semisynthetic polymyxins with potent antibacterial activity and reduced kidney cell toxicity. RSC Med Chem 14:2417–2425. doi:10.1039/d3md00456b 37974968 PMC10650952

[23] Chihara S, Tobita T, Yahata M, Ito A, Koyama Y. 1973. Enzymatic degradation of colistin isolation and identification of α-N-Acyl α, γ-diaminobutyric acid and colistin nonapeptide. Agric Biol Chem 37:2455–2463. doi:10.1271/bbb1961.37.2455

[24] Vaara M, Fox J, Loidl G, Siikanen O, Apajalahti J, Hansen F, Frimodt-Møller N, Nagai J, Takano M, Vaara T. 2008. Novel polymyxin derivatives carrying only three positive charges are effective antibacterial agents. Antimicrob Agents Chemother 52:3229–3236. doi:10.1128/AAC.00405-08 18591267 PMC2533495

[25] Kanazawa K, Sato Y, Ohki K, Okimura K, Uchida Y, Shindo M, Sakura N. 2009. Contribution of each amino acid residue in polymyxin B(3) to antimicrobial and lipopolysaccharide binding activity. Chem Pharm Bull (Tokyo) 57:240–244. doi:10.1248/cpb.57.240 19252313

[26] Moestrup SK, Cui S, Vorum H, Bregengård C, Bjørn SE, Norris K, Gliemann J, Christensen EI. 1995. Evidence that epithelial glycoprotein 330/megalin mediates uptake of polybasic drugs. J Clin Invest 96:1404–1413. doi:10.1172/JCI118176 7544804 PMC185763

[27] Ramirez DM, Ramirez D, Arthur G, Zhanel G, Schweizer F. 2022. Guanidinylated polymyxins as outer membrane permeabilizers capable of potentiating rifampicin, erythromycin, ceftazidime and aztreonam against gram-negative bacteria. Antibiotics (Basel) 11:1277. doi:10.3390/antibiotics11101277 36289935 PMC9598282

[28] Ramirez D, Berry L, Domalaon R, Brizuela M, Schweizer F. 2020. Dilipid ultrashort tetrabasic peptidomimetics potentiate novobiocin and rifampicin against multidrug-resistant gram-negative bacteria. ACS Infect Dis 6:1413–1426. doi:10.1021/acsinfecdis.0c00017 32357292

[29] Ramirez DM, Ramirez D, Dhiman S, Arora R, Lozeau C, Arthur G, Zhanel G, Schweizer F. 2023. Guanidinylated amphiphilic tobramycin derivatives synergize with β-lactam/β-lactamase inhibitor combinations against Pseudomonas aeruginosa. ACS Infect Dis 9:1754–1768. doi:10.1021/acsinfecdis.3c00217 37603592

[30] Vaara M, Vaara T. 2013. The novel polymyxin derivative NAB739 is remarkably less cytotoxic than polymyxin B and colistin to human kidney proximal tubular cells. Int J Antimicrob Agents 41:292–293. doi:10.1016/j.ijantimicag.2012.10.005 23182536

[31] Azad M, Finnin BA, Poudyal A, Davis K, Li J, Hill PA, Nation RL, Velkov T, Li J. 2013. Polymyxin B induces apoptosis in kidney proximal tubular cells. Antimicrob Agents Chemother 57:4329–4335. doi:10.1128/AAC.02587-12 23796937 PMC3754291

[32] Jarzina S, Di Fiore S, Ellinger B, Reiser P, Frank S, Glaser M, Wu J, Taverne FJ, Kramer NI, Mally A. 2022. Application of the adverse outcome pathway concept to in vitro nephrotoxicity assessment: kidney injury due to receptor-mediated endocytosis and lysosomal overload as a case study. Front Toxicol 4:864441. doi:10.3389/ftox.2022.864441 35516525 PMC9061999

[33] Payasi A, Yadav MK, Chaudhary S, Aggarwal A. 2024. Evaluating nephrotoxicity reduction in a novel polymyxin B formulation: insights from a 3D kidney-on-a-chip model. Antimicrob Agents Chemother 68:e0021924. doi:10.1128/aac.00219-24 39225483 PMC11459911

[34] Odds FC. 2003. Synergy, antagonism, and what the chequerboard puts between them. J Antimicrob Chemother 52:1–1. doi:10.1093/jac/dkg301 12805255

[35] Reissier S, Cattoir V. 2021. Streptogramins for the treatment of infections caused by Gram-positive pathogens. Expert Rev Anti Infect Ther 19:587–599. doi:10.1080/14787210.2021.1834851 33030387

[36] Clinical and Laboratory Standards Institute. 2025. Performance standards for antimicrobial susceptibility testing. 35th ed. Wayne, PA.

[37] Taylor SN, Marrazzo J, Batteiger BE, Hook EW, Seña AC, Long J, Wierzbicki MR, Kwak H, Johnson SM, Lawrence K, Mueller J. 2018. Single-dose Zoliflodacin (ETX0914) for treatment of urogenital gonorrhea. N Engl J Med 379:1835–1845. doi:10.1056/NEJMoa1706988 30403954

[38] Magiorakos A-P, Srinivasan A, Carey RB, Carmeli Y, Falagas ME, Giske CG, Harbarth S, Hindler JF, Kahlmeter G, Olsson-Liljequist B, Paterson DL, Rice LB, Stelling J, Struelens MJ, Vatopoulos A, Weber JT, Monnet DL. 2012. Multidrug-resistant, extensively drug-resistant and pandrug-resistant bacteria: an international expert proposal for interim standard definitions for acquired resistance. Clin Microbiol Infect 18:268–281. doi:10.1111/j.1469-0691.2011.03570.x 21793988

[39] Clinical and Laboratory Standards Institute. 1999. Methods for determining bactericidal activity of antimicrobial agents. Approved Guideline. Wayne, PA.

[40] Antimicrobial Agents and Chemotherapy. 2008. Antimicrobial agents and chemotherapy: 2008 instructions to authors. Antimicrob Agents Chemother 52:1–23. doi:10.1128/AAC.01407-07

[41] Li M, Balamuthusamy S, Simon EE, Batuman V. 2008. Silencing megalin and cubilin genes inhibits myeloma light chain endocytosis and ameliorates toxicity in human renal proximal tubule epithelial cells. Am J Physiol Renal Physiol 295:F82–90. doi:10.1152/ajprenal.00091.2008 18448595

[42] Wieser M, Stadler G, Jennings P, Streubel B, Pfaller W, Ambros P, Riedl C, Katinger H, Grillari J, Grillari-Voglauer R. 2008. hTERT alone immortalizes epithelial cells of renal proximal tubules without changing their functional characteristics. Am J Physiol Renal Physiol 295:F1365–F1375. doi:10.1152/ajprenal.90405.2008 18715936

[43] Pristovsek P, Kidric J. 1999. Solution structure of polymyxins B and E and effect of binding to lipopolysaccharide: an NMR and molecular modeling study. J Med Chem 42:4604–4613. doi:10.1021/jm991031b 10579822

[44] Clausell A, Garcia-Subirats M, Pujol M, Busquets MA, Rabanal F, Cajal Y. 2007. Gram-negative outer and inner membrane models: insertion of cyclic cationic lipopeptides. J Phys Chem B 111:551–563. doi:10.1021/jp064757+ 17228913

[45] Velkov T, Thompson PE, Nation RL, Li J. 2010. Structure--activity relationships of polymyxin antibiotics. J Med Chem 53:1898–1916. doi:10.1021/jm900999h 19874036 PMC2907661

[46] Li J, Guan D, Chen F, Shi W, Lan L, Huang W. 2021. Total and semisyntheses of polymyxin analogues with 2-Thr or 10-Thr modifications to decipher the structure–activity relationship and improve the antibacterial activity. J Med Chem 64:5746–5765. doi:10.1021/acs.jmedchem.0c02217 33909428

[47] Pancu DF, Scurtu A, Macasoi IG, Marti D, Mioc M, Soica C, Coricovac D, Horhat D, Poenaru M, Dehelean C. 2021. Antibiotics: conventional therapy and natural compounds with antibacterial activity-a pharmaco-toxicological screening. Antibiotics (Basel) 10:401. doi:10.3390/antibiotics10040401 33917092 PMC8067816

[48] Bradford PA, Miller AA, O’Donnell J, Mueller JP. 2020. Zoliflodacin: an oral spiropyrimidinetrione antibiotic for the treatment of Neisseria gonorrheae, including multi-drug-resistant isolates. ACS Infect Dis 6:1332–1345. doi:10.1021/acsinfecdis.0c00021 32329999

[49] Cooper EC, Curtis N, Cranswick N, Gwee A. 2014. Pristinamycin: old drug, new tricks? J Antimicrob Chemother 69:2319–2325. doi:10.1093/jac/dku167 24891428

[50] Logviniuk D, Fridman M. 2020. Serum prevents interactions between antimicrobial amphiphilic aminoglycosides and plasma membranes. ACS Infect Dis 6:3212–3223. doi:10.1021/acsinfecdis.0c00588 33174428

[51] Abodakpi H, Gohlke J, Chang K-T, Chow D-L, Tam VH. 2015. Analytical and functional determination of polymyxin B protein binding in serum. Antimicrob Agents Chemother 59:7121–7123. doi:10.1128/AAC.01815-15 26324262 PMC4604353

[52] Kwa ALH, Lim T-P, Low JGH, Hou J, Kurup A, Prince RA, Tam VH. 2008. Pharmacokinetics of polymyxin B1 in patients with multidrug-resistant Gram-negative bacterial infections. Diagn Microbiol Infect Dis 60:163–167. doi:10.1016/j.diagmicrobio.2007.08.008 17916420

[53] Sandri AM, Landersdorfer CB, Jacob J, Boniatti MM, Dalarosa MG, Falci DR, Behle TF, Bordinhão RC, Wang J, Forrest A, Nation RL, Li J, Zavascki AP. 2013. Population pharmacokinetics of intravenous polymyxin B in critically ill patients: implications for selection of dosage regimens. Clin Infect Dis 57:524–531. doi:10.1093/cid/cit334 23697744

[54] Domalaon R, Yang X, Lyu Y, Zhanel GG, Schweizer F. 2017. Polymyxin B_3_-tobramycin hybrids with Pseudomonas aeruginosa-selective antibacterial activity and strong potentiation of rifampicin, minocycline, and vancomycin. ACS Infect Dis 3:941–954. doi:10.1021/acsinfecdis.7b00145 29045123

[55] Domalaon R, Berry L, Tays Q, Zhanel GG, Schweizer F. 2018. Development of dilipid polymyxins: investigation on the effect of hydrophobicity through its fatty acyl component. Bioorg Chem 80:639–648. doi:10.1016/j.bioorg.2018.07.018 30053708

[56] Zhanel GG, Adam HJ, Baxter MR, Fuller J, Nichol KA, Denisuik AJ, Golden AR, Hink R, Lagacé-Wiens PRS, Walkty A, Mulvey MR, Schweizer F, Bay D, Hoban DJ, Karlowsky JA, Canadian Antimicrobial Resistance Alliance (CARA) and CANWARD. 2019. 42936 pathogens from Canadian hospitals: 10 years of results (2007-16) from the CANWARD surveillance study. J Antimicrob Chemother 74:iv5–iv21. doi:10.1093/jac/dkz283 31505641

