# Supplementary material for: Noncytotoxic polymyxin derivatives enhance antibiotic action against multidrug-resistant Gram-negative bacteria
Source: Antimicrob Agents Chemother. 2025 Sep 22;69(11):e00712-25. doi: 10.1128/aac.00712-25 (PMC12587545; doi:10.1128/aac.00712-25)
Supplement: Supplemental material — Tables S1 to S4; Fig. S1 to S25. [file aac.00712-25-s0002.docx]

**Supplemental Material**

**Noncytotoxic Polymyxin Derivatives Enhance Antibiotic Action against Multidrug-Resistant Gram-Negative Bacteria**

Danyel Ramirez,^1^ Danzel Marie Ramirez,^1^ Rajat Arora,^1^ Gilbert Arthur,^2^ Frank Schweizer^1,3#^

^1^Department of Chemistry, University of Manitoba, Winnipeg, Manitoba, Canada

^2^Department of Biochemistry and Medical Genetics, Winnipeg, Manitoba, Canada

^3^Department of Medical Microbiology and Infectious Diseases, Winnipeg, Manitoba, Canada

**Running Head:** Nontoxic Polymyxin-Based Adjuvant for Gram-Negatives

#Address correspondence to Frank Schweizer, frank.schweizer@umanitoba.ca

**Table of Contents**

1. Molecular Weight of Compounds in Salt Form (Table S1) .………………………………………….S2
2. Compound Concentrations Used in the Cell Viability Assays (Table S2) …..…………..……..S2
3. CC_50_ and Therapeutic Indices of Compounds (Table S3) ………………..…………………..……..S2
4. Chemical Characterization of Compounds …..………………..…………………………………...….S5
5. NMR Spectra of Compounds (Figures S1-25) ..…………………..……………..….……………..…..S6
6. Resistance Phenotype of MDR Clinical Isolates (Table S4) .……………………………………….S19

**1. Molecular Weight of Compounds in Salt Form**

**Table S1.** Molecular weight of compounds as a base and as a TFA salt.

| **Compound** | **Molecular Weight (base) (g/mol)** | **Molecular Weight (TFA salt) (g/mol)** |
| --- | --- | --- |
| F230 | 1131.3450 | 1701.4615 (x 5 TFA) |
| 1 | 1119.3370 | 1689.4535 (x 5 TFA) |
| 2 | 1330.5500 | 1900.6665 (x 5 TFA) |
| 3 | 1148.3310 | 1604.4242 (x 4 TFA) |
| 4 | 1204.4390 | 1660.5322 (x 4 TFA) |

**2. Compound Concentrations Used in the Cell Viability Assays**

**Table S2.** Compound concentrations used in the cell viability assays.

| **Compound** |  | **Concentration (μM)** | | | | |
| --- | --- | --- | --- | --- | --- | --- |
|  |  | **6** | **36** | **72** | **144** | **200** |
| F230 | **Concentration**  **(μg/mL)** | 3.5 | 21.2 | 42.3 | 84.6 | 117.5 |
| 1 |  | 3.6 | 21.3 | 42.6 | 85.2 | 118.4 |
| 2 |  | 3.2 | 18.9 | 37.9 | 75.8 | 105.2 |
| 3 |  | 3.7 | 22.4 | 44.9 | 89.8 | 124.7 |
| 4 |  | 3.6 | 21.7 | 43.4 | 86.7 | 120.4 |

**3.** **CC_50_ and Therapeutic Indices of Compounds**

**Table S3.** CC_50_ and therapeutic indices (TI) of compounds.

| **Compound** | **CC_50_ in HK-2 (μM)** | **TI_1_*** | **CC_50_ in RPTEC/TERT1 (μM)** | **TI_2_*** |
| --- | --- | --- | --- | --- |
| F230 | 263.1 | 56.0 | 471.4 | 100.3 |
| 1 | 238.7 | 50.4 | ND | ND |
| 2 | 26.9 | 6.4 | 28.0 | 6.7 |
| 3 | 215.5 | 43.2 | 627.2 | 125.8 |
| 4 | 236.8 | 49.2 | 238.8 | 49.6 |

*Therapeutic Index (TI) = CC_50_ / working concentration of 8 μg/mL (4.2 – 5 μM)

ND: not determined

**4. Chemical Characterization of Compounds**

**F230**

^1^H NMR (400 MHz, D_2_O) δ 4.59 – 4.55 (m, 1H), 4.45 – 4.10 (m, 13H) (Dab_1,3,4,5,8,9α_ + Thr_2,6,7,10α_ + Thr_2,6,7,10β_), 3.34 – 3.25 (m, 1H), 3.16 – 2.95 (m, 11H) (Dab_1,3,4,5,8,9β_), 2.29 – 2.07 (m, 9H), 2.06 – 1.98 (m, 3H), 1.94 – 1.77 (m, 2H) (CH_2_ lipid + Dab_1,3,4,5,8,9γ_), 1.57 – 1.47 (m, 2H, CH_2_ lipid), 1.26 – 1.08 (m, 20H, CH_2_ lipid + Thr_2,6,7,10γ_), 0.81 – 0.72 (m, 3H, CH_3_ lipid). ^13^C NMR (126 MHz, D_2_O) δ 178.0, 173.2, 173.1, 173.0, 172.6, 172.5, 172.4, 171.8, 171.6, 67.1, 67.0, 66.7, 66.4, 59.8, 59.8, 59.0, 58.6, 52.5, 52.0, 51.9, 51.2, 51.2, 50.8, 36.6, 36.4, 36.3, 36.2, 35.5, 31.0, 30.7, 29.8, 28.7, 28.5, 28.3, 28.1, 27.9, 25.3, 22.0, 19.3, 19.2, 19.0, 18.8, 13.4. MS (+TOF) m/z calculated for C_48_H_90_N_16_O_15_ [M+Na]^+^: 1153.6670, found: 1153.695.

**Compound 1**

^1^H NMR (400 MHz, D_2_O) δ 7.27 – 7.14 (m, 3H, Phe_6γ_), 7.13 – 7.03 (m, 2H, Phe_6γ_), 4.44 – 4.27 (m, 3H), 4.24 – 4.00 (m, 5H) (Dap_α_ + Thr_2,10α_ + Phe_6α_ + Leu_7α_ + Thr_2,10β_ ), 3.55 – 3.44 (m, 2H), 3.40 – 3.32 (m, 2H), 3.29 – 2.77 (m, 10H) (Dab_4β_ + Dap_1,3,5,8,9β_ + Phe_6β_), 2.20 (t, *J* = 7.5 Hz, 2H, CH_2_ lipid), 1.90 – 1.71 (m, 2H, Dab_4γ_), 1.50 – 1.27 (m, 4H, CH_2_ lipid + Leu_7β_), 1.21 – 0.99 (m, 15H, CH_2_ lipid + Thr_2,10γ_ + Leu_7γ_), 0.75 – 0.50 (m, 9H, CH_3_ lipid + Leu_7δ_). ^13^C NMR (126 MHz, D_2_O) δ 178.1, 176.1, 173.3, 173.2, 172.2, 171.1, 170.9, 169.9, 169.6, 136.0, 129.0, 128.9, 127.4, 67.2, 67.0, 59.5, 58.9, 55.1, 52.2, 51.8, 51.7, 51.4, 50.9, 50.7, 50.5, 40.7, 39.8, 39.7, 39.3, 39.1, 36.8, 35.6, 35.3, 31.0, 28.4, 28.1, 25.1, 24.0, 22.3, 22.0, 20.5, 19.0, 18.7, 13.4. MS (+TOF) m/z calculated for C_50_H_86_N_16_O_13_ [M+Na]^+^: 1141.6458, found: 1141.5802; [M+K]^+^: 1157.7543, found: 1157.5413.

**Compound 2**

^1^H NMR (500 MHz, D_2_O) δ 7.45 – 7.33 (m, 3H, Phe_6γ_), 7.30 – 7.24 (m, 2H, Phe_6γ_), 4.74 – 4.68 (m, 2H), 4.67 – 4.63 (m, 1H), 4.60 – 4.54 (m, 1H), 4.54 – 4.39 (m, 3H), 4.38 – 4.33 (m, 1H), 4.32 – 4.19 (m, 3H), (Thr_2,10β_ + Dap_1,3,5,8,9α_ + Thr_2,10α_ + Phe_6α_ + Leu_7α_), 3.84 – 3.50 (m, 9H), 3.47 – 3.34 (m, 2H), 3.21 – 3.03 (m, 3H) (Dab_4β_ + Dap_1,3,5,8,9β_ + Phe_6β_), 2.35 (t, *J* = 6.6 Hz, 2H, CH_2_ lipid), 2.05 – 1.89 (m, 2H Dab_4γ_), 1.66 – 1.57 (m, 2H, CH_2_ lipid), 1.50 – 1.16 (m, 16H, CH_2_ lipid + Thr_2,10γ_ + Leu_7β_), 1.00 – 0.60 (m, 10H, CH_3_ lipid + Leu_7δ_ + Leu_7γ_). ^13^C NMR (126 MHz, D_2_O) δ 178.0, 175.2, 173.5, 173.0, 171.9, 171.6, 171.5, 171.0, 170.8, 170.2, 157.2, 157.1, 135.4, 129.1, 129.1, 127.6, 67.1, 66.4, 59.9, 59.0, 56.1, 53.7, 52.9, 52.5, 52.2, 52.1, 51.4, 41.8, 41.7, 41.5, 40.9, 39.2, 36.9, 35.6, 31.0, 28.3, 28.2, 25.3, 23.6, 22.3, 22.0, 20.5, 19.1, 18.7, 13.4. MS (+TOF) m/z calculated for C_55_H_97_N_26_O_13_ [M+H]^+^: 1330.7801, found: 1330.7923; [M+Na]^+^: 1352.7626, found: 1352.7707.

**Compound 3**

^1^H NMR (400 MHz, D_2_O) δ 7.27 – 7.02 (m, 5H, Phe_6γ_), 4.59 – 4.53 (m, 2H), 4.49 – 4.33 (m, 2H) (Dap_α_ + Thr_2,10α_ + Phe_6α_), 4.23 – 4.04 (m, 5H, Thr_2,10β_ + Leu_7α_ + Asp_3α_), 3.59 – 3.33 (m, 4H), 3.23 – 2.92 (m, 8H), 2.86 – 2.69 (m, 2H) (Dab_4β_ + Dap_1,5,8,9β_ + Phe_6β_ + Asp_3β_), 2.19 (t, *J* = 7.5 Hz, 2H, CH_2_ lipid), 1.96 – 1.66 (m, 2H, Dab_4γ_), 1.52 – 1.25 (m, 4H, CH_2_ lipid + Leu_7β_), 1.21 – 0.91 (m, 15H, CH_2_ lipid + Thr_2,10γ_ + Leu_7γ_), 0.77 – 0.52 (m, 9H, CH_3_ lipid + Leu_7δ_). ^13^C NMR (126 MHz, D_2_O) δ 178.0, 176.1, 174.1, 173.4, 173.1, 172.3, 171.5, 171.2, 171.0, 170.7, 169.6, 135.9, 129.0, 128.9, 127.4, 67.0, 66.8, 59.7, 59.0, 55.2, 52.2, 51.5, 51.3, 50.7, 50.6, 50.4, 50.2, 49.2, 46.7, 40.7, 39.8, 39.8, 39.3, 39.1, 36.7, 35.6, 35.1, 31.0, 28.4, 28.1, 25.1, 24.0, 22.3, 22.0, 20.5, 19.0, 18.8, 13.4, 8.3.MS (+TOF) m/z calculated for C_51_H_85_N_15_O_15_ [M-H]^-^: 1146.6277, found: 1146.6970.

**Compound 4**

^1^H NMR (400 MHz, D_2_O) δ 7.33 – 7.20 (m, 3H, Phe_6γ_), 7.19 – 7.12 (m, 2H, Phe_6γ_), 4.49 – 4.35 (m, 3H), 4.29 – 4.06 (m, 6H) (Dab_α_ + Thr_2,10α_ + Thr_2,10β_ + Asp_3α_ + Phe_6α_ + Leu_7α_), 3.19 – 3.12 (m, 2H), 3.09 – 2.93 (m, 8H), 2.91 – 2.68 (m, 4H) (Dab_1,4,5,8,9β_ + Asp_3β_ + Phe_6β_), 2.32 – 1.62 (m, 14H, CH_2_ lipid + Dab_1,4,5,8,9γ_ + Leu_7β_), 1.58 – 1.44 (m, 2H, CH_2_ lipid), 1.40 – 1.03 (m, 15H, CH_2_ lipid + Thr_2,10γ_ + Leu_7γ_), 0.78 – 0.48 (m, 9H, CH_3_ lipid + Leu_7δ_). ^13^C NMR (126 MHz, D_2_O) δ 177.9, 177.9, 175.0, 174.1, 173.5, 173.0, 172.3, 171.5, 171.4, 129.1, 127.7, 127.5, 114.5, 67.0, 66.3, 59.8, 58.9, 56.3, 56.0, 52.5, 51.9, 51.8, 51.3, 51.2, 50.6, 50.5, 50.1, 45.5, 39.2, 39.1, 36.6, 36.5, 36.4, 35.5, 35.4, 31.0, 29.7, 28.5, 28.4, 28.3, 28.1, 25.3, 23.7, 22.4, 22.0, 20.4, 19.1, 18.9, 13.4.MS (+TOF) m/z calculated for C_55_H_93_N_15_O_15_ [M-H]^-^: 1202.6903, found: 1202.7627.

**5. NMR Spectra of Compounds**


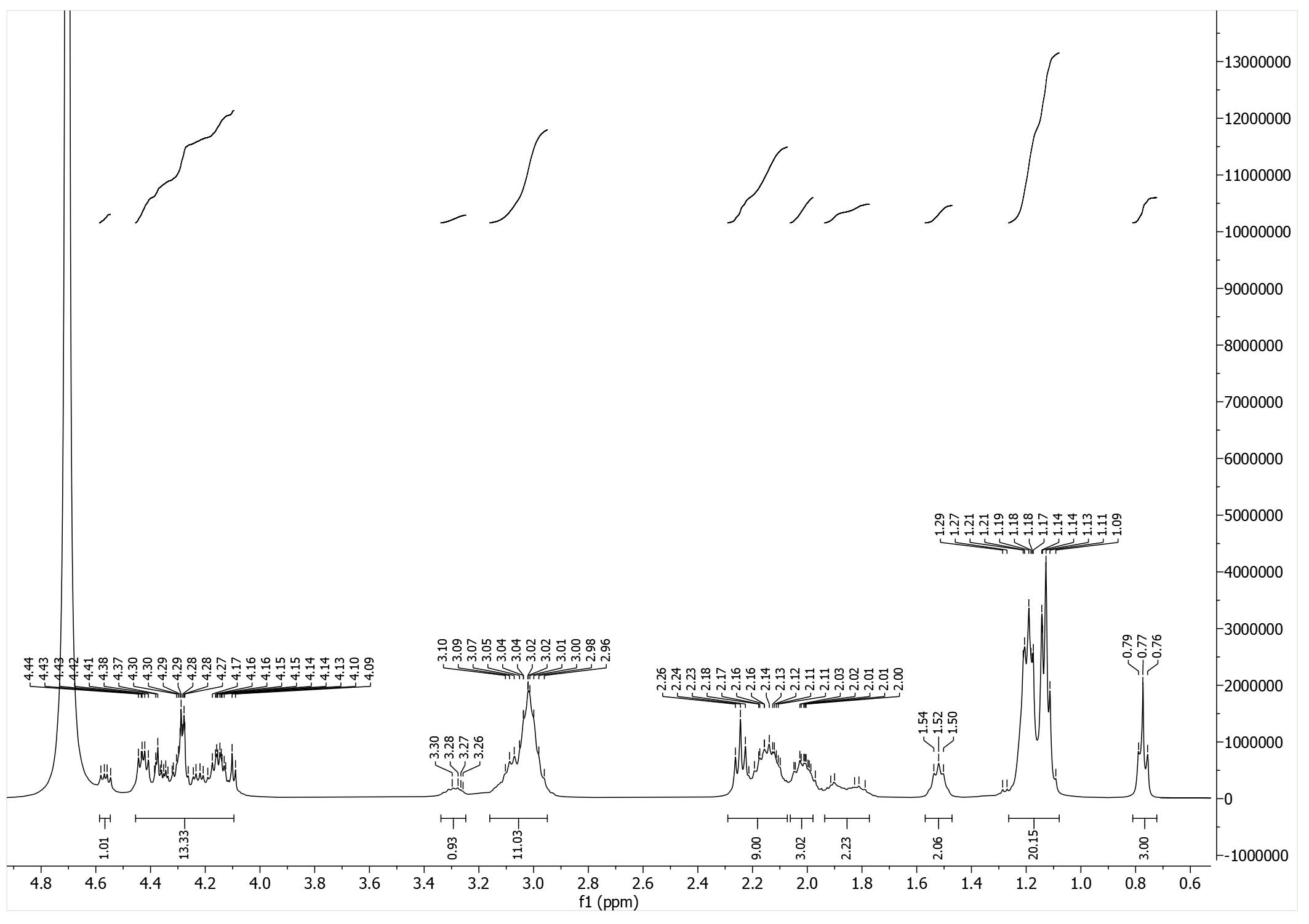


**Figure S1.** ^1^H NMR of F230.


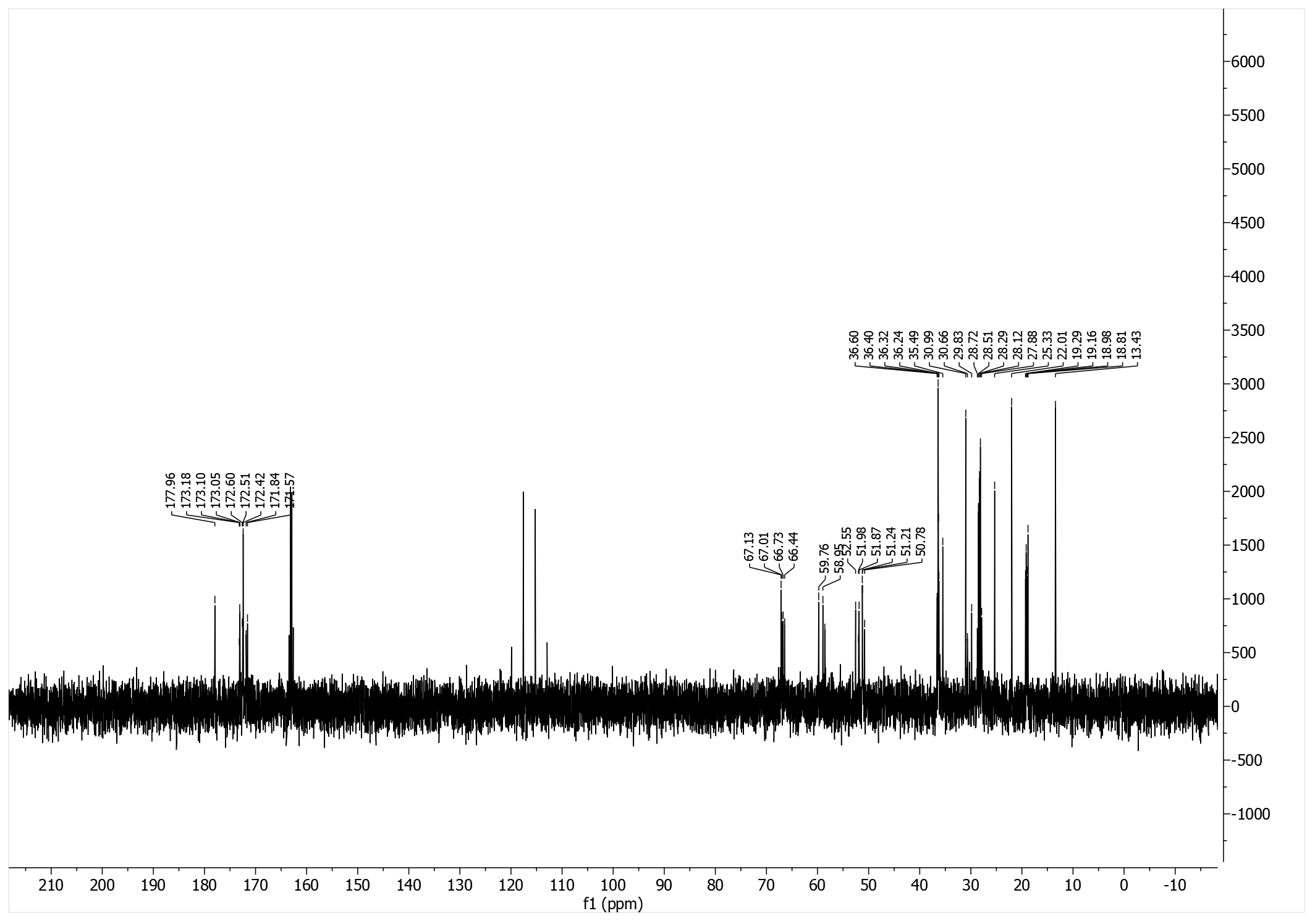


**Figure S2.** ^13^C NMR of F230.


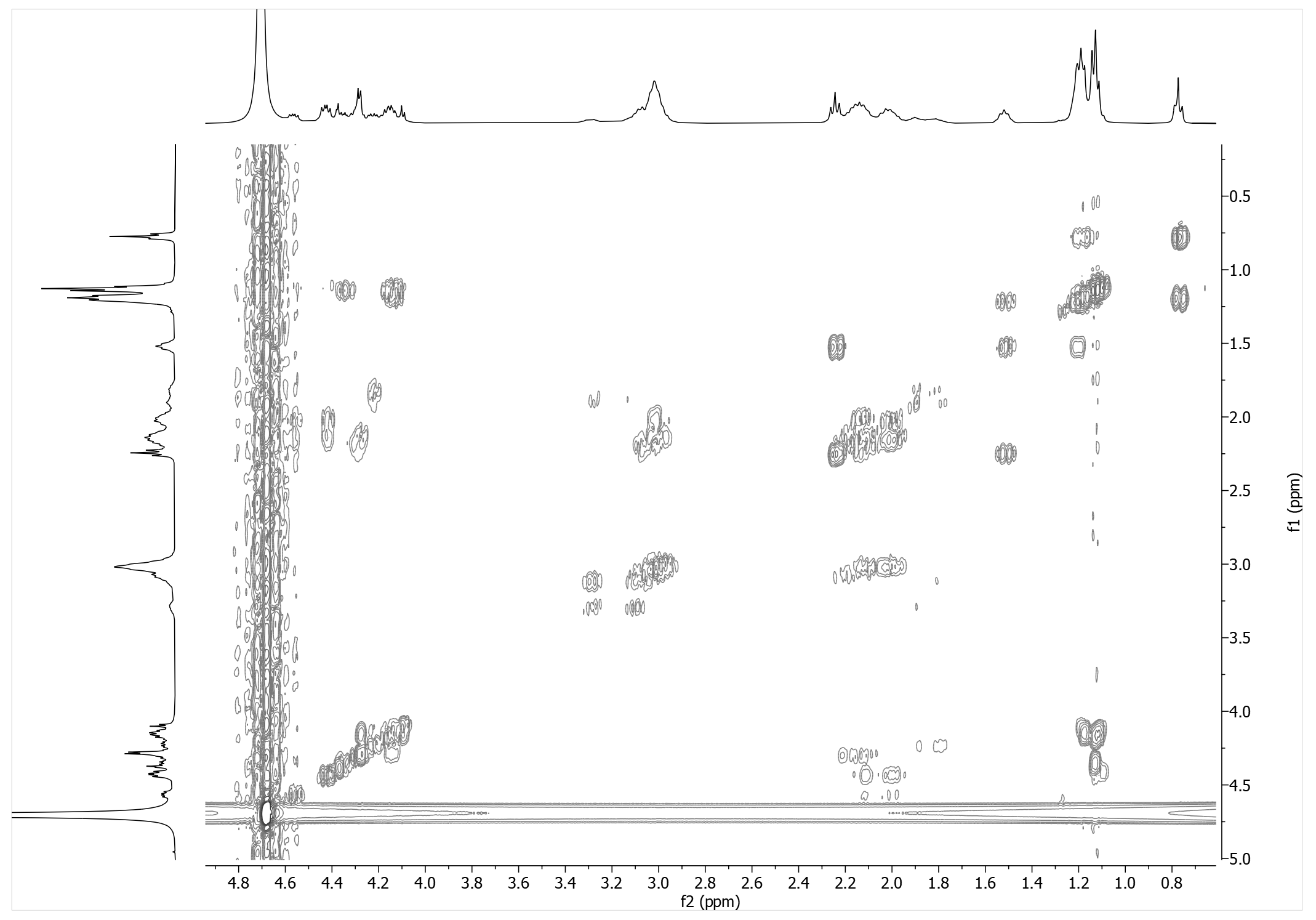


**Figure S3.** COSY of F230.


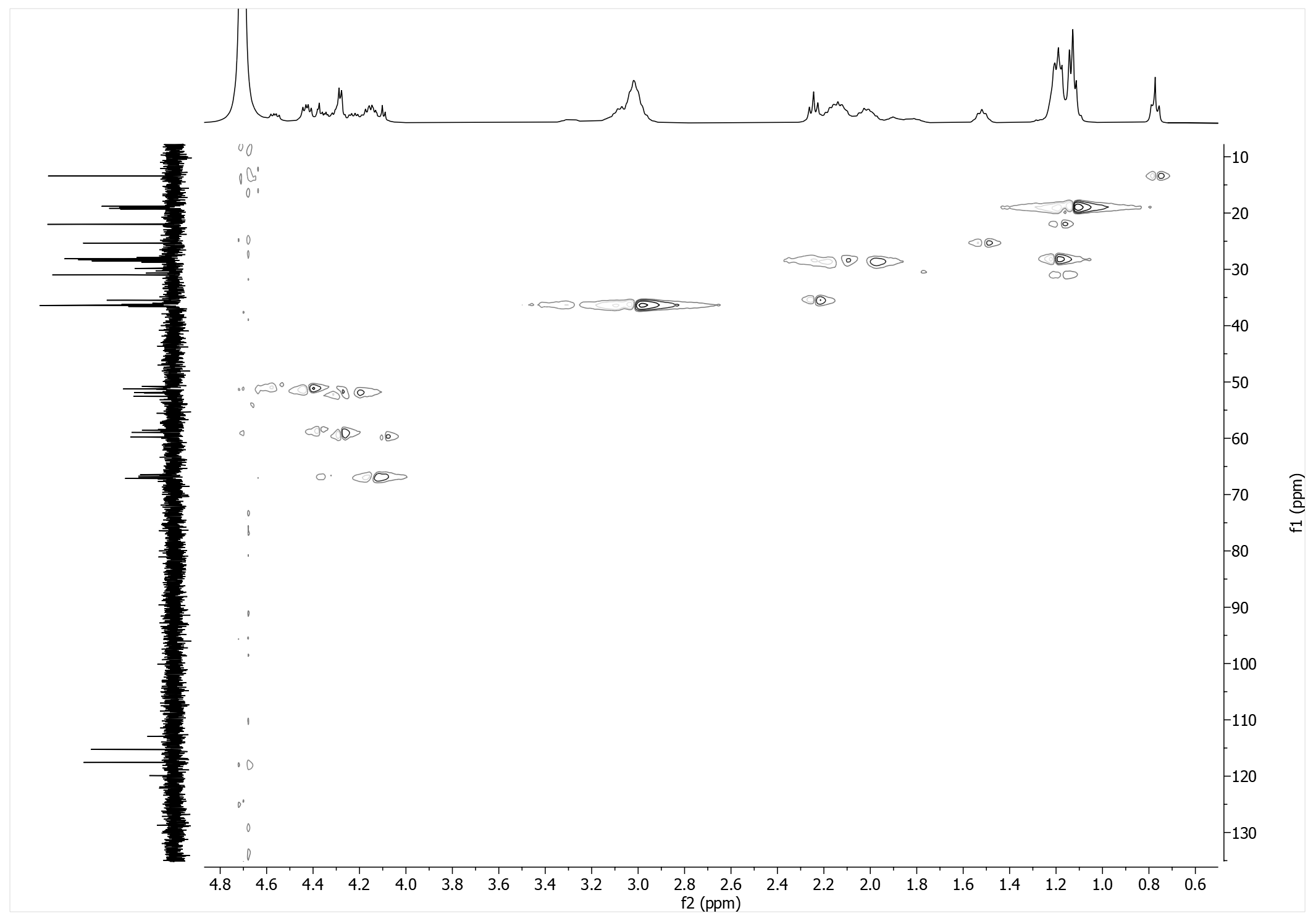


**Figure S4.** HSQC of F230.


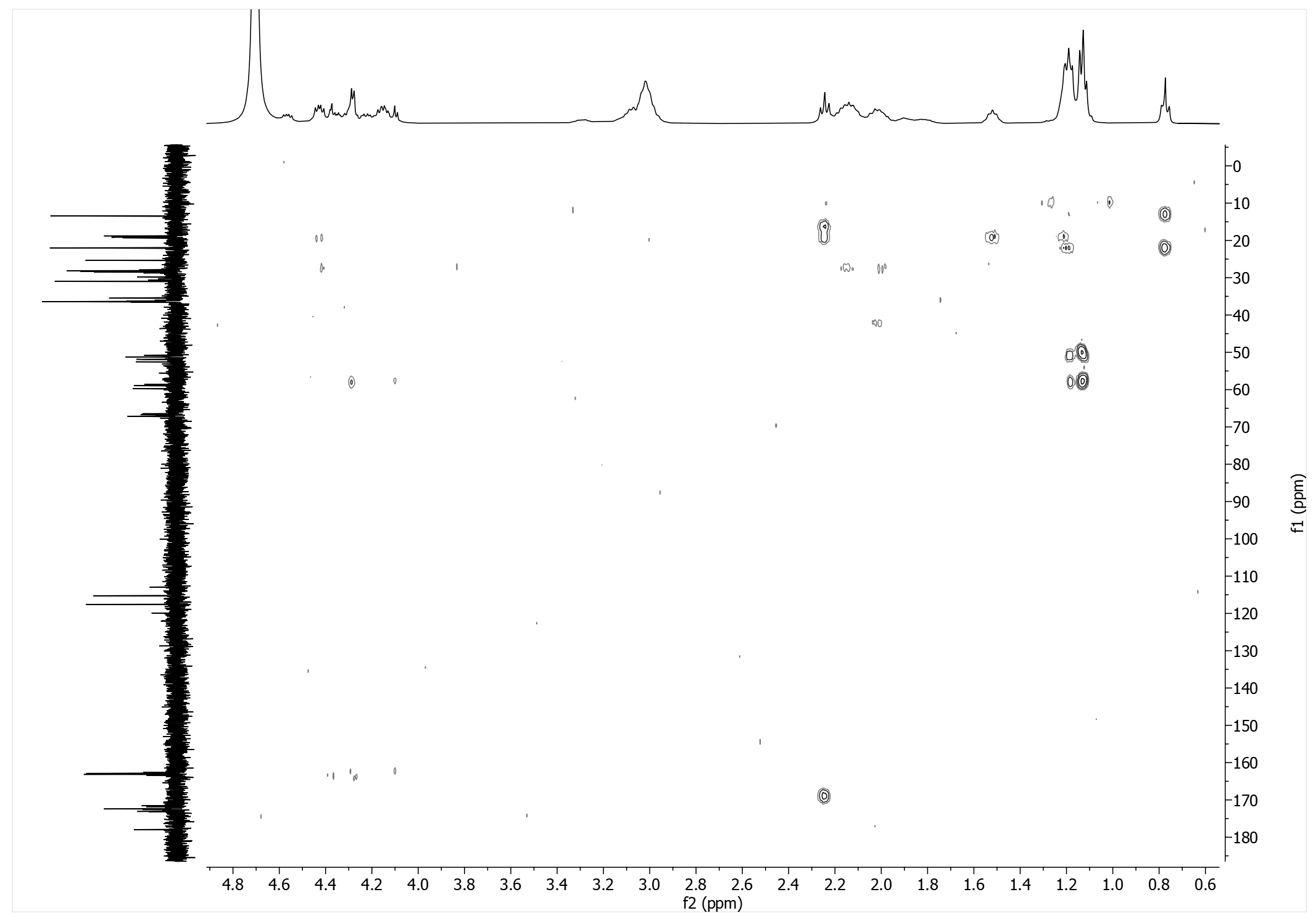


**Figure S5.** HMBC of F230.


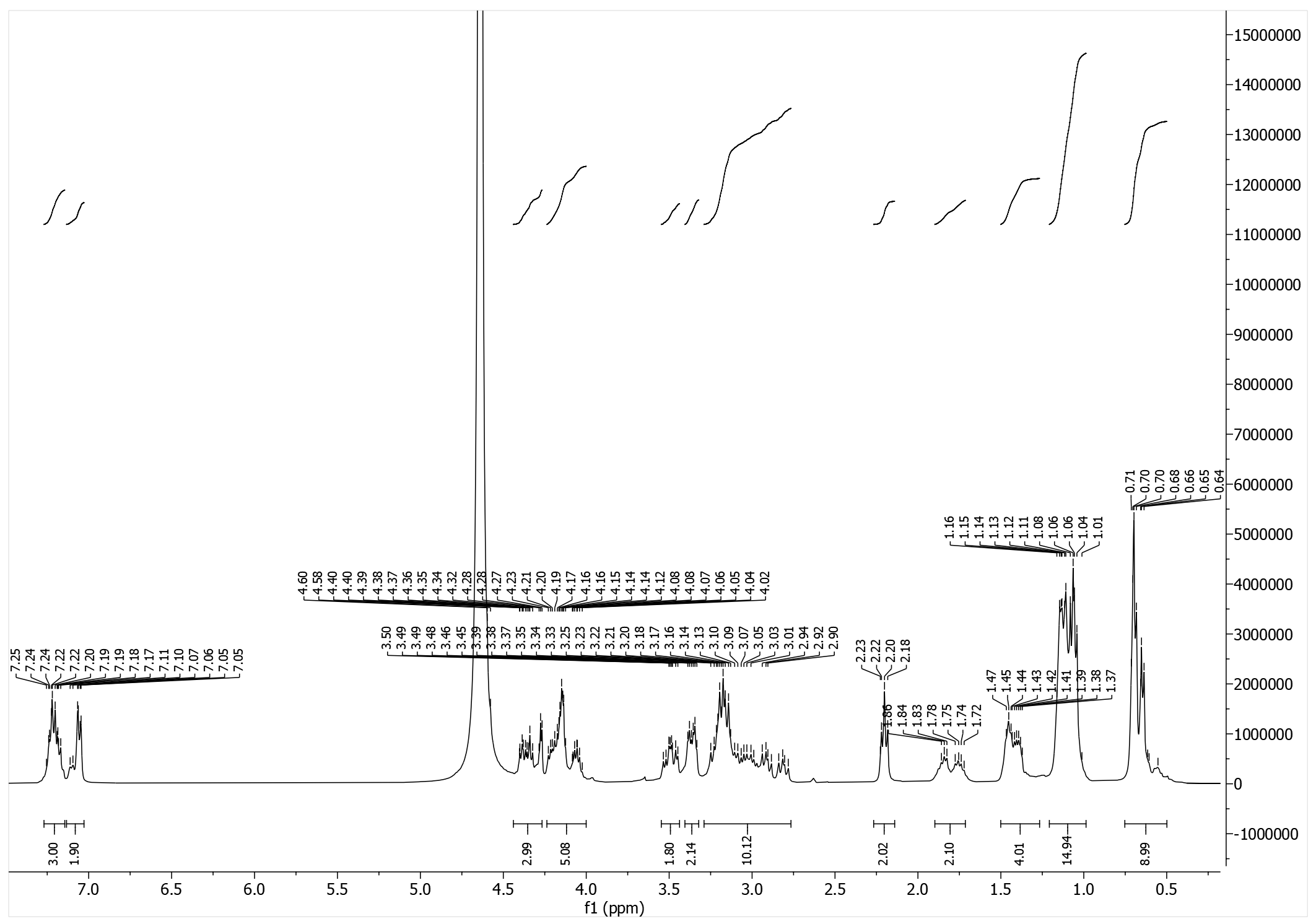


**Figure S6.** ^1^H NMR of Compound **1**.


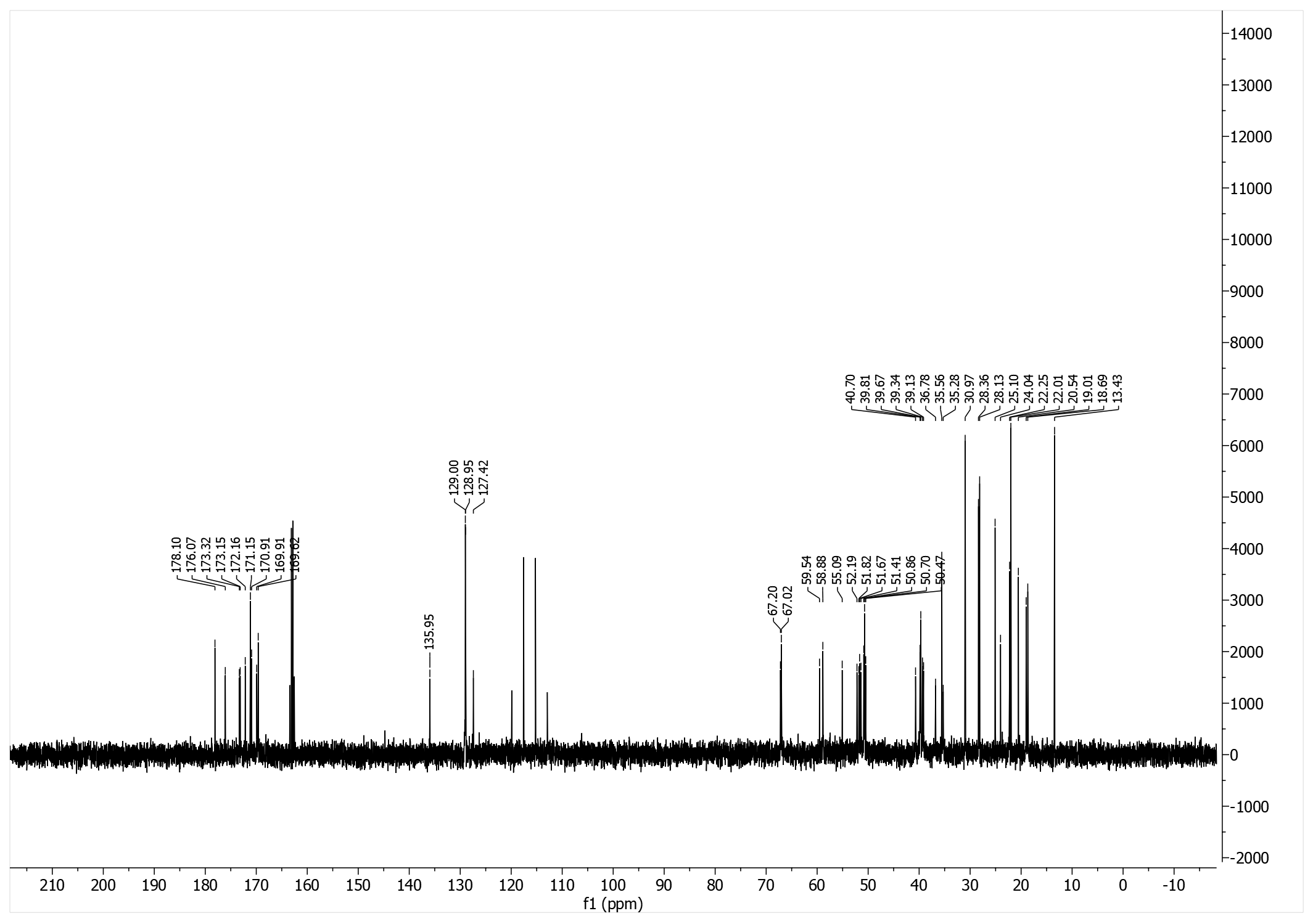


**Figure S7.** ^13^C NMR of Compound **1**.


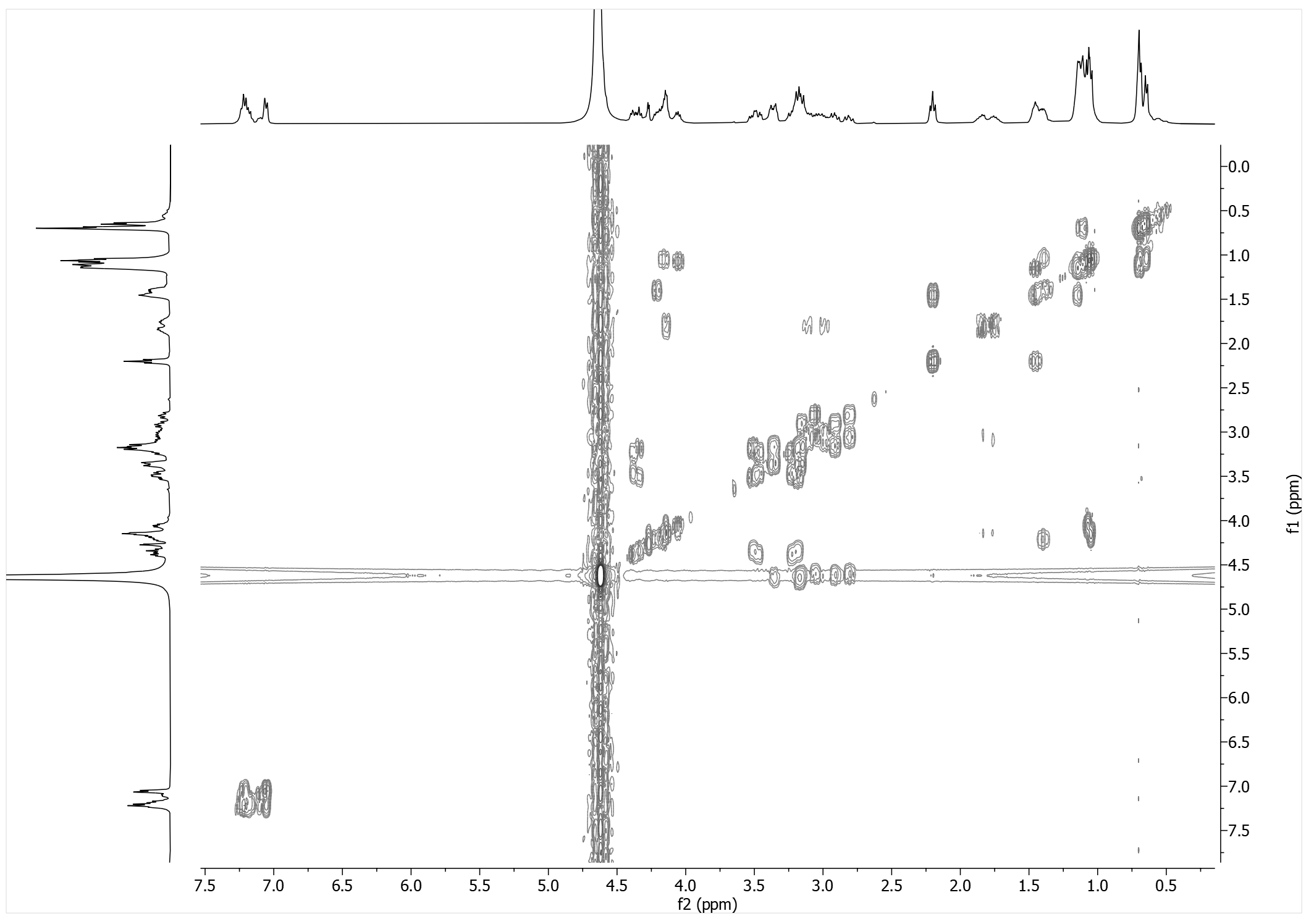


**Figure S8.** COSY of Compound **1**.


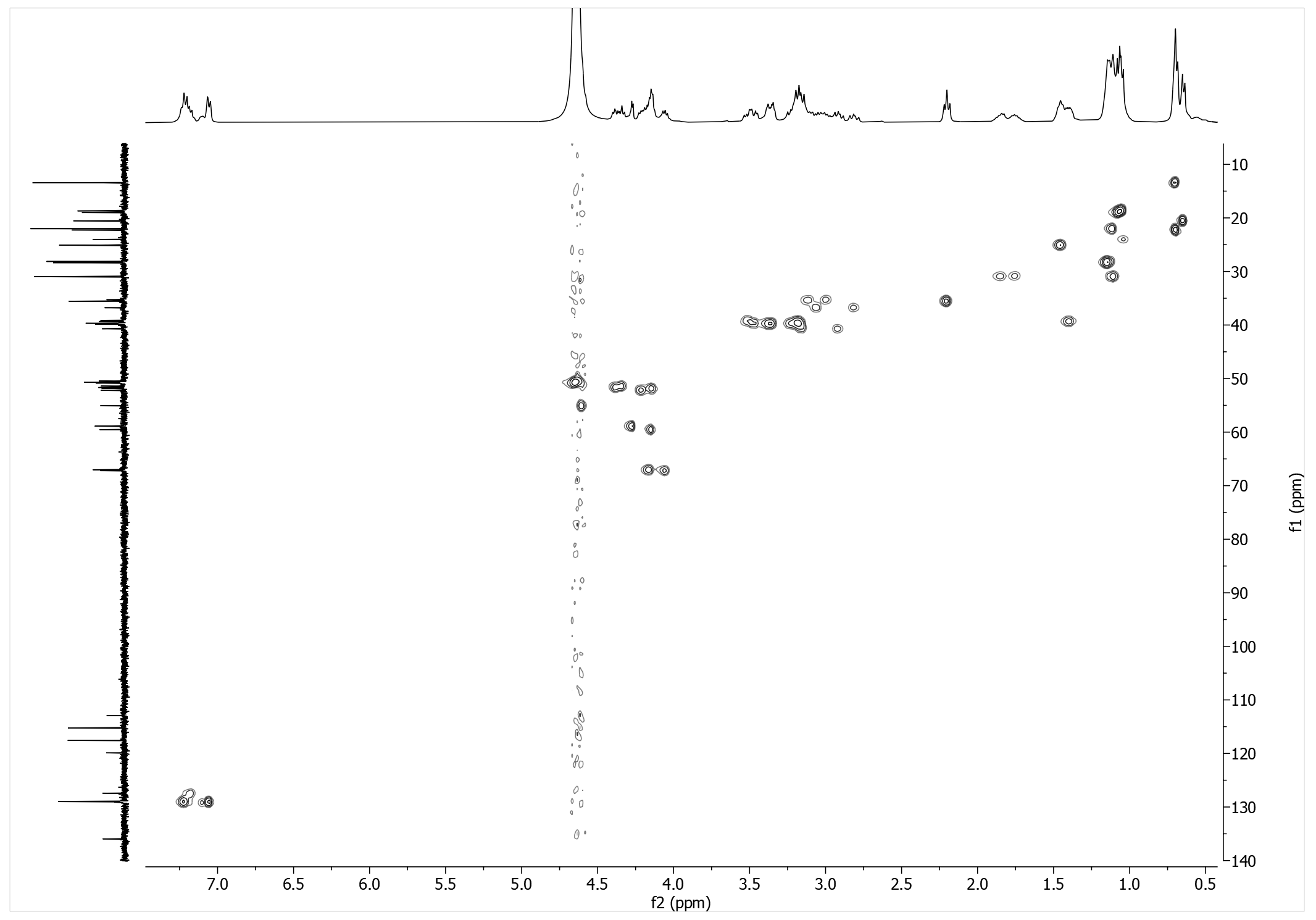


**Figure S9.** HSQC of Compound **1**.


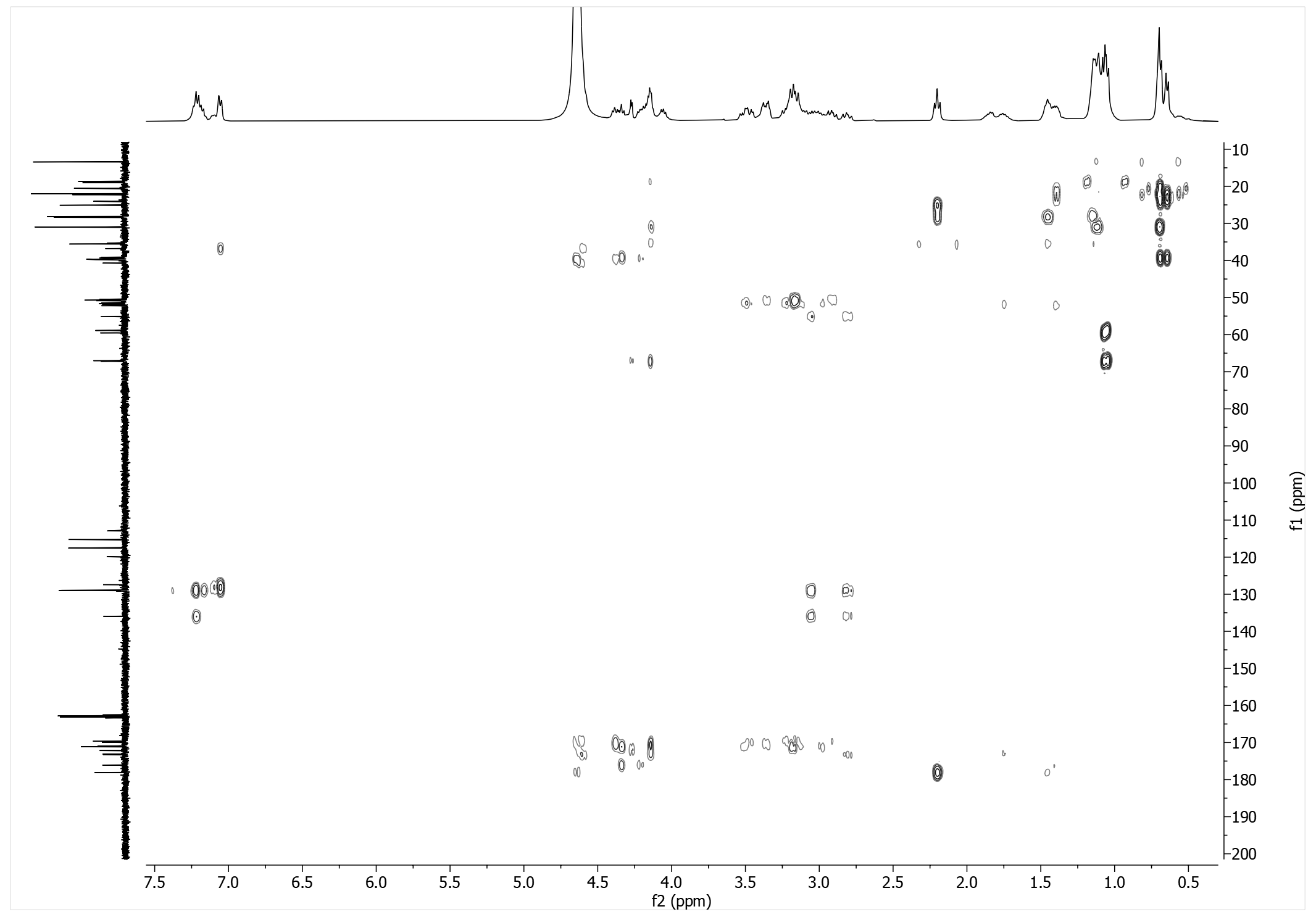


**Figure S10.** HMBC of Compound **1**.


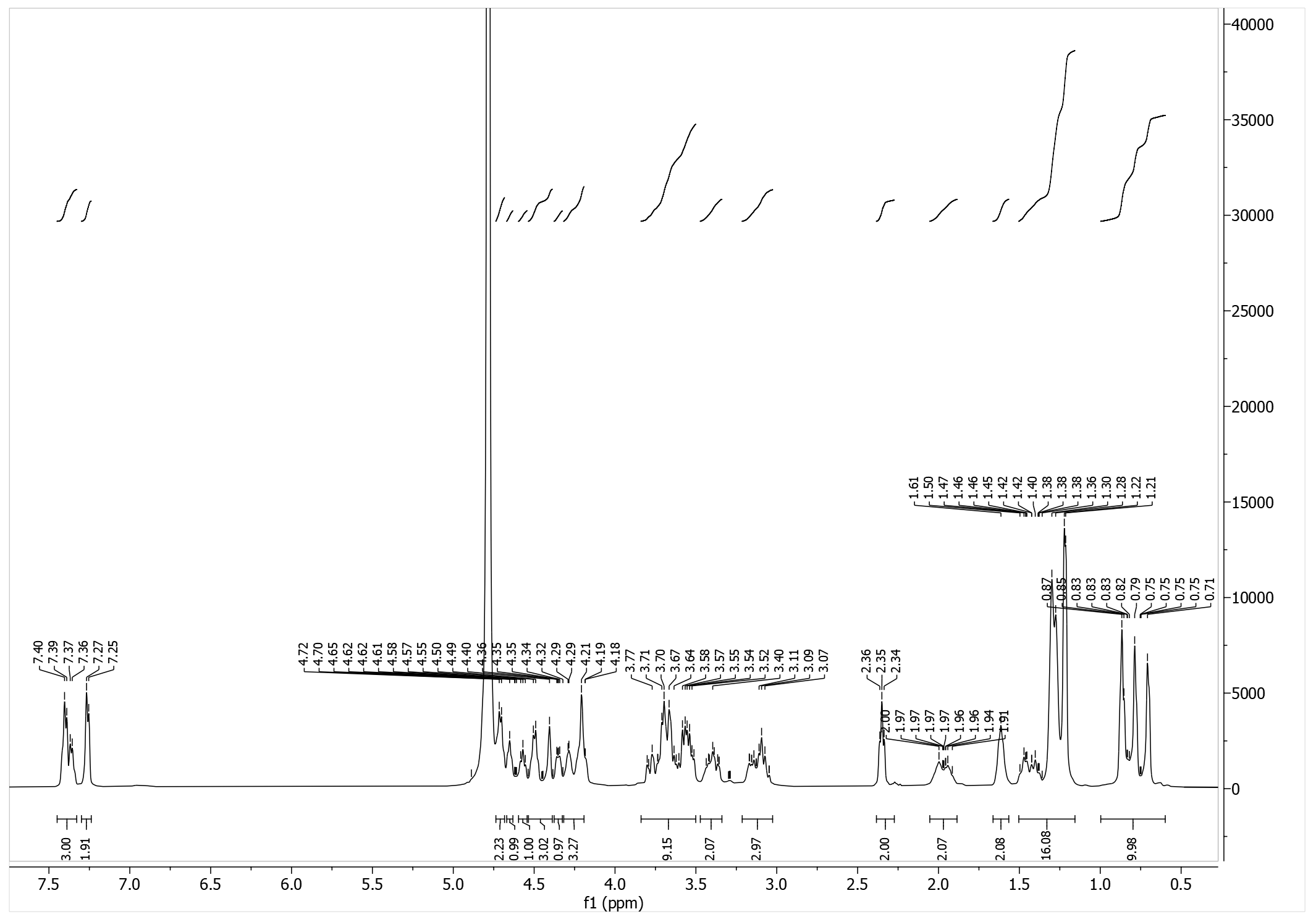


**Figure S11.** ^1^H NMR of Compound **2**.


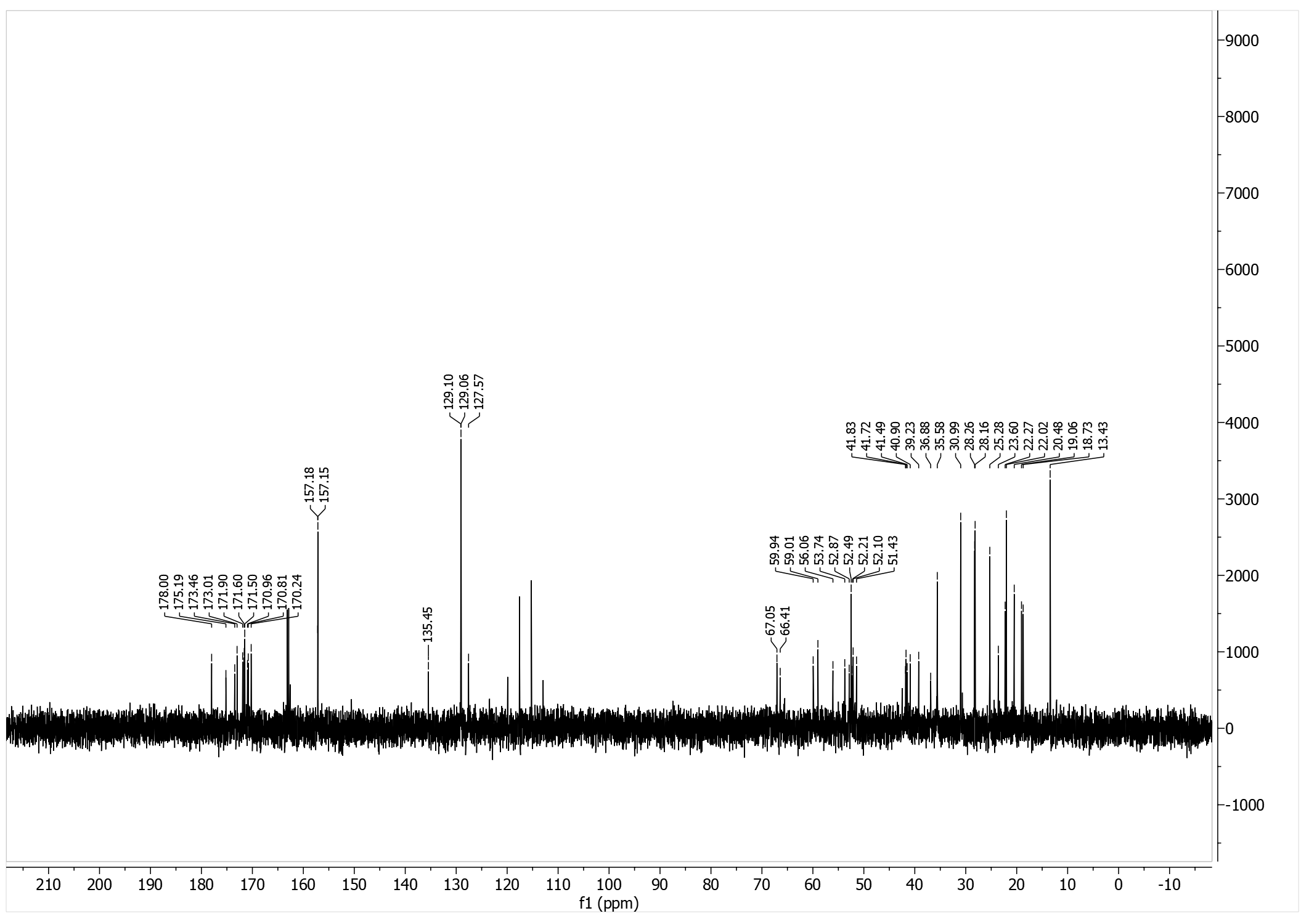


**Figure S12.** ^13^C NMR of Compound **2**.


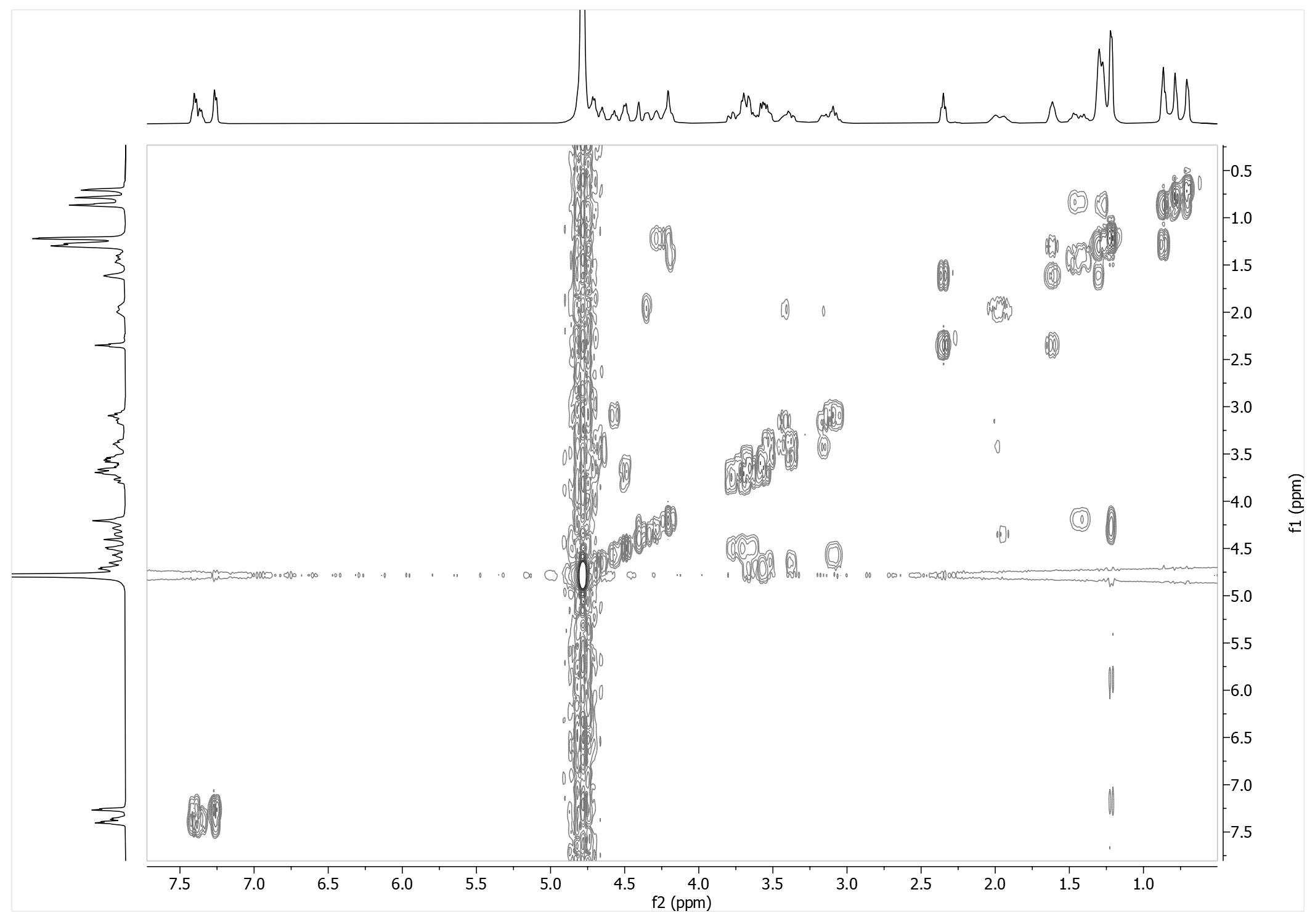


**Figure S13.** COSY of Compound **2**.


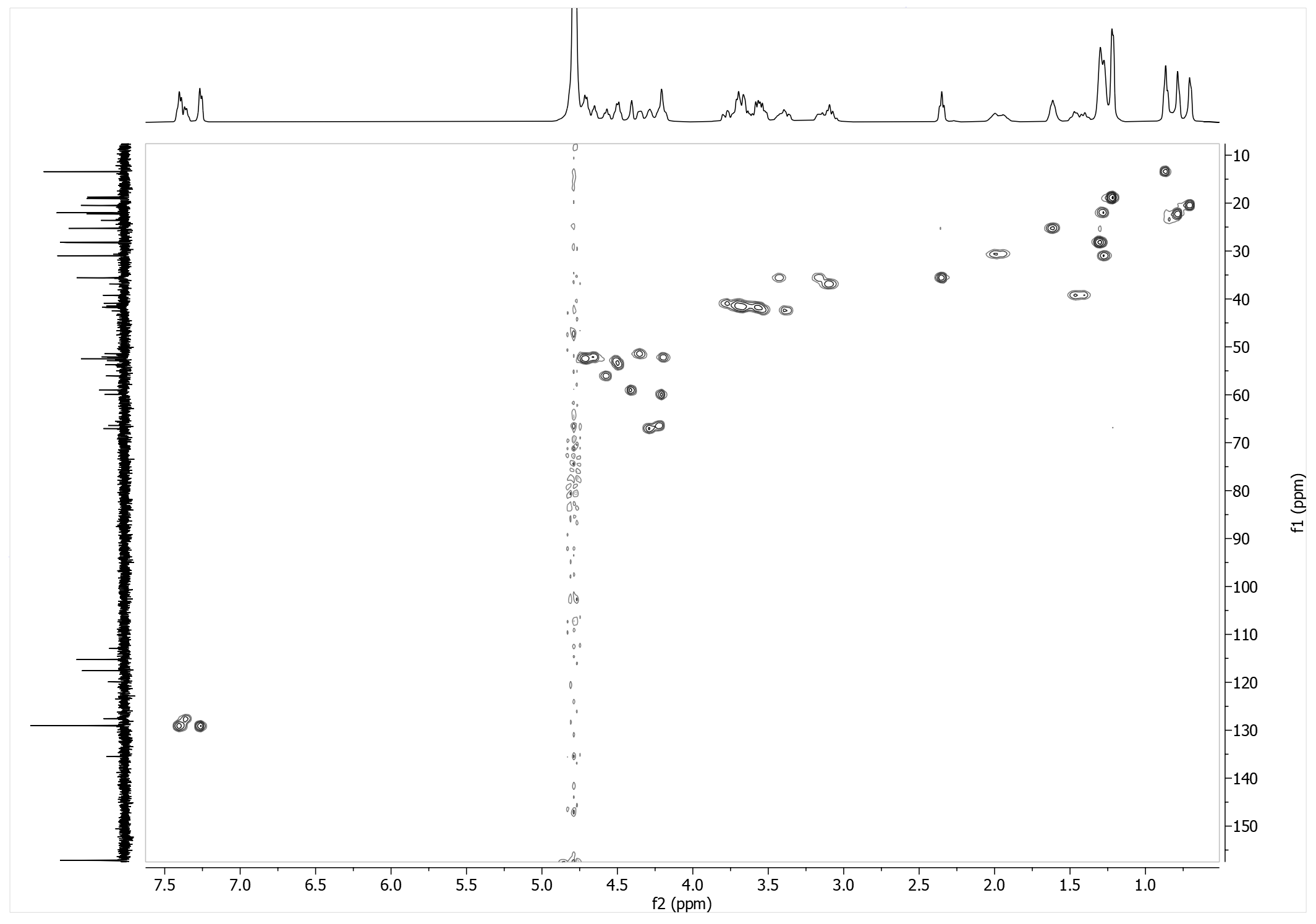


**Figure S14.** HSQC of Compound **2**.


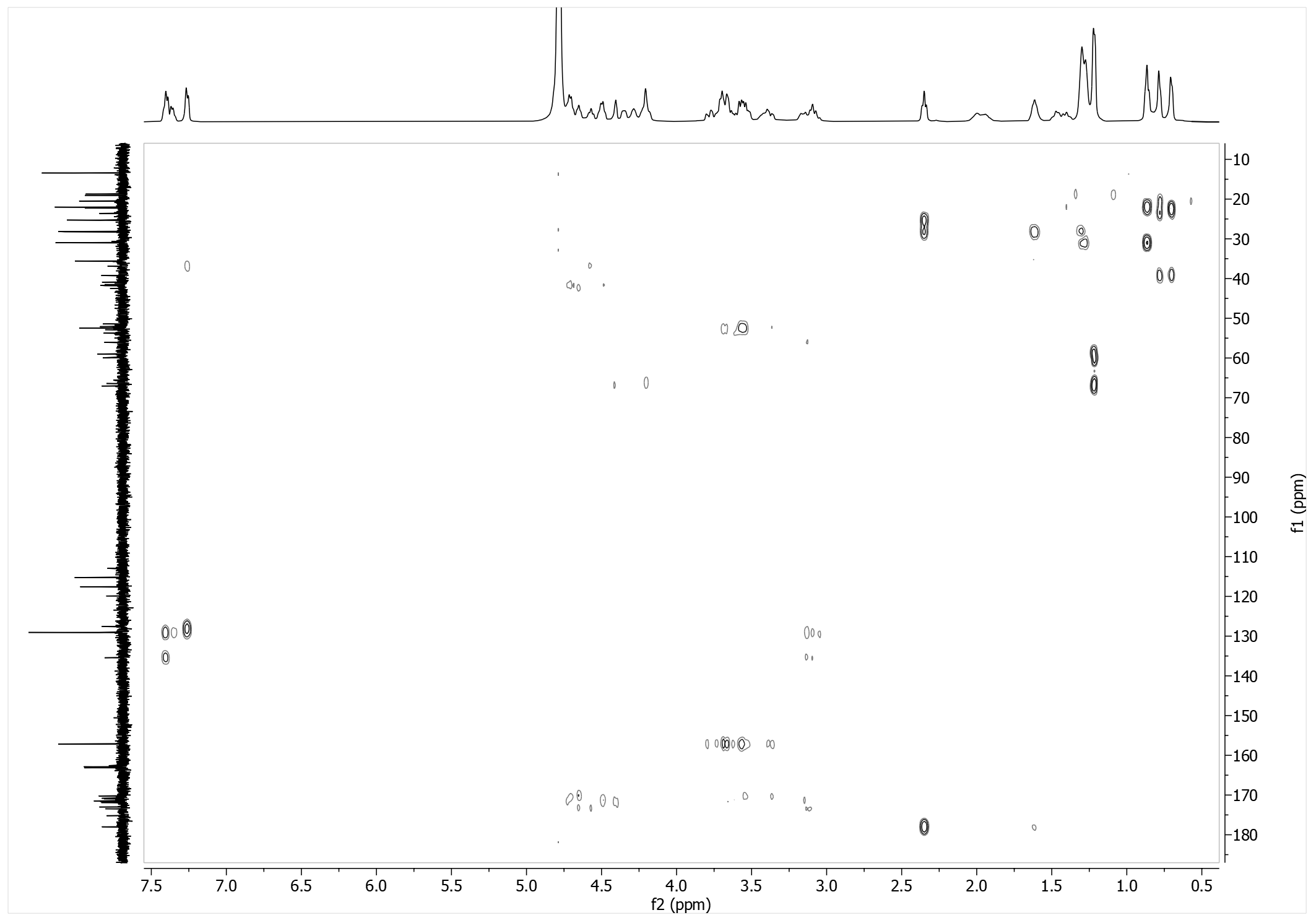


**Figure S15.** HMBC of Compound **2**.


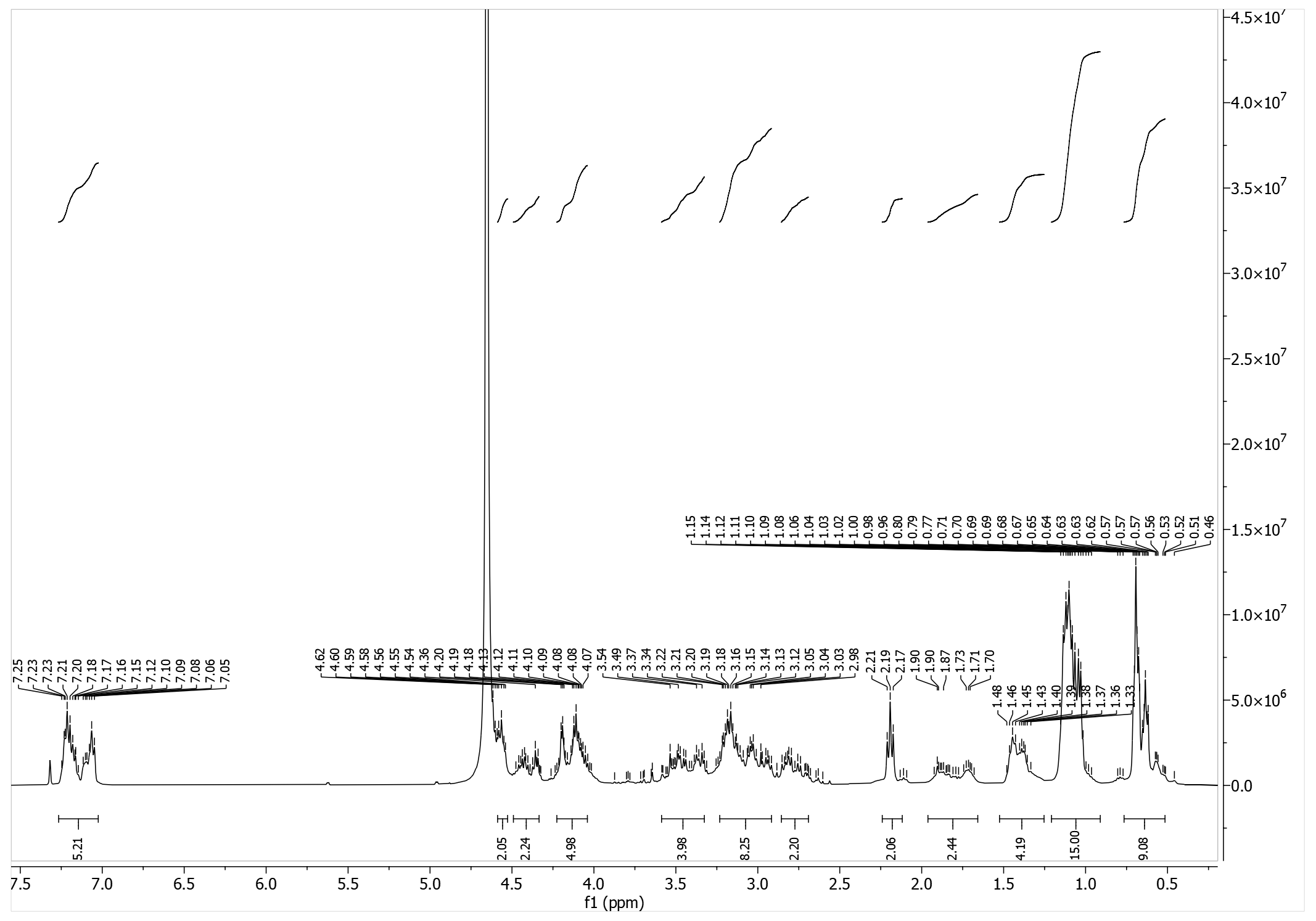


**Figure S16.** ^1^H NMR of Compound **3**.


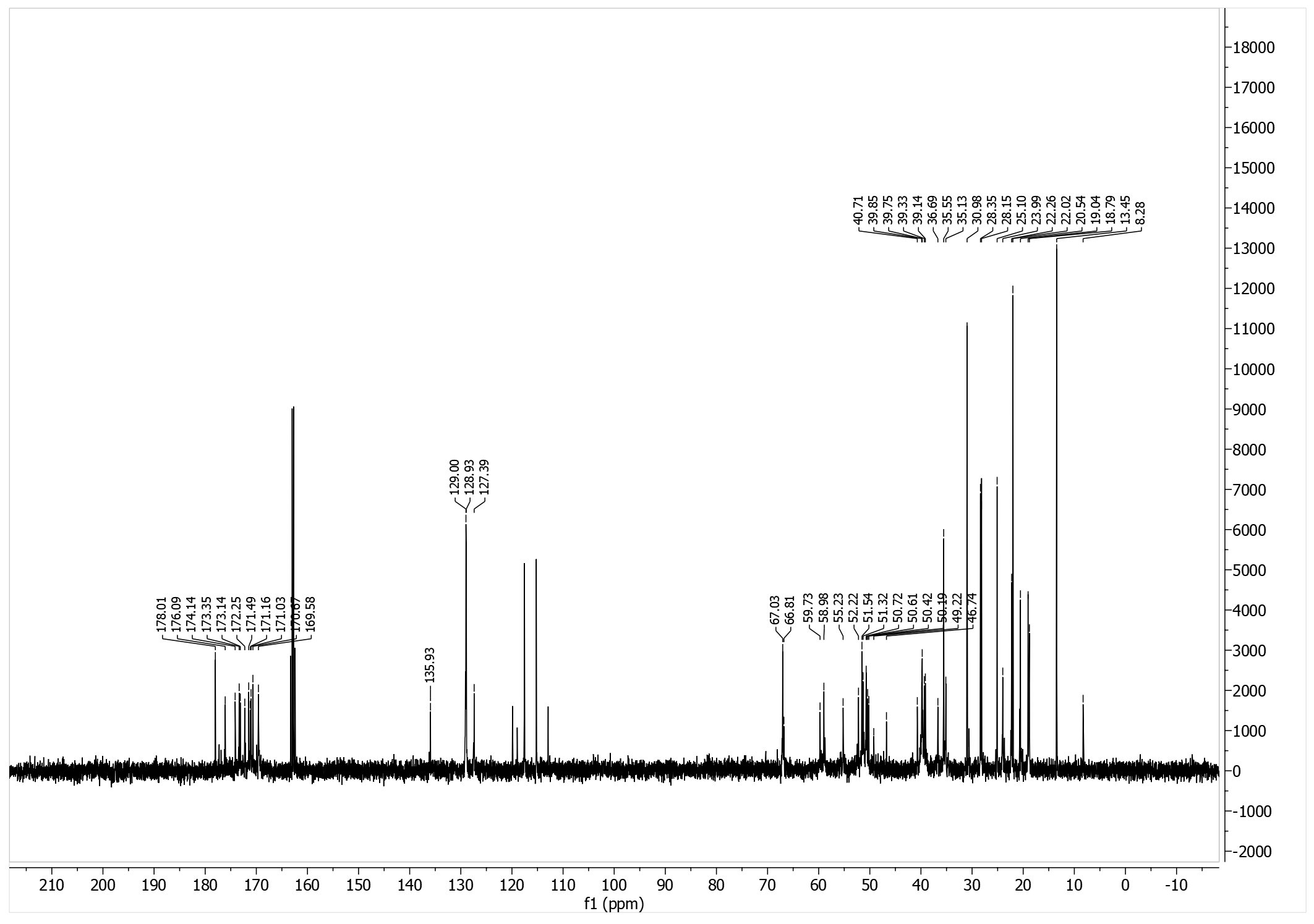


**Figure S17.** ^13^C NMR of Compound **3**.


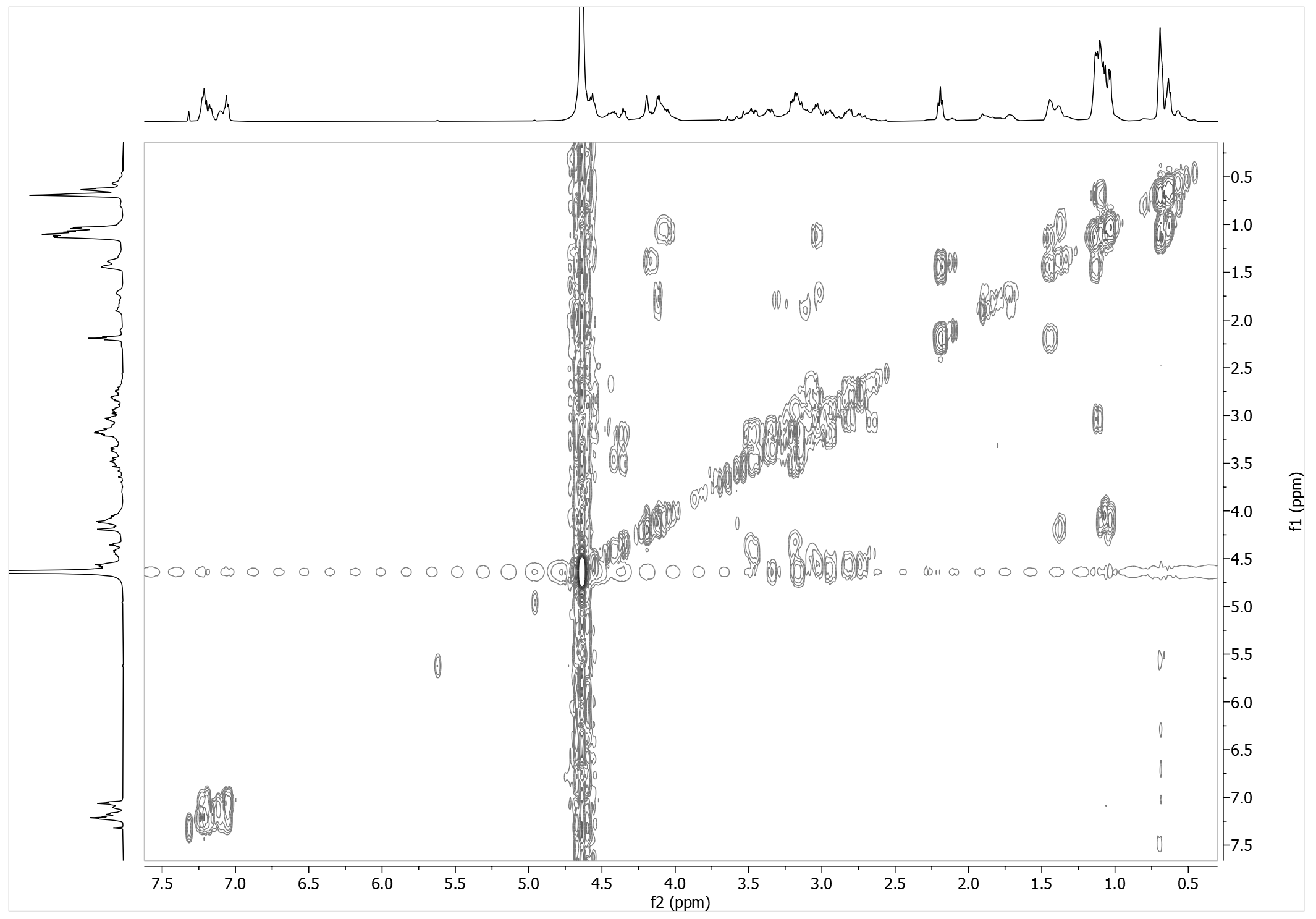


**Figure S18.** COSY of Compound **3**.


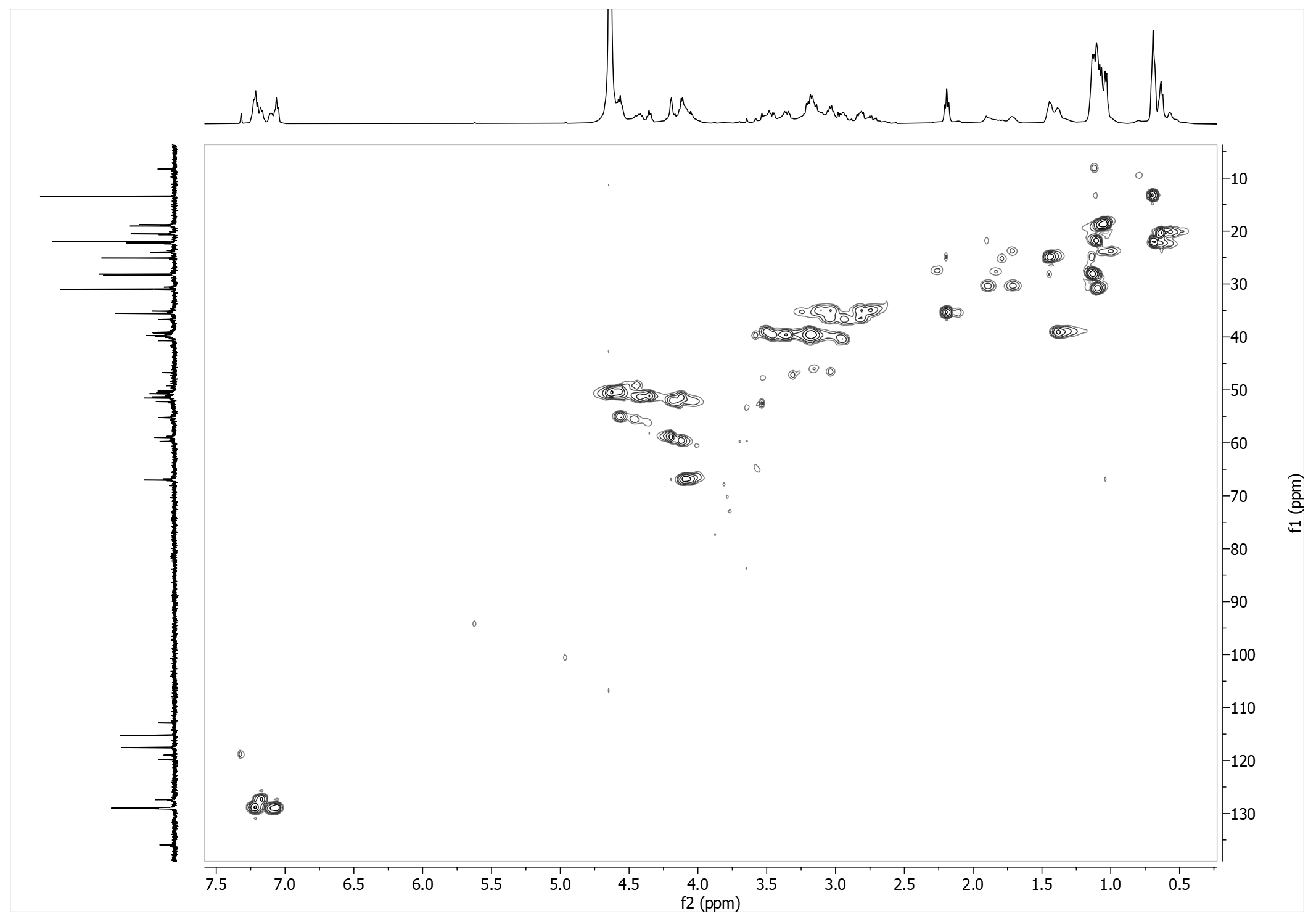


**Figure S19.** HSQC of Compound **3**.


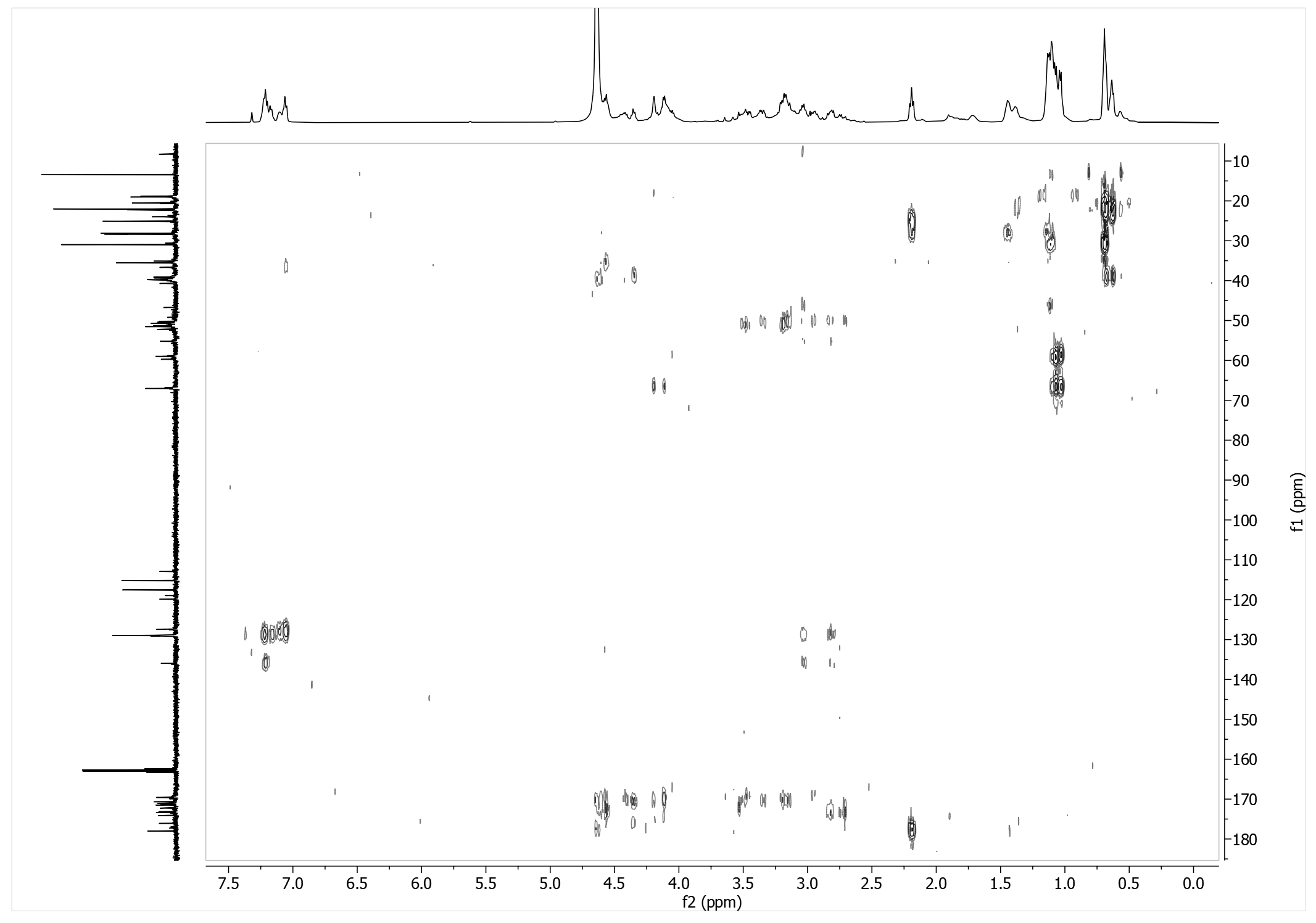


**Figure S20.** HMBC of Compound **3**.


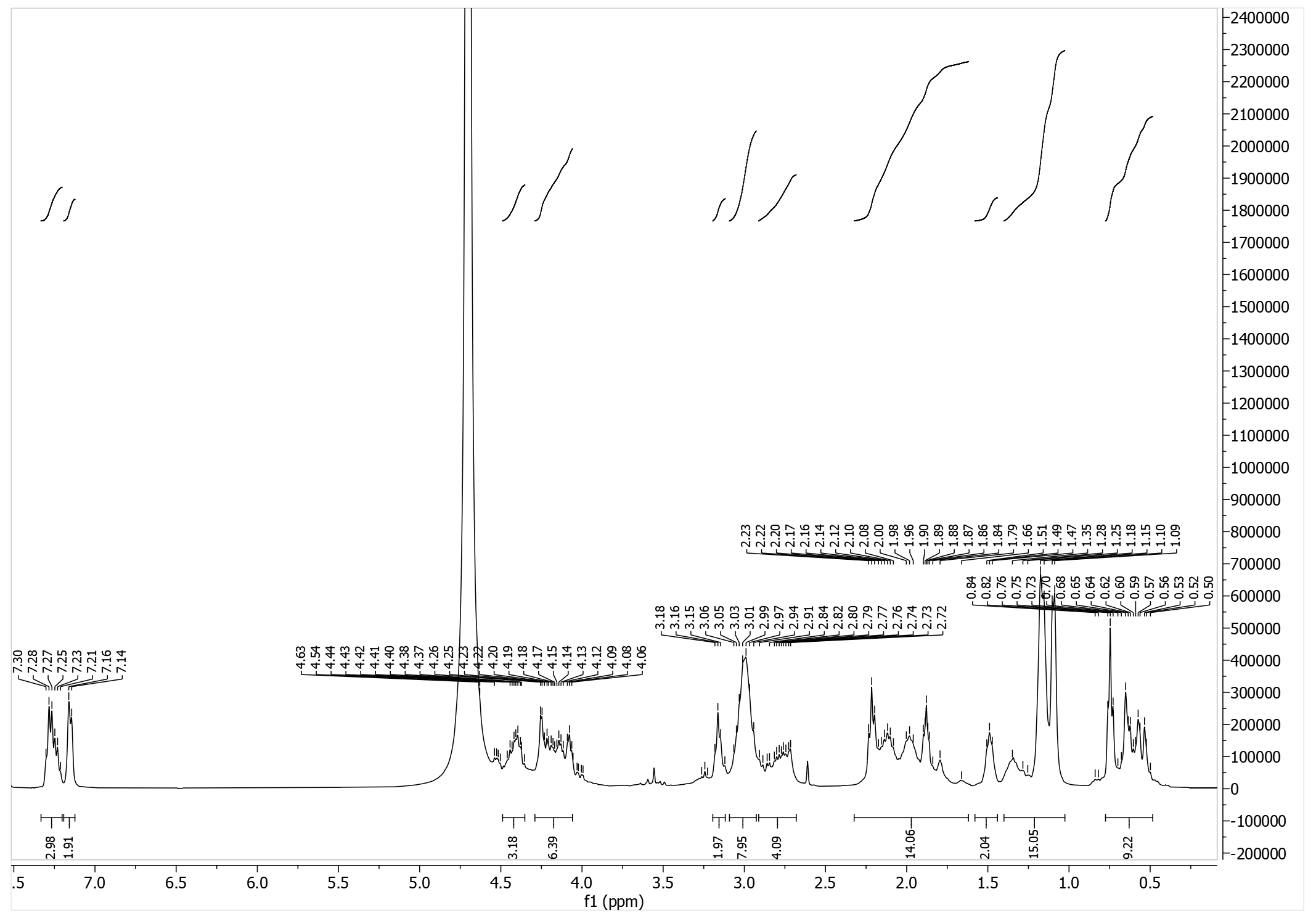


**Figure S21.** ^1^H NMR of Compound **4**.


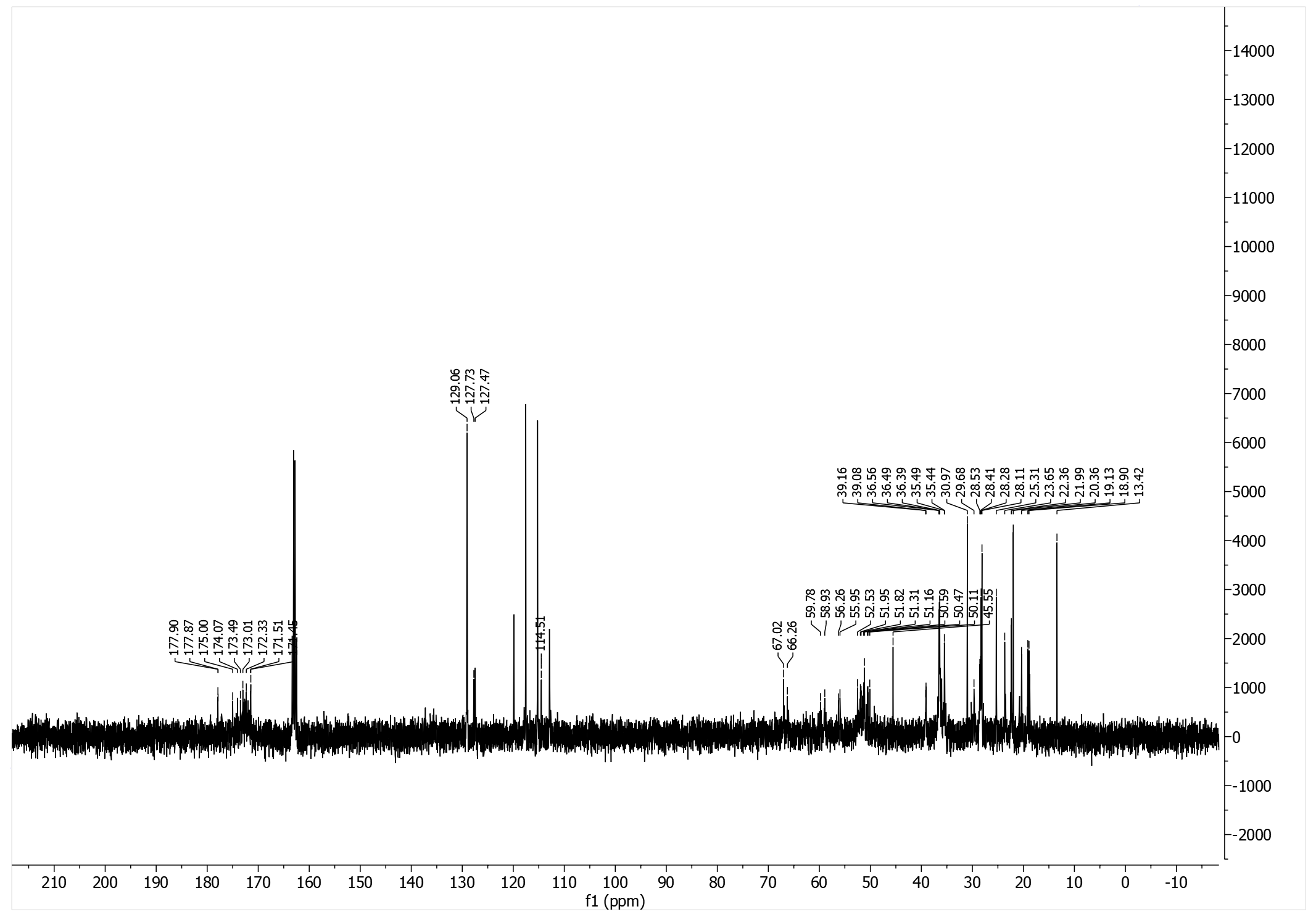


**Figure S22.** ^13^C NMR of Compound **4**.


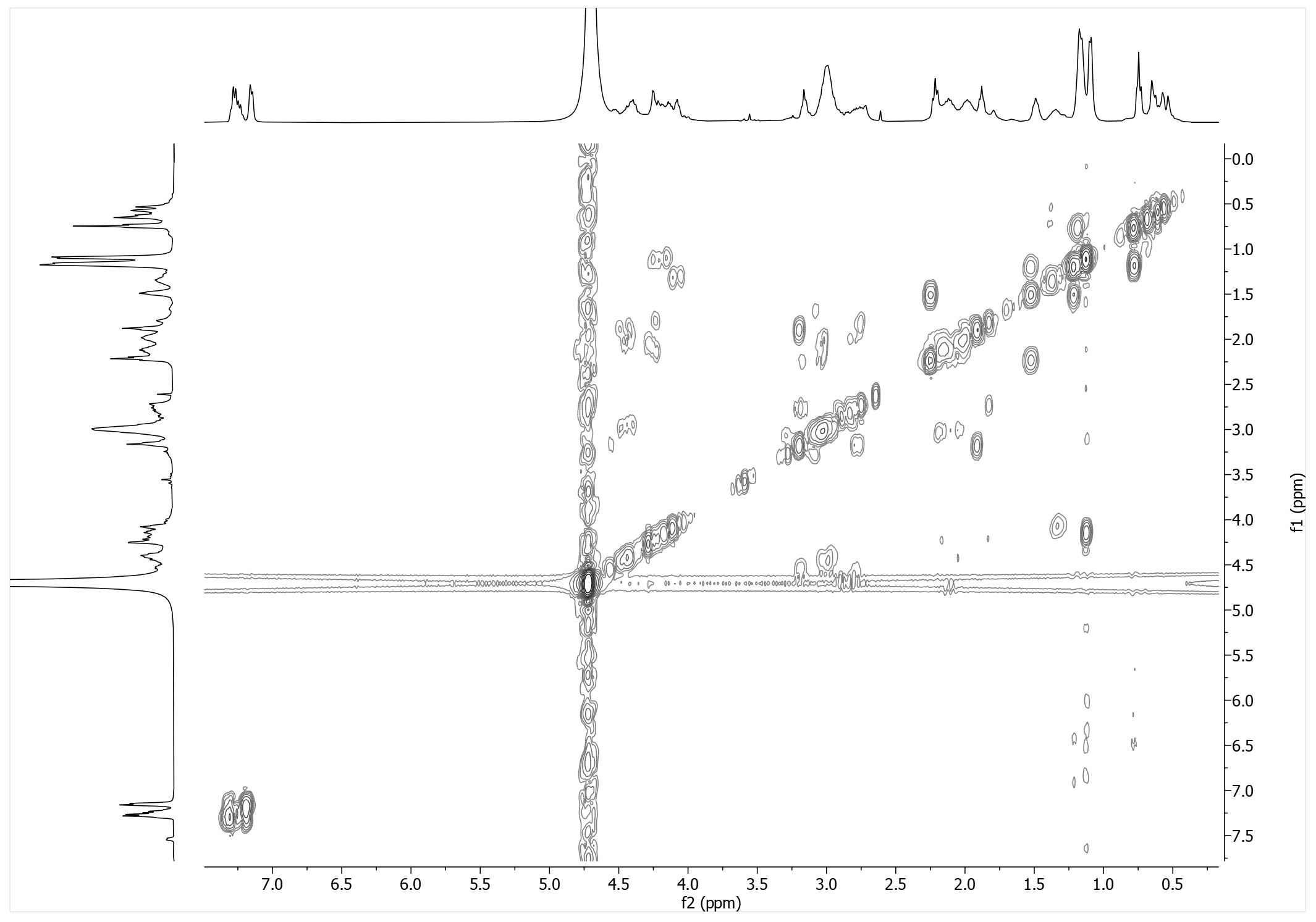


**Figure S23.** COSY of Compound **4**.


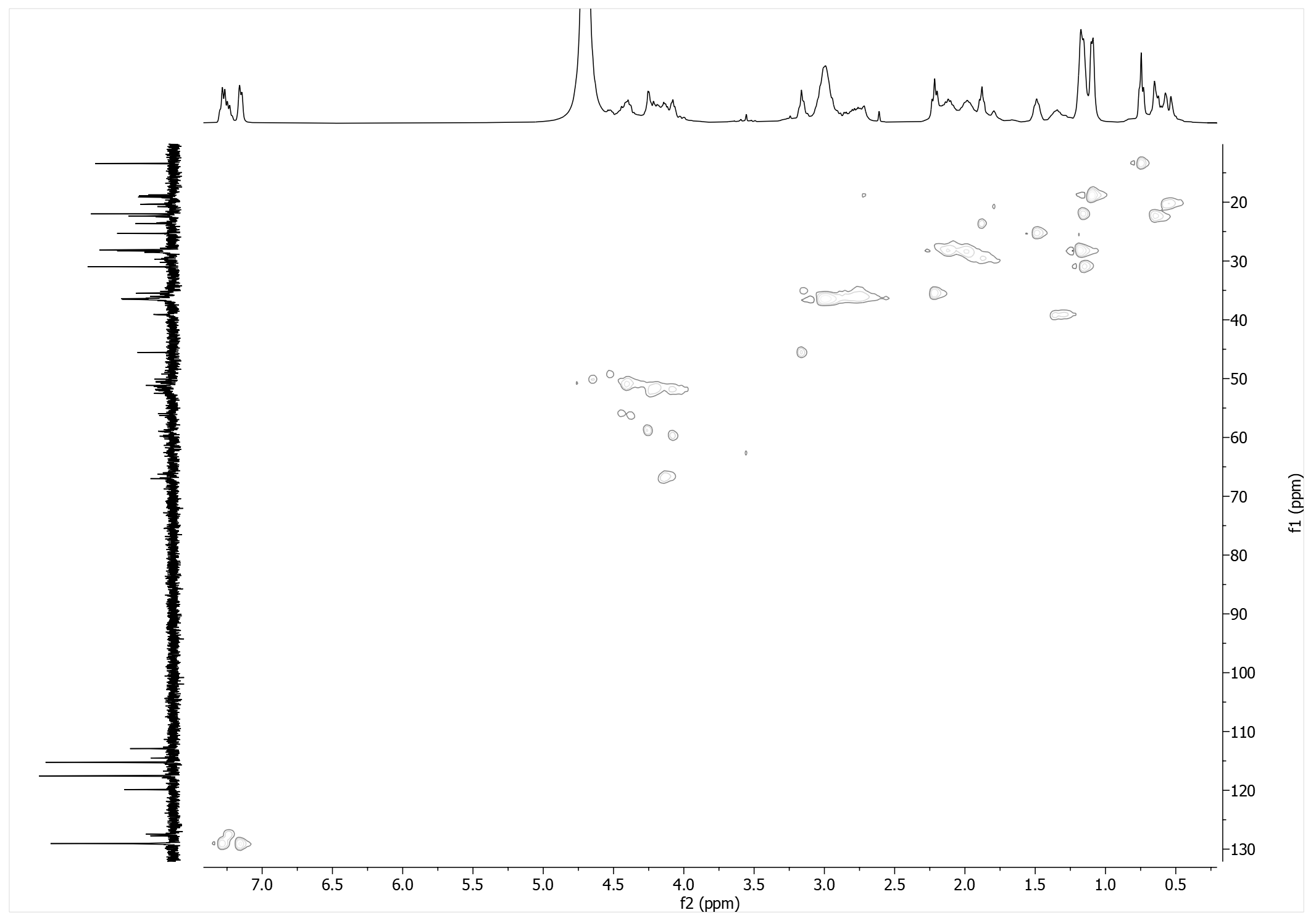


**Figure S24.** HSQC of Compound **4**.


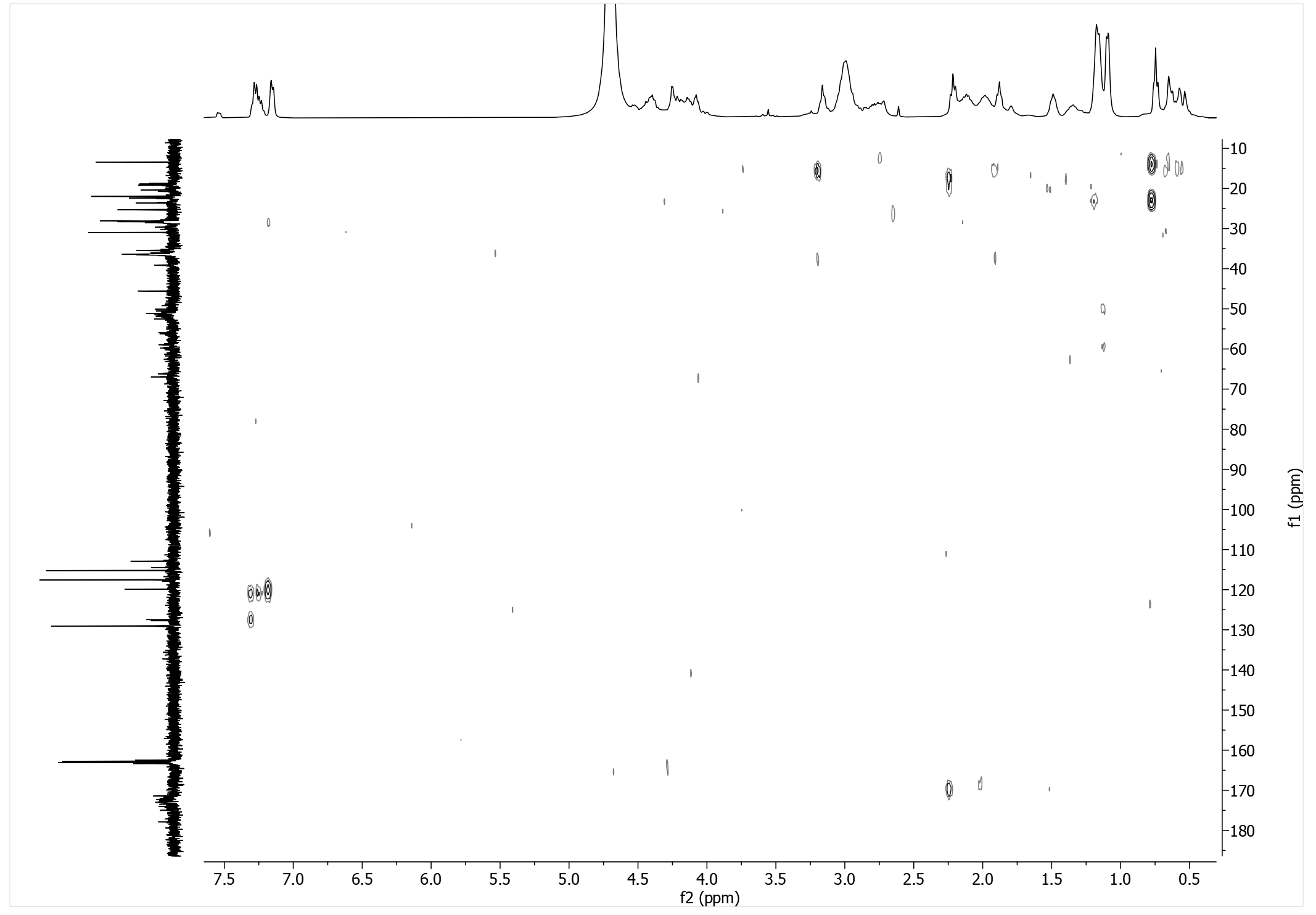


**Figure S25.** HMBC of Compound **4**.

**6. Resistance Phenotype of MDR Clinical Isolates**

Table S4. Resistance phenotype of MDR clinical isolates.

| *P. aeruginosa* | PTZ | A/C | ATM | FOX | CFZ | CTR | CPM | CTX | IMI | MER | DOR | ETP | CIP | MOX | TOB | GEN | AMK | TGC | DOX | ERC | OMC | CAM |
| --- | --- | --- | --- | --- | --- | --- | --- | --- | --- | --- | --- | --- | --- | --- | --- | --- | --- | --- | --- | --- | --- | --- |
| 259-96916 | 64 | >32 | 32 | >32 | >128 | >64 | >64 | 2048 | 32 | 1024 | >1024 | >32 | >16 | >16 | 256 | >32 | >64 | 32 | 32 | 8 | 64 | 1024 |
| 262-101856 | 64 | >32 | 32 | >32 | >128 | 64 | 32 | 128 | 32 | 32 | 16 | >32 | >16 | >16 | 1024 | >32 | >64 | 32 | 1024 | 8 | 64 | 2048 |
| 264-104354 | 256 | >32 | 64 | >32 | >128 | >64 | 32 | 2048 | 32 | 64 | 16 | >32 | >16 | >16 | 128 | >32 | 8 | 32 | 64 | 8 | 64 | 4096 |
| 101243 | 128 | >32 | 32 | >32 | >128 | >64 | 64 | ND | 16 | 16 | 16 | >32 | 1 | 8 | 128 | >32 | >64 | ND | 4 | ND | ND | 1 |
| 114228 | ND | ND | 32 | ND | ND | ND | ND | 128 | ND | 8 | 8 | ND | ND | ND | 2 | ND | ND | ND | 16 | 16 | 128 | ND |

| *A. baumannii* | PTZ | FOX | CFZ | CPM | CTX | C/T | IMI | MER | CIP | MOX | TOB | GEN | AMK | TGC | DOX | ERC | OMC | CAM |
| --- | --- | --- | --- | --- | --- | --- | --- | --- | --- | --- | --- | --- | --- | --- | --- | --- | --- | --- |
| AB027 | 512 | ND | >128 | >128 | >256 | >16 | 32 | 16 | >16 | 8 | ND | 32 | >64 | 4 | ND | 0.5 | 1 | 128 |
| AB031 | 4 | ND | >128 | 4 | 16 | >16 | 0.25 | 1 | 0.25 | 0.12 | ND | <0.5 | 2 | 8 | ND | 0.25 | 2 | 128 |
| 92247 | <1 | 32 | 128 | 4 | ND | 2 | ND | 4 | ≤0.06 | ND | ND | ND | <1 | 0.25 | ND | ND | ND | ND |
| 110193 | ND | ND | ND | 64 | ND | ND | ND | ND | ≤1 | ≤1 | 1 | 2 | ND | ND | 16 | ND | ND | 128 |

| *E. coli* | PTZ | A/C | ATM | FOX | CFZ | CPM | C/T | IMI | MER | ETP | CIP | MOX | TOB | GEN | AMK | TGC | DOX | ERC | OMC | CAM |
| --- | --- | --- | --- | --- | --- | --- | --- | --- | --- | --- | --- | --- | --- | --- | --- | --- | --- | --- | --- | --- |
| 94393 (mcr-1 +) | ≤1 | 4 | ≤0.12 | 4 | 1 | ≤0.25 | 0.25 | 0.25 | ≤0.03 | ≤0.03 | 0.5 | 1 | ≤0.5 | ≤0.5 | 2 | 0.25 | 4 | 0.5 | 4 | 4 |
| 94474 (mcr-1 +) | 16 | >32 | ≤0.12 | 16 | 4 | ≤0.25 | 0.5 | 0.25 | ≤0.03 | ≤0.03 | >16 | 16 | 32 | 16 | 2 | 1 | >32 | 1 | 16 | 4 |
| 107115 | >512 | >32 | >64 | >32 | >128 | >64 | >16 | 8 | 32 | >32 | >16 | 16 | 8 | >32 | 2 | 0.25 | >32 | 0.125 | 4 | 512 |
| 131629 | ND | ND | >32 | ND | ND | 16 | ND | ND | ND | ND | 64 | 32 | 2 | 4 | ND | ND | 4 | ND | ND | 2 |

| *K. pneumoniae* | PTZ | A/C | ATM | FOX | CFZ | CPM | C/T | IMI | MER | ETP | CIP | MOX | TOB | GEN | AMK | TGC | DOX | ERC | OMC | CAM |
| --- | --- | --- | --- | --- | --- | --- | --- | --- | --- | --- | --- | --- | --- | --- | --- | --- | --- | --- | --- | --- |
| 113250 | 4 | 4 | ≤0.12 | 1 | 1 | 1 | 2 | 0.25 | ≤0.03 | ≤0.03 | ≤0.06 | ≤0.06 | ≤0.5 | ≤0.5 | ≤1 | ND | 2 | 1 | 2 | 4 |
| 116381 | 8 | 16 | 16 | 16 | >128 | 16 | 1 | 0.5 | ≤0.03 | 0.12 | >16 | >16 | 4 | ≤0.5 | ≤1 | 1 | 1 | 0.5 | 4 | >512 |

| *E. cloacae* | PTZ | A/C | ATM | FOX | CFZ | CPM | C/T | IMI | MER | ETP | CIP | MOX | TOB | GEN | AMK | TGC | DOX | ERC | OMC | CAM |
| --- | --- | --- | --- | --- | --- | --- | --- | --- | --- | --- | --- | --- | --- | --- | --- | --- | --- | --- | --- | --- |
| 117029 | 2 | 16 | ≤0.12 | >32 | >128 | ≤0.25 | 0.25 | 0.25 | ≤0.03 | ≤0.03 | ≤0.06 | ≤0.06 | 2 | ≤0.5 | 2 | 0.5 | >32 | 0.5 | 4 | 8 |
| 121187 | 1 | 8 | ≤0.12 | >32 | 32 | 0.25 | ND | ND | 0.06 | ND | 0.25 | 1 | 32 | >32 | 1 | ND | >32 | ND | ND | ND |

PTZ: piperacillin-tazobactam, A/C: amoxicillin-clavulanic acid, ATM: aztreonam, FOX: cefoxitin, CFZ: cefazolin, CTR: ceftriaxone, CPM: cefepime, CTX: cefotaxime, C/T: ceftolozane-tazobactam, IMI: imipenem, MER: meropenem, DOR: doripenem, ETP: ertapenem, CIP: ciprofloxacin, MOX: moxifloxacin, TOB: tobramycin, GEN: gentamicin, AMK: amikacin, TGC: tigecycline, DOX: doxycycline, ERC: eravacycline, OMC: omadacyline, CAM: chloramphenicol, ND: not determined.
